# Efficacy of five different traditional Chinese medicine injections in acute upper respiratory tract infection in children: a network meta-analysis and systematic review

**DOI:** 10.3389/fped.2024.1358639

**Published:** 2024-06-10

**Authors:** Xinyi Guo, Changxing Liu, Qiong Zhao, Sajiyue Huang

**Affiliations:** ^1^ College of Clinical Medicine, Chengdu University of Traditional Chinese Medicine, Chengdu, China; ^2^ Department of Pediatrics, Hospital of Chengdu University of Traditional Chinese Medicine, Chengdu, China; ^3^ Graduate Student, Heilongjiang University of Traditional Chinese Medicine, Heilongjiang, China

**Keywords:** acute upper respiratory tract infection in children, traditional Chinese medicine, randomized controlled trial, network meta-analysis, injection

## Abstract

**Background:**

Acute upper respiratory tract infection (AURI) includes infections caused by a variety of pathogens and is one of the most common diseases in children. Traditional Chinese medicine (TCM) injections are widely used for treating AURI in clinical practice, but their efficacy is unclear because of the lack of clear evidence. In this study, a network meta-analysis (NMA) was used to evaluate the efficacy and safety of TCM injections in the treatment of AURI and to provide a reference for clinical treatment.

**Methods:**

Eight databases were searched, namely, PubMed, Embase, the Cochrane Library, Web of Science, SinoMed, China National Knowledge Infrastructure (CNKI), the Wanfang database, and the Chinese Scientific Journal database (VIP). The search time period was from 1 January 2013 to 1 November 2023. Randomized controlled trials of herbal injections for treating AURI were searched. The Cochrane Risk of Bias 2.0 tool was used to assess the quality of these studies. Review Manager 5.4 and Stata 15.0 were used for the NMA.

**Results:**

A total of 81 papers involving 11,736 patients were included. These involved five different TCM injections, namely, Xiyanping injection (XYPI), Qingkailing injection (QKLI), Reduning injection (RDNI), Yanhuning injection (YHNI), and Tanreqing injection (TRQI). QKLI was most effective in alleviating symptoms of fever and improving overall clinical effectiveness. TRQI was most effective in relieving cough symptoms. YHNI was most effective in alleviating sore throat, runny nose, and nasal congestion. The overall incidence of adverse effects of these herbal injections in the treatment of AURI was lower, and their safety profiles were better.

**Conclusions:**

The herbal injections combined with ribavirin improved clinical outcomes, and were superior to ribavirin injection alone in alleviating clinical symptoms such as fever, cough, sore throat, runny nose, and nasal congestion, and have favorable safety profiles.

**Systematic Review Registration:**

https://www.crd.york.ac.uk/prospero/display_record.php?ID=CRD42023484099, CRD42023484099.

## 1 Introduction

Acute upper respiratory tract infection (AURI) is a general term for acute inflammation of the nasal, pharyngeal, and laryngeal regions that includes a group of diseases, predominantly acute nasopharyngolaryngitis, as well as viral pharyngitis, laryngitis, herpetic pharyngitis, pharyngoconjunctival fever, and tonsillitis (1). It is a common respiratory disease in pediatric outpatient clinics and is mainly characterized by rapid progression and severe fever. Because of children's young age and immaturity, they are susceptible to infections induced by viral and bacterial invasion. If AURI is not controlled in time, it may easily develop into inflammatory lesions in adjacent organs, lymph nodes, etc., and in severe cases inflammation of the lungs may also occur (2, 3). Owing to the unclear symptoms of children's complaints and the rapid progression of the disease, the complication rate is high, and, if not diagnosed early and given symptomatic treatment, delays are easily caused, which adversely affects the physical and mental health of the children (4, 5). The etiology of AURI mostly comprises the invasion of the nasopharynx by viruses such as influenza virus and adenovirus, and the clinical symptoms include headache, fever, sore throat, and runny nose (6). Owing to differences in individual immunity, the disease varies in severity, and, although it is a self-limiting disease with a good prognosis, it may cause complications and further affect health if not treated in time (7). Treatment of this disease in Western medicine is based on antipyretic drugs and antibiotics, and ribavirin is often used in clinical treatment, but its effect is not good.

Traditional Chinese medicine (TCM) is characterized by holistic interventions based on multiple targets and multiple pathways in preventing respiratory diseases in children and improving patients’ prognosis (8). Traditional Chinese medicine injections (TCMIs) are patented traditional Chinese drugs registered by the National Medical Products Administration. Chinese medicine injections are injections prepared under the guidance of Chinese medicinal theory using modern pharmaceutical technology to purify and concentrate the active ingredients in single and compound Chinese medicinal formulations. In recent years, clinical evidence has accumulated for the treatment of AURI with Chinese herbal injections in combination with Western drugs, which can effectively relieve symptoms and improve levels of serum-related factors and blood rheology indices (9–11). However, owing to the large differences in efficacy and the great variety of such drugs, as well as the shortage of studies and evaluations of direct comparisons among TCMIs, there is still a great deal of difficulty in the selection of the optimal treatment regimen in clinical work. Network meta-analysis (NMA) enables quantitative evaluation and ranking of multiple interventions for the same disease on the basis of direct and indirect comparisons (12).

The specific efficacies and therapeutic advantages of TCMIs are unclear, which causes problems in clinical application. This study is the first article to systematically evaluate and compare the clinical efficacies and safety of several commonly used TCMIs in combination with ribavirin. The aim of this study is to provide sufficient evidence-based medical evidence and to inform the use of TCMIs for the treatment of AURI in the clinic.

## 2 Materials and methods

### 2.1 Standard evaluation of traditional Chinese medicines

To make the study more accurate and reproducible, this study refers to the ConPhyMP consensus (13). In addition, we standardized the naming of herbal medicines (14) and validated the names against the Plants of the World Online (http://www.plantsoftheworldonline.org) and the World Flora Online (http://www.worldfloraonline.org/) databases. Summary tables describing the compositions of agents and how they were reported in the original studies were prepared in accordance with the principles described in the four pillars of ethnopharmacology. The composition and standard name of each injection are shown in Table 1.

**Table 1:** Compositions of the traditional Chinese medicine injections.

Drug name	Scientific plant or animal name/medicinal component	Family	Plant parts used
Xiyanping injection	*Andrographis paniculata*	Acanthaceae	Roots
Qingkailing injection	*Isatis tinctoria*	Brassicaceae	Roots
	*Bubalus bubalis* (Cornu Bubali)	—	—
	Baicalin	—	—
	*Lonicera japonica*	Caprifoliaceae	Flower buds
	*Gardenia jasminoides* (Fructus Gardeniae)	Rubiaceae	Fruits
	Pteriidae	Pteriidae	—
	Cholic acid	Suidae	—
Reduning injection	*Artemisia carvifolia*	Compositae	Roots
	*Gardenia jasminoides* (Fructus Gardeniae)	Rubiaceae	Fruits
	*Lonicera japonica*	Caprifoliaceae	Flower buds
Yanhuning injection	*Andrographis paniculata*	Acanthaceae	Roots
Tanreqing injection	*Scutellaria baicalensis*	Labiatae	Roots
	Fel Ursi	Ursidae	—
	Cornu Naemorhedi	Caprinae	—
	*Lonicera japonica*	Caprifoliaceae	Flower buds
	*Forsythia suspensa*	Oleaceae	Flower buds

### 2.2 Systematic review protocol and registration

The NMA was registered with the international prospective register of systematic reviews (PROSPERO) under the registration number CRD42023484099. We followed the Preferred Reporting Items for Systematic Reviews and Meta-Analyses (PRISMA) guidelines, the associated protocols, and the PRISMA extension statement for network meta-analyses to report the current results (15, 16).

### 2.3 Literature search

This study searched a total of eight databases, namely, PubMed, Embase, the Cochrane Library, Web of Science, SinoMed, China National Knowledge Infrastructure (CNKI), the Wanfang database, and the Chinese Scientific Journal database (VIP). The main search terms included “Traditional Chinese medicine injections*,” “Respiratory Tract Infections,” “Infection, Respiratory Tract,” “Respiratory Tract Infection,” “Infections, Respiratory,” “Infections, Respiratory Tract,” “Infections, Upper Respiratory,” and “Respiratory Infection, Upper.” References from previous systematic reviews and meta-analyses with similar topics were scanned for supplementation in the preliminary screening stage. References from eligible articles were scanned for supplementation in the full-text screening stage, and unpublished studies were not retrieved. The detailed search strategy is presented in Supplementary Tables S1–S8 (pages 1–S5). The search was limited to the period from 1 January 2013 to 1 November 2023.

### 2.4 Inclusion and exclusion criteria

Inclusion criteria were devised according to the patient, intervention, comparator, and outcome (PICO) framework: (a) the type of study included comprised randomized controlled trials (RCTs); (b) the type of disease studied was a viral infection of the upper respiratory tract (including viral pharyngitis, laryngitis, herpetic pharyngitis, pharyngoconjunctival fever, and tonsillitis) rather than a bacterial infection (no limitations applied in terms of age, sex, or nationality); (c) in the treatment group, the intervention was TCMIs; and (d) the primary outcome in the study was the total effectiveness rate. The secondary outcomes included the times to resolution of fever, cough, sore throat, runny nose, and nasal congestion.

The following exclusion criteria were used: (a) duplicated articles; (b) incomplete or incorrect data; and (c) nonconforming studies (including reviews, systematic reviews, meta-analyses, animal experiments, conference abstracts, reports, letters, and case reports).

### 2.5 Study selection and data extraction

Two researchers (XYG and CXL) from related disciplines independently screened and crosschecked studies for inclusion. In the case of disagreement, a third researcher (QZ or SJYH) adjudicated and provided a solution. Preliminary screening was carried out according to the title and abstract, and the included studies were then selected by reading the full text. Two researchers used uniform criteria for data extraction: the first author, year of publication, duration of AURI, sample size, male-to-female ratio, age, interventions, course of treatment, and outcomes.

### 2.6 Risk of bias assessment and quality assessment

The quality of the included studies was assessed by two investigators (XYG and CXL) using the Cochrane Risk of Bias 2.0 tool (17), which includes the randomization process, deviations from the intended interventions, missing outcome data, measurement of outcomes, selection of the reported results, and overall bias. The risk of bias was classified as “low risk,” “high risk,” or “some concerns.” We used the Grading of Recommendations, Assessment, Development, and Evaluations (GRADE) method for the entire network to provide a framework for the deterministic rating of each paired comparison, which was classified as high, medium, low, or very low (18, 19).

### 2.7 Statistical analysis

The NMA was performed using Stata 15.0 for NMA version on the basis of the frequentist framework. Results between pairwise comparisons were reported using the netleague command and were presented in tabular form. For dichotomous variables, the odds ratio (OR) was used as the effect analysis statistic; for continuous variables, the mean difference (MD) was used as the effect analysis statistic. For continuous variables, differences were not statistically significant when the 95% CI included 0. For dichotomous variables, the difference was not statistically significant when the 95% CI included 1.Evidence network diagrams were used to show direct comparisons between different interventions, where the size of each node represented the sample size for the corresponding intervention and the thickness of the line connecting two nodes indicated the number of studies that directly compared the two interventions. The surface area under the cumulative ranking curve (SUCRA) was calculated for each intervention, where SUCRA was expressed as a value in the range of 0%–100% and represented the probability that the treatment was the best choice. A “comparison–correction” funnel plot was drawn to assess the publication bias of the included studies. If a closed loop was formed, an inconsistency test was performed (20).

## 3 Results

### 3.1 Study selection and study characteristics

**Figure 1:**
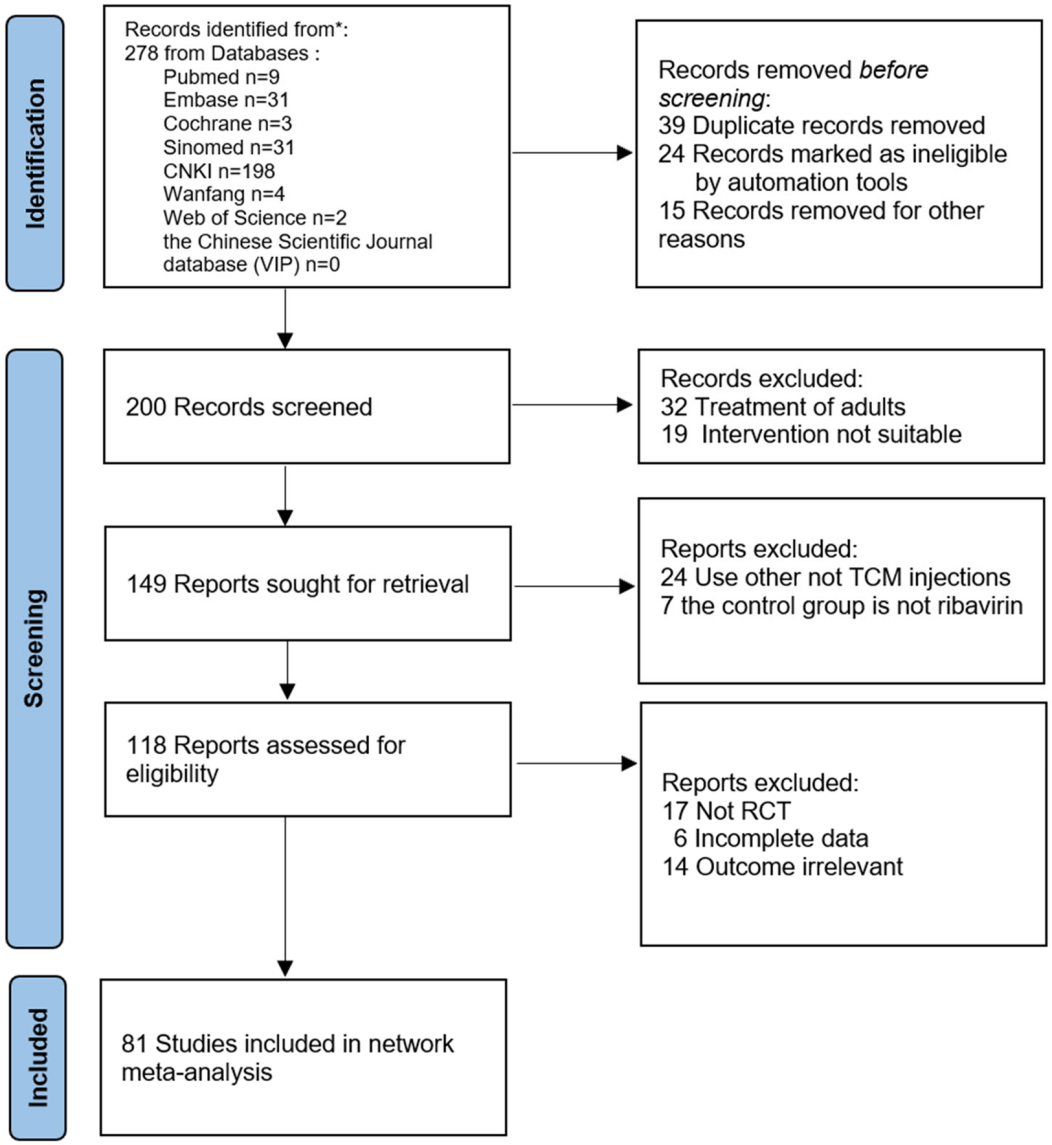
Flow diagram of study selection.

The initial review yielded 2023 relevant papers, and 81 RCTs, which were all two-arm studies, were finally included after the initial screening and rescreening (21–101). These included 11,737 patients: 5,904 in the treatment groups and 5,833 in the control groups. The 81 RCTs included in this study covered a total of six interventions, including five herbal injections, namely, Xiyanping injection (XYPI; 30 RCTs) (21–50); Qingkailing injection (QKLI; 2 RCTs) (51, 52); Reduning injection (RDNI; 22 RCTs) (53–74); Yanhuning injection (YHNI; 22 RCTs) (75–96); and Tanreqing injection (TRQI; 5 RCTs) (97–101). The specific search and screening process is described in Figure 1, and the basic characteristics of the included studies are shown in Table 2.

**Table 2:** Summary of all included studies.

No.	Study	Course of disease		Sample size		Sex (M/F)		Age (mean or range)		Interventions			Outcome(s)
		T	C	T	C	T	C	T	C	T	C		
1	Zhaohongxia (2015)	2.3 ± 0.3 days	2.1 ± 0.2 days	55	55	29/26	27/28	4.4 ± 0.6	4.5 ± 0.7	XYPI (5 mg/kg/day)	R (10 mg/kg/day)	3 days	①②③④
2	Lixinhua (2013)	NA		50	50	65/35		8.0 ± 2.6		XYPI (200 mg/day) + R (500 mg/day)	R (500 mg/day)	5 days	①②③⑤⑥⑦
3	Zhengjianxin (2013)	12.1 ± 2.5 h		68	68	78/58		2.3 ± 1.9		XYPI (5 mg/kg/day)	R (10 mg/kg/day)	7 days	②③④⑦
4	Mali (2013)	1–3 days		50	50	56/44		6.39 ± 2.14		XYPI (5 mg/kg/day)	R (10 mg/kg/day)	5 days	①②③④⑦
5	Zhaoruijing (2013)	NA	125	125	68/57	67/58	4.0 ± 1.3	4.2 ± 1.4	XYPI (5 mg/kg/day)	R (10 mg/kg/day)		7 days	①→②③④
6	Huangwenjing (2014)	3–5 days		60	60	64/56		NA		XYPI (5 mg/kg/day)	R (10 mg/kg/day)	5 days	①→②④⑦
7	Zhaoyajuan (2014)	1.8 ± 0.8 days		60	60	70/50		4.5 ± 1.5		XYPI (0.2–0.4 ml/kg/day)	R (10 mg/kg/day)	3 days	①→②
8	Zhangzhouhui (2013)	3.1 ± 0.7 days	3.3 ± 0.6 days	50	50	23/27	22/28	6.1 ± 1.1	5.5 ± 1.4	XYPI (6 mg/kg/day)	R (9 mg/kg/day)	7 days	①②③④⑦
9	Jiangfei (2014)	5–48 h		48	48	54/42		2.6 ± 1.3		XYPI (5 mg/kg/day)	R (10 mg/kg/day)	5 days	①②③④⑥
10	Maolinlin (2021)	8.3 ± 2.1 days	8.1 ± 2.1 days	35	35	22/13	21/14	7.2 ± 2.3	7.2 ± 2.2	XYPI (6 mg/kg/day)	R (9 mg/kg/day)	5 days	①②③
11	Dinglihong (2014)	NA		25	25	32/18		3–10		XYPI (0.2–0.4 ml/kg/day)	R (10 mg/kg/day)	7 days	①→
12	Panjie (2017)	12.9 ± 2.7 h	13.2 ± 2.5 h	49	49	26/23	28/21	2.2 ± 1.2	2.4 ± 1.0	XYPI (0.2–0.4 ml/kg/day)	R (10 mg/kg/day)	3 days	①→②③④
13	Liuji (2016)	2–13 h		49	49	54/44		0.5–4		XYPI (5–10 mg/kg/day)	R (10 mg/kg/day)	5 days	①②③④
14	Liujiarong (2016)	NA	30	30	17/13	19/11	2.6 ± 1.4	2.8 ± 1.2		XYPI (5 mg/kg/day)	R (10 mg/kg/day)	5 days	①→⑦
15	Moguipei (2016)	NA	40	40	24/16	27/13	4.2 ± 1.6	4.4 ± 1.7		XYPI (0.2–0.4 ml/kg/day)	R (10 mg/kg/day)	7 days	①②③④⑦
16	Wulin (2017)	1.9 ± 0.5 days		46	46	49/43		4.5 ± 1.7		XYPI (5 mg/kg/day)	R (10 mg/kg/day)	7 days	①②③④
17	Chenhaijun (2015)	NA		50	50	54/46		4.5 ± 0.8		XYPI (0.2–0.4 ml/kg/day)	R (10 mg/kg/day)	7 days	①→
18	Shenguijun (2017)	12.6 ± 3.2 h	12.2 ± 3.5 h	48	48	25/23	26/22	2.4 ± 1.1	2.5 ± 1.3	XYPI (5 mg/kg/day)	R (10 mg/kg/day)	7 days	①②③④⑤
19	Qinyan (2016)	NA		42	42	22/20	21/21	5.8 ± 1.3	5.5 ± 1.2	XYPI (5 mg/kg/day)	R (10 mg/kg/day)	5 days	①②③④
20	Wangyan (2015)	NA		100	96	38/62	43/53	5.1 ± 0.4	4.7 ± 0.6	XYPI (0.2–0.4 ml/kg/day)	R (10 mg/kg/day)	7 days	①②③④
21	Zhulili (2016)	2.5 days	2.0 days	98	98	59/39	56/42	2.4 ± 1.5	3.3 ± 1.5	XYPI (5–10 mg/kg/day)	R (10 mg/kg/day)	7 days	①②③
22	Liuguifang (2015)	NA		50	50	32/18	31/19	7.2 ± 2.1	7.5 ± 2.3	XYPI (5–10 mg/kg/day)	R (10 mg/kg/day)	5 days	①→
23	Gongtianyin (2016)	NA		99	91	53/46	51/40	3.2	3.8	XYPI (5 mg/kg/day)	R (10 mg/kg/day)	5 days	①→
24	Ganxiaohong (2014)	NA		60	60	29/31	28/32	3.5 ± 0.9	3.7 ± 0.8	XYPI (0.2–0.4 ml/kg/day)	R (10 mg/kg/day)	5 days	①②③④
25	Hanchangming (2016)	5.6 ± 1.2 days	5.8 ± 1.2 days	50	50	25/25	26/24	5.6 ± 1.4	5.8 ± 1.4	XYPI (0.2–0.4 ml/kg/day)	R (10 mg/kg/day)	5 days	①→
26	Zhangyuqin (2018)	NA		40	40	49/31		4.3 ± 2.1		XYPI (200 mg/day)	R (500 mg/day)	5 days	①→②③④
27	Yanghongying (2015)	NA		41	41	22/19	23/18	5.3 ± 1.1	5.6 ± 1.3	XYPI (5 mg/kg/day)	R (10 mg/kg/day)	5 days	①②③④⑦
28	Zhangjun (2015)	NA		44	44	24/20	20/24	3.5	3.6	XYPI (5 mg/kg/day)	R (10 mg/kg/day)	5 days	②→
29	Zhumantang (2017)	1.7 ± 0.6 days	1.6 ± 0.5 days	60	60	39/21	37/23	5.8 ± 0.9	5.7 ± 0.8	XYPI (5 mg/kg/day)	R (10 mg/kgay)ay	5 days	①→⑦
30	Yijingting (2016)	3.0 ± 1.2 days	3.0 ± 1.5 days	60	60	39/21	33/27	4.0 ± 1.1	3.0 ± 1.6	XYPI (5–10 mg/kg/day)	R (10 mg/kg/day)	7 days	①②③④⑦
31	Guoyewei (2014)	1.8 ± 0.4 days	1.6 ± 0.6 days	50	50	27/23	29/21	1.0 ± 0.6	1.0 ± 0.7	QKLI (0.8–1 ml/kg/day)	R (10 mg/kg/day)	5 days	①→
32	Panhong (2015)	NA		100	100	52/48	50/50	5.6 ± 2.3	5.9 ± 2.5	QKLI (0.5–0.6 ml/kg/day)	R (10 mg/kg/day)	7 days	①②③⑦
33	Liuqin (2015)	NA		61	61	30/31	29/32	5.4 ± 1.5	5.5 ± 1.3	RDNI (0.6 ml/kg/day)	R (10 mg/kg/day)	3 days	①②③④⑥
34	Zhangxian (2013)	NA		68	61	NA		1–6		RDNI (0.5–0.8 ml/kg/day)	R (10 mg/kg/day)	3 days	①②③④⑥
35	Dingpei (2013)	NA		58	58	61/55		1–12		RDNI (0.3–0.5 ml/kg/day)	R (10 mg/kg/day)	5 days	①②③④
36	Liuhui (2015)	24–72 h		62	61	65/58		10 months–11 years		RDNI (0.3–0.5 ml/kg/day)	R (10 mg/kg/day)	5 days	①→
37	Zhouwenwen (2014)	<48 h		42	42	20/22	19/21	NA		RDNI (0.3–0.5 ml/kg/day)	R (10 mg/kg/day)	3 days	①→
38	Cuibeiyong (2013)	NA		120	110	62/58	53/57	3 months–3 years		RDNI (0.5–0.8 ml/kg/day)	R (10 mg/kg/day)	5 days	①③⑥
39	Liuhaiqin (2013)	2.7 ± 0.5 days	2.5 ± 0.6 days	38	38	21/17	23/15	4.4 ± 0.5	4.2 ± 0.6	RDNI (0.5 ml/kg/day)	R (10 mg/kg/day)	7 days	①→②
40	Huchunxia (2014)	NA		150	150	75/75	75/75	6 months–6 years		RDNI (0.5 ml/kg/day)	R (10 mg/kg/day)	7 days	①②③
41	Liumingqi (2014)	NA		104	104	118/90		<18 years		RDNI (0.5–1 ml/kg/day)	R (10 mg/kg/day)	5 days	①②④⑤
42	Limingchun (2014)	<24 h		84	84	44/40	45/39	11 months–5 years	10 months–5 years	RDNI (0.5 ml/kg/day)	R (10 mg/kg/day)	4 days	①②③④⑦
43	Guyuxing (2013)	NA		120	120	80/40	78/42	7.2 ± 1.2	7.1 ± 1.6	RDNI (0.5–0.8 ml/kg/day)	R (10 mg/kg/day)	5 days	①②⑦
44	Muchunjie (2013)	NA		68	68	38/30	36/32	5.2 ± 1.4	5.3 ± 1.4	RDNI (0.6 ml/kg/day)	R (10 mg/kg/day)	3 days	①→
45	Zhaoqun (2018)	2.2 ± 0.3 days	2.3 ± 0.1 days	45	45	28/17	27/18	6.2 ± 2.6	6.3 ± 2.5	RDNI (0.6 ml/kg/day)	R (10 mg/kg/day)	4 days	①②③④⑥⑦
46	Xudongsheng (2015)	NA		90	90	54/36	46/44	7.3 ± 1.2	7.4 ± 1.1	RDNI (0.6 ml/kg/day)	R (10 mg/kg/day)	4 days	①②③④⑤⑦
47	Zhuangtao (2015)	2.2 ± 0.3 days	2.2 ± 0.4 days	39	39	21/18	20/19	5.1 ± 1.2	5.2 ± 1.2	RDNI (0.5 ml/kg/day)	R (10 mg/kg/day)	3 days	①②③④⑤⑦
48	Fangwanfen (2015)	1.7 ± 0.8 days	1.6 ± 0.8 days	59	59	32/27	30/29	6.0 ± 1.6	5.8 ± 1.7	RDNI (0.6 ml/kg/day)	R (10 mg/kg/day)	3 days	①②③④⑥⑦
49	Lihao (2017)	50.4 ± 10.6 h	50.5 ± 10.2 h	34	34	18/16	19/15	8.5 ± 2.4	8.3 ± 2.7	RDNI (0.6 ml/kg/day)	R (10 mg/kg/day)	3 days	①②③④⑥⑦
50	Zhangzhigang (2019)	NA		43	43	25/18	26/17	4.9 ± 4.0	5.4 ± 4.6	RDNI (0.6 ml/kg/day)	R (10 mg/kg/day)	3 days	①②③④⑥
51	Zhangjie (2015)	NA		60	60	32/28	31/29	6.4 ± 1.2	6.5 ± 1.0	RDNI (0.6 ml/kg/day)	R (10 mg/kg/day)	3 days	①②③④⑥⑦
52	Liuyan (2015)	2.1 ± 0.9 days	2.4 ± 0.8 days	45	45	25/20	24/21	3.3 ± 2.2	3.1 ± 2.3	RDNI (0.5–0.8 ml/kg/day)	R (10 mg/kg/day)	3 days	①→
53	Liubinxiu (2015)	<48 h		40	40	20/20	22/18	3.4 ± 1.7	3.4 ± 1.9	RDNI (10 ml/day)	R (10 mg/kg/day)	3 days	①②③④⑥
54	Liuzhongyan (2015)	NA		104	102	NA		NA		RDNI (0.5–0.8 ml/kg/day)	R (10 mg/kg/day)	3 days	①→②⑦
55	Weiyueru (2014)	1.8 ± 0.6 days		102	101	106/97		3.2 ± 1.6		YHNI (5–10 mg/kg/day)	R (10 mg/kg/day)	3 days	①②③④⑥⑦
56	Wangguiying (2014)	NA		50	50	29/31	25/25	NA		YHNI (5–10 mg/kg/day)	R (10 mg/kg/day)	3 days	①→②
57	Wuguobin (2013)	4 days		25	25	28/22		4.8 ± 1.2		YHNI (5–10 mg/kg/day)	R (10 mg/kg/day)	3 days	①②③⑥
58	Saiqin (2015)	NA		40	40	23/17	19/21	3.8 ± 1.6	3.7 ± 1.5	YHNI (10 mg/kg/day)	R (10 mg/kg/day)	3 days	①→
59	Mushuyun (2013)	4 days–2 months		50	50	56/44		7 months–9 years		YHNI (5–10 mg/kg/day)	R (10 mg/kg/day)	3 days	① ②③⑥⑦
60	Wangxinlian (2019)	2.3 ± 0.7 days	2.5 ± 1.1 days	95	95	52/43	45/50	5.3 ± 1.2	5.6 ± 1.1	YHNI (0.16–0.4 g/day)	R (10 mg/kg/day)	3 days	①②③④⑤⑦
61	Miuchunjie (2013)	NA		60	60	34/26	33/27	5.2 ± 1.4	5.3 ± 1.4	YHNI (40–80 mg/day)	R (10 mg/kg/day)	3 days	①→
62	Tangliqun (2013)	4.2 ± 0.5 days	4.0 ± 0.7 days	105	105	61/44	65/40	4.5 ± 0.6	4.7 ± 0.8	YHNI (5–10 mg/kg/day)	R (10 mg/kg/day)	3 days	①②③④⑥
63	Kuangchaobo (2014)	20.56 ± 2.33 h		60	60	64/56		4.56 ± 0.33		YHNI (240 mg/day)	R (10 mg/kg/day)	3 days	①→
64	Tanghongjun (2015)	NA		82	44	48/34	23/21	48.9 ± 2.8 months	48.5 ± 2.6 months	YHNI (5–10 mg/kg/day)	R (10 mg/kg/day)	7 days	①→
65	Jifengying (2014)	NA		103	103	54/49	53/50	3.0 ± 1.5	3.1 ± 1.3	YHNI (10 mg/kg/day)	R (10 mg/kg/day)	3 days	①②③④⑥
66	Liuaipeng (2014)	3.9 ± 0.2 days	3.9 ± 0.2 days	141	141	72/69	73/68	4.1 ± 0.7	4.2 ± 0.8	YHNI (10 mg/kg/day)	R (10 mg/kg/day)	3 days	①②③④⑥⑦
67	Zhangjunjing (2017)	3.5 ± 1.3 days	3.4 ± 1.2 days	40	40	24/16	22/18	5.6 ± 1.1	5.8 ± 1.0	YHNI (5–10 mg/kg/day)	R (10 mg/kg/day)	3 days	①→②
68	Baoliang (2016)	6–48 h		58	58	76/40		1–6		YHNI (0.16 g/day)	R (10 mg/kg/day)	7 days	②③④⑥⑦
69	Zhaona (2015)	NA		40	40	22/18	21/19	NA		YHNI (5 mg/kg/day)	R (10 mg/kg/day)	7 days	①→
70	Lvhuanming (2019)	3.2 ± 1.0 days	3.1 ± 1.1 days	30	30	18/12	17/13	5.2 ± 1.3	5.1 ± 1.3	YHNI (5–10 mg/kg/day)	R (10 mg/kg/day)	5 days	①②③④⑦
71	Zhuyuemei (2017)	1.8 ± 0.3 days	1.7 ± 0.4 days	45	45	25/20	26/19	5.2 ± 1.2	5.2 ± 1.2	YHNI (160–400 mg/day)	R (10 mg/kg/day)	3 days	①②③④⑥
72	Lixiaoli (2018)	NA		39	39	21/18	20/19	2–6		YHNI (5–10 mg/kg/day)	R (10 mg/kg/day)	3 days	①→②
73	Yanghonggui (2015)	3.5 ± 0.5 days	4.0 ± 0.7 days	86	86	51/35	49/37	3.5 ± 0.6	3.7 ± 0.8	YHNI (5–10 mg/kg/day)	R (10 mg/kg/day)	3 days	①②③④⑥
74	Lijiahong (2015)	NA		30	30	16/14	15/15	5.3 ± 4.2	4.8 ± 3.6	YHNI (5–8 mg/kg/day)	R (10 mg/kg/day)	3 days	①②③
75	Haobingfeng (2017)	<3 days		1,000	1,000	523/477	520/480	4.2 ± 2.1	4.3 ± 2.0	YHNI (10 mg/kg/day)	R (10 mg/kg/day)	3 days	①②③④⑥⑦
76	Yanghaizhen (2023)	3.4 ± 1.1 days	3.1 ± 1.2 days	35	35	19/16	20/15	3.3 ± 1.2	3.2 ± 1.1	YHNI (160–400 mg/day)	R (10 mg/kg/day)	5 days	①②③④
77	Cuibingzhong (2013)	2.4 ± 1.2 days	2.6 ± 1.3 days	40	40	21/19	18/22	6.6 ± 2.2	6.8 ± 2.1	TRQI (0.3–0.5 ml/kg/day)	R (10 mg/kg/day)	5 days	①→⑦
78	Wangchunying (2014)	NA		52	52	56/48		6.5 ± 2.4		TRQI (0.3–0.5 ml/kg/day)	R (10 mg/kg/day)	7 days	①②③⑤
79	Wanmin (2016)	NA		50	50	55/45		2–5.2		TRQI (0.3–0.5 ml/kg/day)	R (5 mg/kg/day)	7 days	①→
80	Kongshanshan (2018)	2.0 ± 0.6 days	2.2 ± 0.4 days	40	40	22/18	20/20	5.3 ± 1.2	5.8 ± 1.3	TRQI (0.3–0.5 ml/kg/day)	R (10 mg/kg/day)	5 days	①→③④⑤
81	Guohui (2017)	NA	NA	40	40	19/21	21/19	5.0 ± 0.5	4.2 ± 0.5	TRQI (0.8 ml/kg/day)	R (8 mg/kg/day)	5 days	①⑦

T, treatment group; C, control group; XYPI, Xiyanping injection; QKLI, Qingkailing injection; RDNI, Reduning injection; YHNI, Yanhuning injection; TRQI, Tanreqing injection; R, Ribavirin injection. Ages are in years unless otherwise stated.

① Total effectiveness rate; ② Time to relief of fever; ③ Time to relief of cough; ④ Time to relief of sore throat; ⑤ Time to relief of runny nose; ⑥ Time to relief of nasal congestion; ⑦ Adverse reactions.

### 3.2 Risk of bias results

**Figure 2:**
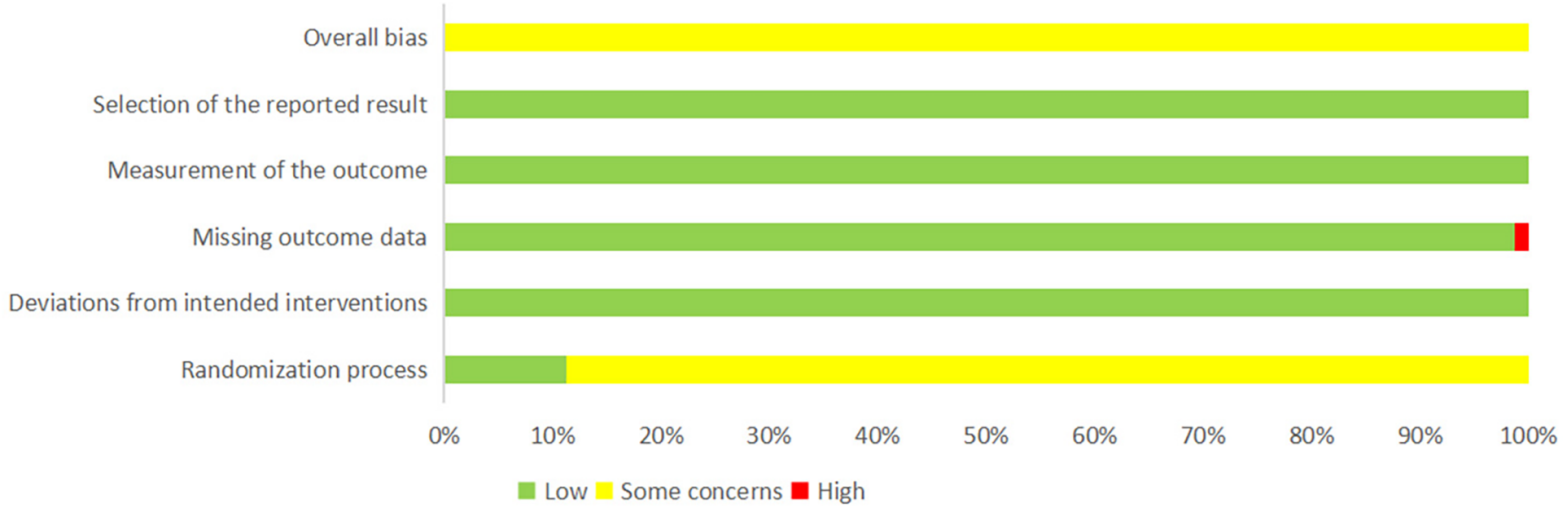
Percentages of reporting items of included articles that produced risks of bias.

The quality of the included studies was assessed using the risk assessment tool recommended by the Cochrane Collaboration. Among the 81 included studies, nine studies (55, 58, 63, 65, 67, 80, 85, 90, 100) mentioned the random number table method and were rated as “low risk.” The remaining 72 studies only mentioned randomization and were rated as “some concerns.” All studies were tested according to the established allocation to interventions, and those with no deviations were rated as “low risk.” One study (31) recorded outcome data with omissions and was rated as “high risk,” whereas the others reported complete outcome data with no exclusions or omissions and were rated as “low risk.” All the studies used appropriate measures for the outcome data and had no reporting bias in terms of outcome selection and were rated as “low risk.” The results of the risk of bias assessment of the included studies are shown in Figure 2 and Supplementary Table S10 (pages 11–S74).

### 3.3 Network diagrams

**Figure 3:**
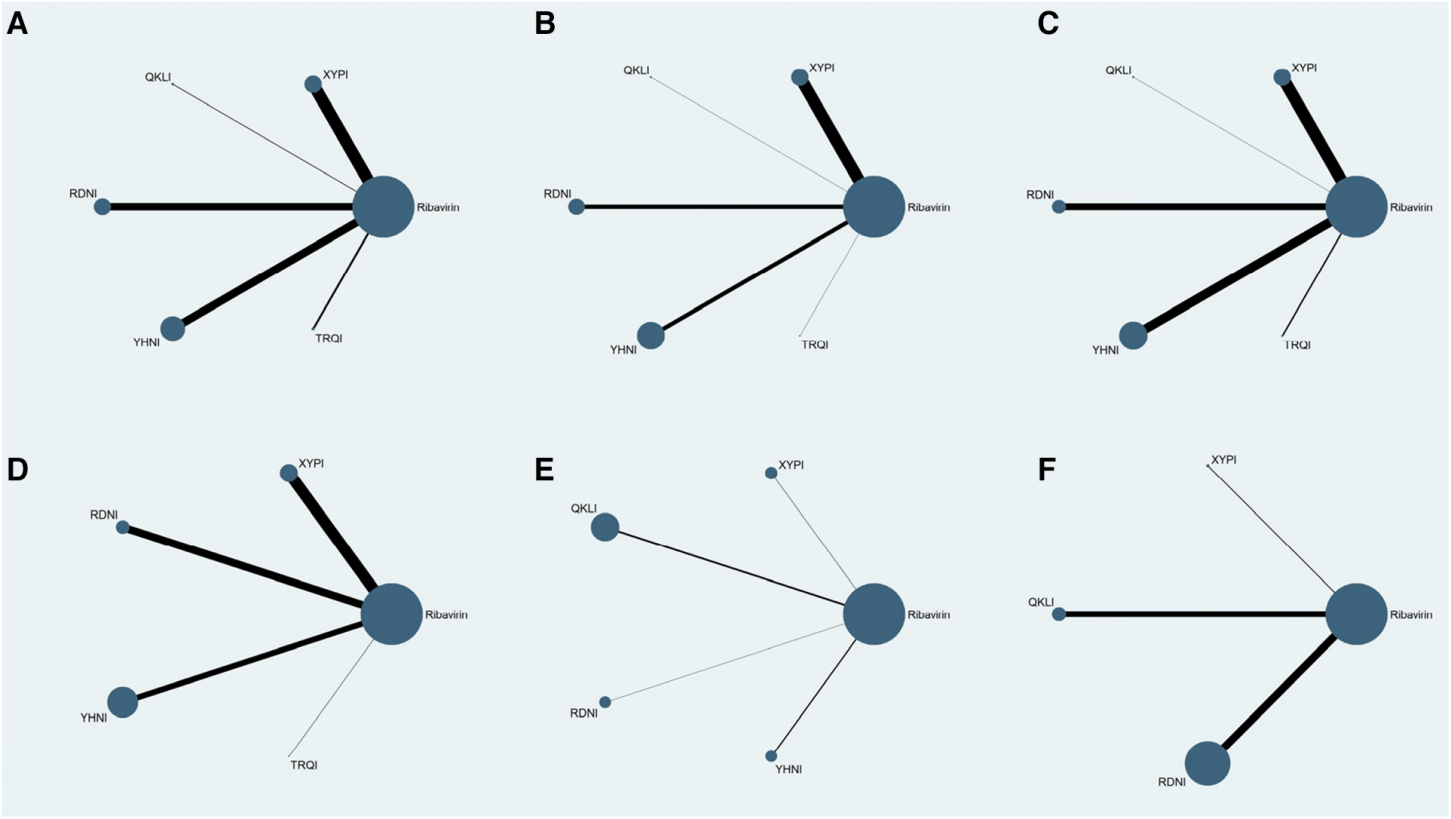
Evidence networks for the outcome indicators. (**A**) Clinical effectiveness; (**B**) time to relief of fever; (**C**) time to relief of cough; (**D**) time to relief of sore throat; (**E**) time to relief of runny nose; (**F**) time to relief of nasal congestion.

Seventy-eight RCTs reported clinical effectiveness and involved five TCMIs and six interventions; 58 RCTs reported time taken to reduce fever and involved five TCMIs and six interventions; 51 RCTs reported time to relief of cough and involved five TCMIs and six interventions; 43 RCTs reported time to relief of sore throat and involved four TCMIs and five interventions; 8 RCTs reported time to relief of runny nose and involved four TCMIs and five interventions; and 21 RCTs reported time to relief of nasal congestion and involved three TCMIs and four interventions. In the evidence networks constructed for the different outcome indicators, the size of a node represents the corresponding study sample size, and the thickness of the line connecting two nodes represents the number of included studies. There was no closed loop between the different interventions, and therefore the consistency model was used for the analysis: see Figure 3.

### 3.4 Results of network meta-analysis

#### 3.4.1 Clinical effectiveness

**Figure 4:**
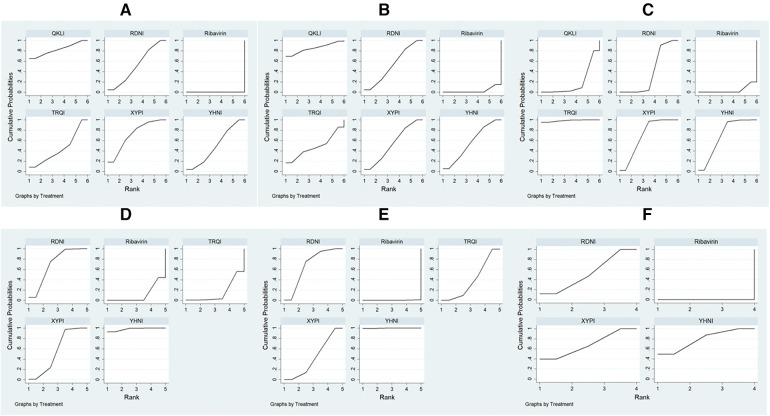
Plots of the surface area under the cumulative ranking curve. (**A**) Total effectiveness rate; (**B**) time to relief of fever; (**C**) time to relief of cough; (**D**) time to relief of sore throat; (**E**) time to relief of runny nose; (**F**) time to relief of nasal congestion.

A total of 11,396 patients involved in 78 studies (21, 22, 24–47, 49–87, 89–101) were evaluated, and the total effectiveness rate of five TCMIs and six interventions was reported. All five TCMIs were better than ribavirin (*P* < 0.05), as follows: QKLI [relative risk (RR): 0.21, CI: 0.10–0.33], XYPI (RR: 0.18, CI: 0.15–0.21), RDNI (RR: 0.16, CI: 0.12–0.20), YHNI (RR: 0.16, CI: 0.12–0.20), and TRQI (RR: 0.15, CI: 0.07–0.23), which suggested that they had advantages in alleviating clinical symptoms (Table 3). In addition, none of the differences between groups were statistically significant in pairwise comparisons between the five TCMIs (*P* > 0.05). According to the results of the SUCRA ranking, QKLI (82.7%) was the best treatment, followed by XYPI (71.8%) and RDNI (51.9%) (Table 4; Figure 4).

**Table 3:** League table for all outcome measures.

Clinical effectiveness	QKLI	XYPI	RDNI	YHNI	TRQI	Ribavirin
QKLI	QKLI	—	—	—	—	—
XYPI	0.03 (−0.09, 0.15)	XYPI	—	—	—	—
RDNI	0.05 (−0.07, 0.17)	0.02 (−0.03, 0.06)	RDNI	—	—	—
YHNI	0.05 (−0.07, 0.17)	0.02 (−0.03, 0.07)	0.00 (−0.05, 0.05)	YHNI	—	—
TRQI	0.06 (−0.08, 0.20)	0.03 (−0.06, 0.12)	0.01 (−0.07, 0.10)	0.01 (−0.07, 0.10)	TRQI	—
Ribavirin	**0.21** (**0.10, 0.33)**	**0.18** (**0.15, 0.21)**	**0.16** (**0.13, 0.20)**	**0.16** (**0.12, 0.20)**	**0.15** (**0.07, 0.23)**	Ribavirin
Time to relief of fever	QKLI	YHNI	XYPI	RDNI	TRQI	Ribavirin
QKLI	QKLI	—	—	—	—	—
YHNI	−0.80 (−2.49, 0.90)	YHNI	—	—	—	—
XYPI	−0.82 (−2.51, 0.86)	−0.03 (−0.56, 0.51)	XYPI	—	—	—
RDNI	−0.82 (−2.51, 0.87)	−0.02 (−0.58, 0.54)	0.00 (−0.53, 0.53)	RDNI	—	—
TRQI	−0.97 (−3.29, 1.35)	−0.17 (−1.86, 1.51)	−0.15 (−1.83, 1.53)	−0.15 (−1.84, 1.54)	TRQI	—
Ribavirin	−**1.86** (−**3.51,** −**0.21)**	−**1.06** (−**1.46,** −**0.67)**	−**1.04** (−**1.39,** −**0.68)**	−**1.04** (−**1.44,** −**0.64)**	−0.89 (−2.53, 0.75)	Ribavirin
Time to relief of sore throat	YHNI	RDNI	XYPI	TRQI	QKLI	Ribavirin
YHNI	YHNI	—	—	—	—	—
RDNI	−0.34 (−0.77, 0.09)	RDNI	—	—	—	—
XYPI	−**0.49** (−**0.89,** −**0.08)**	−0.14 (−0.53, 0.24)	XYPI	—	—	—
TRQI	−**1.53** (−**2.65,** −**0.41)**	−**1.19** (−**2.30,** −**0.08)**	−1.05 (−2.15, 0.06)	TRQI	—	—
QKLI	—	—	—	—	QKLI	—
Ribavirin	−**1.63** (−**1.95,** −**1.32)**	−**1.29** (−**1.58,** −**1.00)**	−**1.15** (−**1.40,** −**0.89)**	−0.10 (−1.17, 0.97)	—	Ribavirin
Time to relief of cough	TRQI	XYPI	YHNI	RDNI	QKLI	Ribavirin
TRQI	TRQI	—	—	—	—	—
XYPI	−0.73 (−1.50, 0.04)	XYPI	—	—	—	—
YHNI	−0.73 (−1.52, 0.06)	0.00 (−0.35, 0.35)	YHNI	—	—	—
RDNI	−**1.11** (−**1.89,** −**0.33)**	−**0.38** (−**0.73,** −**0.03)**	−0.38 (−0.76, 0.00)	RDNI	—	—
QKLI	−**1.77** (−**2.97,** −**0.58)**	−**1.04** (−**2.01,** −**0.08)**	−**1.04** (−**2.03,** −**0.06)**	−0.66 (−1.64, 0.31)	QKLI	—
Ribavirin	−**2.19** (−**2.93,** −**1.46)**	−**1.46** (−**1.69,** −**1.24)**	−**1.46** (−**1.74,** −**1.19)**	−**1.08** (−**1.35,** −**0.82)**	−0.42 (−1.36, 0.52)	Ribavirin
Time to relief of runny nose	YHNI	RDNI	XYPI	TRQI	QKLI	Ribavirin
YHNI	YHNI	—	—	—	—	—
RDNI	−**1.09** (−**1.92,** −**0.26)**	RDNI	—	—	—	—
XYPI	−**1.42** (−**2.28,** −**0.56)**	−0.33 (−0.99, 0.32)	XYPI	—	—	—
TRQI	−**1.47** (−**2.35,** −**0.59)**	−0.38 (−1.06, 0.29)	−0.05 (−0.76, 0.66)	TRQI	—	—
QKLI	—	—	—	—	QKLI	—
Ribavirin	−**2.11** (−**2.82,** −**1.40)**	−**1.02** (−**1.45,** −**0.59)**	−**0.69** (−**1.18,** −**0.20)**	−**0.64** (−**1.16,** −**0.12)**	—	Ribavirin
Time to relief of nasal congestion	YHNI	XYPI	RDNI	TRQI	QKLI	Ribavirin
YHNI	YHNI	—	—	—	—	—
XYPI	−0.08 (−0.83, 0.68)	XYPI	—	—	—	—
RDNI	−0.19 (−0.66, 0.27)	−0.11 (−0.88, 0.65)	RDNI	—	—	—
TRQI	—	—	—	TRQI	—	—
QKLI	—	—	—	—	QKLI	—
Ribavirin	−**1.22** (−**1.54,** −**0.89)**	−**1.14** (−**1.82,** −**0.45)**	−**1.02** (−**1.36,** −**0.69)**	—	—	Ribavirin

XYPI, Xiyanping injection; QKLI, Qingkailing injection; RDNI, Reduning injection; YHNI, Yanhuning injection; TRQI, Tanreqing injection.

Significant effects are printed in bold.

**Table 4:** Results for the surface area under the cumulative ranking curve (SUCRA) (%).

	Ribavirin	XYPI	QKLI	RDNI	YHNI	TRQI
Clinical effectiveness	0.0	71.8	82.7	51.9	49.6	43.9
Time to relief of fever	3	53.9	85.3	53.4	56.2	48.2
Time to relief of cough	3.9	70.1	18.6	39.1	69.8	98.6
Time to relief of sore throat	11.0	55.5	—	70.1	98.1	15.3
Time to relief of runny nose	0.3	43.0	—	67.9	99.8	39.0
Time to relief of nasal congestion	0.0	68.3	—	52.8	78.9	—

The intervention in green was most likely to be the best intervention, whereas that in yellow was second and that in red was third. XYPI, Xiyanping injection; QKLI, Qingkailing injection; RDNI, Reduning injection; YHNI, Yanhuning injection; TRQI, Tanreqing injection.

#### 3.4.2 Time to relief of fever

A total of 9,268 patients involved in 58 studies (21–30, 32, 33, 35, 36, 38–41, 44, 46–48, 50, 52–55, 59–63, 65–71, 73–80, 82, 85–88, 90–96, 98) were evaluated, and the time to relief of fever in the case of five TCMIs and six interventions was reported. Four TCMIs were better than ribavirin (*P* < 0.05), namely, QKLI [standardized mean difference (SMD): −1.96, CI: −3.51 to −0.21], YHNI (SMD: −1.06, CI: −1.46 to −0.67), XYPI (SMD: −1.04, CI: −1.39 to −0.68), and RDNI (SMD: −1.04, CI: −1.44 to −0.64), in terms of time to relief of fever (Table 3). In addition, none of the differences between groups were statistically significant in pairwise comparisons between the four TCMIs (*P* > 0.05). According to the results of the SUCRA ranking, QKLI (85.3%) was the best treatment, followed by YHNI (56.2%) and XYPI (53.9%) (Table 4; Figure 4).

#### 3.4.3 Time to relief of cough

A total of 8,382 patients involved in 51 studies (21–25, 27–30, 32, 33, 35, 36, 38–41, 44, 46, 47, 50, 52–55, 58, 60, 62, 65–71, 73, 75, 77, 79, 80, 82, 85, 86, 88, 90, 91, 93–96, 98, 100) were evaluated, and the time to relief of cough in the case of five TCMIs and six interventions was reported. Four TCMIs were better than ribavirin (*P* < 0.05), namely, XYPI (SMD: −1.46, CI: −1.69 to −1.23), RDNI (SMD: −1.08, CI: −1.35 to −0.82), YHNI (SMD: −1.46, CI: −1.74 to −1.19), and TRQI (SMD: −2.19, CI: −2.93 to −1.46), in terms of the time to relief of cough. Regarding the TCMIs, TRQI was better than RDNI and QKLI (SMD: −1.11, CI: −1.89 to −0.33/SMD: −1.77, CI: −2.97 to −0.58), XYPI was better than RDNI and QKLI (SMD: −0.38, CI: −0.73 to −0.03/SMD: −1.04, CI: −2.01 to −0.88), and YHNI was better than QKLI (SMD: −1.04, CI: −2.03 to −0.06), which suggested that they had advantages in the relief of cough (Table 3). According to the results of the SUCRA ranking, TRQI (98.6%) was the best treatment, followed by XYPI (70.1%) and YHNI (69.8%) (Table 4; Figure 4).

#### 3.4.4 Time to relief of sore throat

A total of 7,300 patients involved in 43 studies (21, 23–26, 28, 29, 32, 33, 35, 36, 38–40, 44, 46, 47, 50, 53–55, 61, 62, 65–71, 73, 75, 80, 82, 85, 86, 88, 90, 91, 93, 95, 96, 100) were evaluated, and the time to relief of sore throat in the case of four TCMIs and five interventions was reported. Three TCMIs were better than ribavirin (*P* < 0.05), namely, XYPI (SMD: −1.15, CI: −1.40 to −0.89), RDNI (SMD: −1.29, CI: −1.58 to −1.00), and YHNI (SMD: −1.63, CI: −1.95 to −1.32), in terms of the time to relief of sore throat. Regarding the TCMIs, YHNI was better than XYPI and TRQI (SMD: −0.49, CI: −0.89 to −0.08/SMD: −1.53, CI: −2.65 to −0.41), and RDNI was better than TRQI (SMD: −1.19, CI: −2.30 to −0.08), which suggested that they had advantages in the relief of sore throat (Table 3). According to the results of the SUCRA ranking, YHNI (98.1%) was the best treatment, followed by RDNI (70.1%) and XYPI (55.5%) (Table 4; Figure 4).

#### 3.4.5 Time to relief of runny nose

A total of 1,036 patients involved in eight studies (22, 38, 61, 66, 67, 80, 98, 100) were evaluated, and the time to relief of runny nose in the case of four TCMIs and five interventions was reported. All four TCMIs were better than ribavirin (*P* < 0.05), namely, YHNI (SMD: −2.11, CI: −2.82 to −1.04), RDNI (SMD: −1.02, CI: −1.45 to −0.59), XYPI (SMD: −0.69, CI: −1.18 to −0.20), and TRQI (SMD: −0.64, CI: −1.16 to −0.12), in terms of the time to relief of runny nose. Regarding the TCMIs, YHNI was better than RDNI, XYPI, and TRQI (SMD: −1.09, CI: −1.92 to −0.26/SMD: −1.42, CI: −2.28 to −0.56/SMD: −1.47, CI: −2.35 to −0.59), which suggested that it had advantages in the relief of runny nose (Table 3). According to the results of the SUCRA ranking, YHNI (99.8%) was the best treatment, followed by RDNI (67.9%) and XYPI (43.0%) (Table 4; Figure 4).

#### 3.4.6 Time to relief of nasal congestion

A total of 4,668 patients involved in 21 studies (22, 29, 53, 54, 58, 65, 68–71, 73, 75, 77, 79, 82, 85, 86, 88, 91, 92, 95) were evaluated, and the time to relief of nasal congestion in the case of four TCMIs and five interventions was reported. Three TCMIs were better than Ribavirin (*P* < 0.05), namely, YHNI (SMD: −1.22, CI: −1.54 to −0.89), XYPI (SMD: −1.14, CI: −1.82 to −0.45), and RDNI (SMD: −1.02, CI: −1.82 to −0.45), in terms of the time to relief of nasal congestion (Table 3). In addition, none of the differences between groups were statistically significant in pairwise comparisons between the three TCMIs (*P* > 0.05). According to the results of the SUCRA ranking, YHNI (78.9%) was the best treatment, followed by XYPI (68.3%) and RDNI (52.8%) (Table 4; Figure 4).

### 3.5 GRADE levels of evidence

The results of evaluation using GRADE-profiler software showed that the levels of evidence for the interventions were low or very low across the studies (Supplementary Table S11: Pages 74–S78). The included studies were only from China, and the risk of bias was increased because most of the studies did not mention blinding and allocation concealment. Moreover, the heterogeneity of the included studies and the differences in sample size caused inconsistency and imprecision, which resulted in serious indirect effects. Therefore, the results reported in this NMA should be viewed with caution.

### 3.6 Adverse reactions

Twenty-eight RCTs (22–24, 26, 28, 34, 35, 47, 49, 50, 52, 65–69, 71–75, 79, 80, 86, 88, 90, 95, 97, 101) reported the safety of TCMIs and specific adverse reactions, including diarrhea, vomiting, rash at the injection site, loss of appetite, allergies, abdominal pain, laryngitis, and headache. Only a descriptive analysis was performed because the descriptive criteria in the various studies were not uniform. The specific information is given in Table 5.

**Table 5:** Summary of adverse drug events.

Types of interventions	Number of RCTs	Group	Total sample size	Incidence	Details of adverse drug events, with numbers of cases
XYPI vs. Ribavirin	10	XYPI	509	4.13%	17 diarrhea, 2 vomiting, 2 rash at the injection site
		Ribavirin	509	7.47%	9 vomiting, 14 diarrhea, 6 loss of appetite, 7 rash at the injection site
QKLI vs. Ribavirin	1	QKLI	100	1%	1 allergies
		Ribavirin	100	1%	1 rash at the injection site
RDNI vs. Ribavirin	9	RDNI	576	2.95%	4 diarrhea, 6 vomiting, 5 rash at the injection site, 2 allergies
		Ribavirin	574	9.58%	23 vomiting, 17 rash at the injection site, 3 abdominal pain, 10 diarrhea, 2 laryngitis
YHNI vs. Ribavirin	6	YHNI	1,476	1.02%	1 headache, 5 rash at the injection site, 4 vomiting, 5 diarrhea
		Ribavirin	1,475	2.91%	13 diarrhea, 14 rash at the injection site, 9 vomiting, 3 headache, 2 abdominal pain
TRQI vs. Ribavirin	2	TRQI	40	7.5%	2 headache, 3 diarrhea
		Ribavirin	40	25%	4 headache, 6 diarrhea

### 3.7 Publication bias

**Figure 5:**
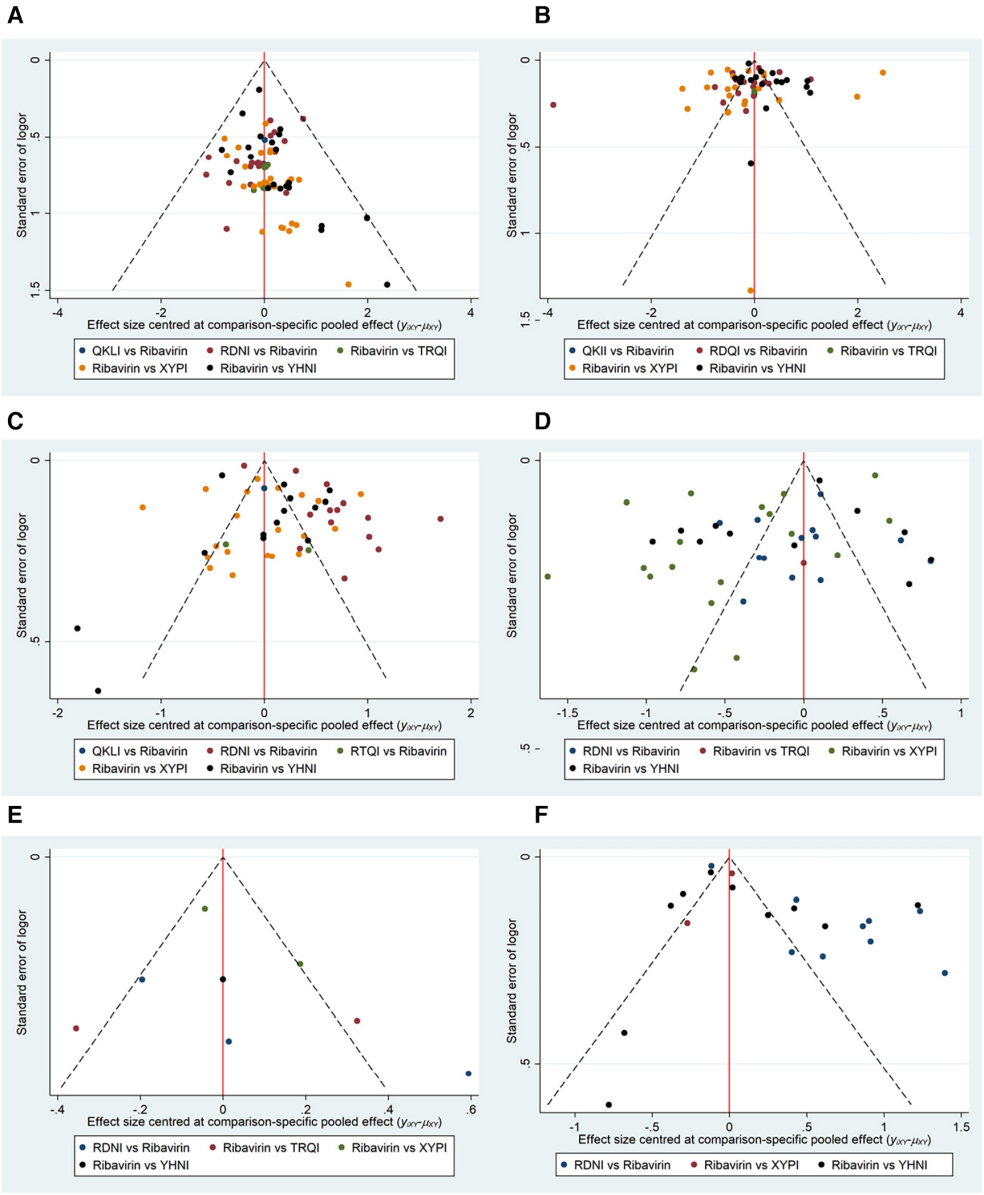
Funnel plots for the outcome indicators. OR, odds ratio. (**A**) Total effectiveness rate; (**B**) time to relief of fever; (**C**) time to relief of cough; (**D**) Time to relief of sore throat; (**E**) time to relief of runny nose; (**F**) time to relief of nasal congestion.

Comparison–correction funnel plotting for different outcome indicators was performed using Stata 15.0 software. In this study, funnel plots for the total effectiveness rate, time to relief of fever, time to relief of cough, time to relief of sore throat, time to relief of runny nose, and time to relief of nasal congestion were plotted. In combination with the results of Egger's test, the results showed that the distributions of the funnel plots for clinical effectiveness (Figure 5A) and time to relief of fever (Figure 5B) were roughly symmetric, without obvious small-sample effects or publication bias. The symmetries of the studies included with regard to time to relief of cough (Figure 5C), time to relief of sore throat (Figure 5D), time to relief of runny nose (Figure 5E), and time to relief of nasal congestion (Figure 5F) were off-axis, and *P* < 0.05 was obtained by Egger's test. This suggests that publication bias was present.

## 4 Discussion

### 4.1 Discussion of the results

The main ingredient of YHNI is dehydrated andrographolide, which is extracted from Andrographis paniculata and can play a very good role in detoxifying and clearing heat. It can therefore be used as a drug for the treatment of children with acute-phase viral upper respiratory tract infections. Modern pharmacological studies have found that YHNI has a strong antiviral effect and is capable of inactivating adenoviruses, influenza viruses, respiratory viruses, etc., and at the same time it can strengthen the body's immune ability to a certain extent (96). The principle behind the antiviral activity of YHNI may be that the monopotassium salt of dehydrated andrographolide succinate occupies the DNA–protein binding site during viral replication and therefore prevents viral replication. As shown in the results of this NMA, intravenous infusion of YHNI for the treatment of children with acute-phase viral upper respiratory tract infections was effective in relieving clinical symptoms such as nasal congestion and runny nose. In addition, some studies have indicated (102) that YHNI can strengthen the immune ability of sick children and avoid the occurrence of febrile convulsions due to hyperthermia, which damage the cerebral nerves of children with acute-phase viral upper respiratory tract infections. Clinically, ribavirin injection is used in combination with YHNI to treat children with acute-phase viral upper respiratory tract infections, which can achieve a better antiviral effect, and the efficacy of YHNI in clearing heat and strengthening the immune system is more suitable for treating sick children.

The main components of QKLI are bile acids, mother of pearl powder, porcine deoxycholic acid, Gardenia, buffalo horn powder, Platycodon, baicalin, and honeysuckle. It has antipyretic effects, inhibits bacterial endotoxins and endogenous pyrogens, inhibits inflammatory reactions, improves the circulation in important organs, protects brain tissues, preserves the liver, and lowers levels of enzymes. QKLI has been widely used in febrile illnesses and in the event of dizziness, such as in pediatric febrile convulsions, pneumonia, and upper respiratory tract infections (103, 104). This NMA study showed that QKLI exhibits improved clinical efficacy in alleviating febrile symptoms of AURI in children. Histamine is an autoactive substance that is produced by the enzyme histidine decarboxylase. When the body is stimulated by physical and chemical factors, mast cells degranulate and release histamine (105). Histamine and histamine receptors bind to cause an inflammatory response (106). Relevant experiments confirmed that QKLI could reduce levels of imidazoleacetic acid and thus affect fever caused by inflammation. Elevated levels of imidazoleacetic acid in the urine of rats in a fever group suggested that histamine levels increased in their bodies, and imidazoleacetic acid levels were significantly reduced after the injection of QKLI, which implied that the immunity of the body was improved (107). In conclusion, QKLI was able to significantly affect inflammation-induced fever.

TRQI is a Chinese herbal injection commonly used in China that consists of Scutellaria baicalensis, bear bile powder, goat's horn, honeysuckle, and Forsythia. It has been included in several guidelines, diagnostic and treatment protocols, and expert consensus statements and is recommended for the treatment of a variety of severe types of pneumonia, such as Middle East respiratory syndrome, dengue fever, and human infection with H7N9 avian influenza (108). TRQI can effectively inhibit the growth of Streptococcus pneumoniae and Streptococcus B haemolyticus, markedly reduce hypersensitivity and inflammatory reactions, and indirectly contribute to a decrease in C-reactive protein (CRP) levels in patients (109). Moreover, TRQI resists proinflammatory factors and has antipyretic effects, which improves the antiviral effect, accelerates the excretion of toxins from the body, and reduces the release of procalcitoninogen (PCT) while reducing the stress response of the body. Liu and Qu (110) showed that TRQI could significantly promote the phagocytosis of white blood cells (WBCs) and inhibit the activation of neutrophils in lung tissues. It thus exerts antibacterial and anti-inflammatory effects and improves the respiratory function of patients. This also explains the mechanism by which TRQI, as found in this NMA, can effectively alleviate patients' febrile symptoms and enhance the efficacy of treatment.

Our study showed that XYPI ranked second on the three outcome measures of relieving symptoms of cough and nasal congestion and improving clinical effectiveness. In recent years, Chinese medicinal preparations have achieved remarkable results in the treatment of pediatric diseases. XYPI contains total sulfonated lactones obtained from the Chinese medicinal plant Andrographis paniculata, with a clear composition. Pharmacological studies have shown that it not only has a direct inhibitory effect on a variety of respiratory viruses (111, 112) and bacteria, but also has a significant protective effect on the body. In addition, XYPI can act synergistically with antibiotics and also modulate the inflammatory response (113–116). Clinical studies have shown that the treatment of acute bronchitis with XYPI is superior to conventional or Western drug therapy in terms of overall effectiveness, alleviation of symptoms, and improvements in lung function indices (117–119), and XYPI has a favorable safety profile (120). A recent large-sample RCT study showed that intravenous infusion of XYPI for acute bronchitis significantly reduced median disease duration and median time to relief of cough (117).

The Chinese medicinal preparation RDNI has high application value and is produced from Gardenia, Artemisia, honeysuckle, and other medicinal herbs by the extraction of the effective components of the preparation. RDNI is used to clear heat and disperse wind and has detoxifying effects. It is widely used in the treatment of infectious emergencies, hand, foot, and mouth disease, and influenza, as well as viral infections, in disease rescue, and treatment can be effective in increasing the rate of cure and alleviating the patient's clinical symptoms (121, 122). The results of this study indicated that RDNI can effectively alleviate the symptoms of pediatric AURI such as sore throat and runny nose. Modern pharmacology has found that Artemisia can have anti-inflammatory effects and modulate immunity, Gardenia has significant advantages in antipyretic and anti-inflammatory activity, and Honeysuckle can have antibacterial, anti-inflammatory, and antioxidant effects (123). Relevant online pharmacology and experimental validation has indicated that RDNI can downregulate the expression of inflammatory cells and proinflammatory cytokines such as human interleukin-1β, human interleukin-6, and tumor necrosis factor-α. In addition, the active complex present in honeysuckle (Lonicera) reduces Akt phosphorylation and slows down the onset of inflammation (124).

### 4.2 Relationships and comparisons with other studies

This study was the first NMA that compared the differences between TCMIs for the treatment of AURI in children. Many previous studies have simply summarized the efficacy and safety of a single TCMI (125–127) for treating AURI or the difference in efficacy between different TCM herbs (128). Those studies could not have stable quality control because of the diversity of ingredients and the variability of doses. The compositions of TCMIs are more stable than those of TCM decoctions, which has quantitative significance. We comprehensively studied the RCTs that used TCMIs in combination with ribavirin in the treatment of AURI and ranked the advantages of the different TCMIs with regard to each outcome index to guide their clinical use.

### 4.3 Implications for clinical practice

In this study, we found that QKLI can significantly improve the clinical effectiveness and is effective in alleviating fever symptoms, YHNI is effective in alleviating symptoms such as sore throat, nasal congestion, and runny nose, and TRQI is effective in alleviating cough. These TCMIs can be effective in solving different problems in AURI. No study showed the effects of a combination of multiple TCMIs in the treatment of AURI. This may be related to the complexity of the components, interactions, and other factors, which need to be further investigated in subsequent studies.

By analyzing differences in different indices of children's peripheral blood in a clinical setting, Ding and Qu concluded that the measurement of peripheral WBC counts and the lymphocyte/monocyte ratio is a key test in the diagnosis and treatment of AURI that can guide clinicians to avoid the irrational use of antimicrobial drugs. They also found that a decrease in prenatals is correlated with infection of the body and is a better indicator of the inflammation status of the child's organism than PCT and CRP (129). Therefore, it is essential to investigate the effects of TCMIs on related indices. Hou et al. concluded that RDNI can correct an imbalance in immune responses, improve immunity, reduce inflammatory responses, and thus promote disease regression. This may be related to the strong immunosuppressive and anti-inflammatory effects of *Gardenia*, honeysuckle, and *Artemisia* present in this Chinese herbal medicinal preparation (130). Wang et al. concluded that QKLI can effectively reduce WBC counts and levels of CRP, interleukin-18, and other proinflammatory factors, reduce inflammatory responses, and improve the anti-infection effect in children with AURI (131).

Diarrhea and allergic reactions are the most common adverse effects of using TCMIs. Twenty-eight RCTs included in this review described TCMIs as having mild adverse effects (22–24, 26, 28, 34, 35, 47, 49, 50, 52, 65–69, 71–75, 79, 80, 86, 88, 90, 95, 97, 101), which could be mitigated or eliminated by discontinuing the medication, decreasing the dose of the medication, or symptomatic treatment. The safety of TCMIs is greatly improved by standardizing their use in clinical applications (132). Li et al. improved the quality standard for the solubilizer polysorbate 80 in TCMIs to reduce anaphylactic reactions. However, adverse reactions in patients still need to be considered to avoid medical accidents (133).

Despite the widespread and effective use of TCMIs in clinical practice, this study found that the extracted components, complex pharmacological mechanisms, and methodological descriptions of their phytopharmacological properties are still unclear. In the future, more pharmacological and mechanistic studies of TCMIs should be conducted in accordance with the consensus recommendations (134).

### 4.4 Limitations

The following limitations existed in this study: (a) adverse reactions were poorly reported, and most of the studies did not have a clear safety assessment; (b) most of the studies were rated as “some concerns” in the risk of bias assessment, and the quality of the studies was low; (c) clinical heterogeneity occurred, which was due to differences in the doses of phytomedicine and the course of treatment; (d) all the included studies were from China; (e) some of the outcome indicators were not standardized during this NMA, such as “the unit of measurement of time, including hours and days,” which may have interfered with the final summary of the results.

## 5 Conclusions

AURI is treated with inhaled bronchodilators, nebulized adrenaline, systemic steroids, and antibiotics (135). However, because of their side effects, it is particularly important to seek more effective alternative therapies. This study showed that TCMIs provide additional benefits in children. In terms of the different outcome indicators, QKLI was more effective in alleviating fever symptoms, YHNI was more effective in alleviating symptoms of sore throat, runny nose, and nasal congestion, and TRQI was more effective in relieving cough. Despite the low incidence of adverse events, only a few studies have evaluated the safety of TCMIs. Further studies are needed to better understand TCMIs and to guide their clinical application.

## Data Availability

The datasets presented in this study can be found in online repositories. The names of the repository/repositories and accession number(s) can be found in the article/**Supplementary Material**.
