# Supplementary material for: Efficacy of five different traditional Chinese medicine injections in acute upper respiratory tract infection in children: a network meta-analysis and systematic review
Source: Front Pediatr. 2024 Jun 10;12:1358639. doi: 10.3389/fped.2024.1358639 (PMC11194337; doi:10.3389/fped.2024.1358639)
Supplement: Supplementary file 1 [file Datasheet1.pdf]

SUPPLEMENTS

Supplementary Table S1-S8: Search Strategy 3

Table S1. Search Strategy: PubMed 3

Table S2. Search Strategy: Embase 3

Table S3. Search Strategy: Cochrane Library 4

Table S4. Search Strategy: Web of Science 4

Table S5. Search Strategy: SinoMed 4

Table S6. Search Strategy: China National Knowledge Infrastructure (CNKI) 6

Table S7. Search Strategy: Wanfang database 6

Table S8. Search Strategy: the Chinese Scientific Journal database (VIP)........ 6

Supplementary Table S9: Summary of All Included Studie 7

Supplementary Figure S1 Risk of Bias of Included Studies 10

Supplementary Table S10 More details about risk of bias 11

Supplementary Table S11: GRADE assessment 75

Supplementary Table S12: The PRISMA checklist of this meta-analysis 79

Table S1. Search Strategy: PubMed

#1	"Respiratory Tract Infections"[Mesh]	627062
#2	((((((((((((((Infection, Respiratory Tract[Title/Abstract])) OR (Respiratory Tract Infection[Title/Abstract])) OR (Infections, Respiratory[Title/Abstract])) OR (Infections, Respiratory Tract[Title/Abstract])) OR (Respiratory System Infections[Title/Abstract])) OR (Infection, Respiratory System[Title/Abstract])) OR (Respiratory System Infection[Title/Abstract])) OR (Respiratory Infections[Title/Abstract])) OR (Upper Respiratory Tract Infections[Title/Abstract])) OR (Upper Respiratory Infections[Title/Abstract])) OR (Upper Respiratory Tract Infection[Title/Abstract])) OR (Infections, Upper Respiratory Tract[Title/Abstract])) OR (Infections, Upper Respiratory[Title/Abstract])) OR (Respiratory Infection, Upper[Title/Abstract]))	35294
#3	#1 OR #2	643700
#4	Traditional Chinese medicine injections *[Title/Abstract]	58081
#5	(((((((Randomized controlled trial[Title/Abstract]) OR (Randomized controlled study[Title/Abstract])) OR (Randomized trial[Title/Abstract])) OR (Randomized study[Title/Abstract])) OR (Controlled trial[Title/Abstract])) OR (Controlled study[Title/Abstract])) OR (Randomized controlled trial[Title/Abstract])) OR (Randomized controlled study[Title/Abstract])	309814
#6	#3 AND #4 AND #5	9

Table S2. Search Strategy: Embase

#1	' Respiratory Tract Infections '/exp	
#2	'Respiratory Tract Infection':ab,ti,kw	
#3	'Infection, Respiratory Tract':ab,ti,kw	
#4	'Infections, Respiratory':ab,ti,kw	
#5	'Infections, Respiratory Tract':ab,ti,kw	
#6	'Respiratory System Infections':ab,ti,kw	

#7	'Infection, Respiratory System':ab,ti,kw	
#8	'Respiratory System Infection':ab,ti,kw	
#9	'Respiratory Infections':ab,ti,kw	
#10	'Upper Respiratory Tract Infections':ab,ti,kw	
#11	'Upper Respiratory Infections':ab,ti,kw	
#12	'Upper Respiratory Tract Infection':ab,ti,kw	
#13	'Infections, Upper Respiratory Tract':ab,ti,kw	
#14	'Infections, Upper Respiratory':ab,ti,kw	
#15	'Respiratory Infection, Upper':ab,ti,kw	
#16	#1 OR #2 OR #3 OR #4 OR #5 OR #6 OR #7 OR #8 OR #9 OR #10 OR #11 OR #12 OR #13 OR #14 OR #15	213367
#17	'Traditional Chinese medicine injections *':ab,ti,kw	847368
#18	' Randomized controlled trial‘ :ab,ti,kw	
#19	' Randomized controlled study ':ab,ti,kw	
#20	' Randomized trial ':ab,ti,kw	
#21	' Randomized study ':ab,ti,kw	
#22	' Controlled trial ':ab,ti,kw	
#23	' Controlled study ':ab,ti,kw	
#24	#17 OR #18 OR #19 OR #20 OR #21 OR #22 OR #23	120632
#25	#16 AND #17 AND #24	31

Table S3. Search Strategy: Cochrane Library

#1	MeSH descriptor: [Respiratory Tract Infections] explode all trees	7533
#2	(Infection, Respiratory Tract):ab,ti,kw OR (Respiratory Tract Infection):ab,ti,kw OR (Infections, Respiratory):ab,ti,kw OR (Infections, Respiratory Tract):ab,ti,kw OR (Respiratory System Infections):ab,ti,kw OR (Infection, Respiratory System):ab,ti,kw OR (Respiratory System Infection):ab,ti,kw OR (Respiratory Infections):ab,ti,kw OR (Upper Respiratory Tract Infections):ab,ti,kw OR (Upper Respiratory Infections):ab,ti,kw OR (Upper Respiratory Tract Infection):ab,ti,kw OR (Infections, Upper Respiratory Tract):ab,ti,kw OR (Infections, Upper Respiratory):ab,ti,kw OR (Respiratory Infection, Upper):ab,ti,kw	8911
#3	#1 OR #2	9833
#4	(Traditional Chinese medicine injections *):ab,ti,kw	1124
#5	(Randomized controlled trial):ab,ti,kw OR (Randomized controlled study):ab,ti,kw OR (Randomized trial):ab,ti,kw OR (Randomized study):ab,ti,kw OR (Controlled trial):ab,ti,kw OR (Controlled study):ab,ti,kw	6310
#6	#3 AND #4 AND #5	3

Table S4. Search Strategy: Web of Science

#1	(((((((((((((TS=(Respiratory Tract Infections)) OR TS=(Infection, Respiratory Tract)) OR TS=(Respiratory Tract Infection)) OR TS=(Infections, Respiratory)) OR TS=(Infections, Respiratory Tract)) OR TS=(Respiratory System Infections)) OR TS=(Infection, Respiratory System)) OR TS=(Respiratory System Infection)) OR TS=(Respiratory Infections)) OR TS=(Upper Respiratory Tract Infections)) OR TS=(Upper Respiratory Infections)) OR TS=(Upper Respiratory Tract Infection)) OR TS=(Infections, Upper Respiratory Tract)) OR TS=(Infections, Upper Respiratory)) OR TS=(Respiratory Infection, Upper)	99704
#2	TS=(Traditional Chinese medicine injections)	377824
#3	((((((TS=(Randomized controlled trial)) OR TS= TS=(Randomized controlled study)) OR TS=(Randomized trial)) OR TS=(Randomized study)) OR TS=(Controlled trial)) OR TS=(Controlled study)	302372
#4	#1 AND #2 AND #3	2

Table S5. Search Strategy: SinoMed

#1	（"上呼吸道感染"[常用字段:智能] OR "呼吸道感染"[常用字段:智能] OR "急性上呼吸道感染"[常用字段:智能] OR "急性呼吸道感染"[常用字段:智能]）AND（"中药注射液"[常用字段:智能] OR "中草药注射剂"[常用字段:智能] OR "中药注射"[常用字段:智能]）AND (“随机对照实验“[常用字段:只能] OR “随机对照研究”[常用字段:智能] OR “对照实验”[常用字段:智能] OR “随机研究”[常用字段:智能])	31

Table S6. Search Strategy: China National Knowledge Infrastructure (CNKI)

#1	（篇关摘：上呼吸道感染 + 急性上呼吸道感染 + 儿童上呼吸道感染 + 儿童急性上呼吸道感染(精确)）AND（篇关摘：中药注射 + 中草药注射液 + 中医药注射剂 + 中医注射药物 + 中药注射治疗(精确)））AND（摘要：随机对照实验(精确)）OR（摘要：随机对照研究(精确)）OR（摘要：RCT(精确)）OR（摘要：随机对照(精确)）OR（摘要：随机(精确)）AND（篇关摘：儿童(精确)）OR（篇关摘：小儿(精确)）	198

Table S7. Search Strategy: Wanfang database

#1	((主题=中药注射 + 中草药注射液 + 中医药注射剂 + 中医注射药物 + 中药注射治疗) AND (主题=上呼吸道感染+急性上呼吸道感染+儿童上呼吸道感染+儿童急性上呼吸道感染) AND (主题=随机对照研究+随机对照实验+随机对照研究+随机对照+随机))	4

Table S8. Search Strategy: the Chinese Scientific Journal database (VIP)

#1	任意字段=上呼吸道感染+急性上呼吸道感染+儿童上呼吸道感染+儿童急性上呼吸道感染	79
#2	任意字段=中药注射 + 中草药注射液 + 中医药注射剂 + 中医注射药物 + 中药注射治疗	30
#3	任意字段=随机对照研究 + 随机对照实验 + 随机对照 + 随机	102
#4	#1 AND #2 AND #3	0

Supplementary table S9: Summary of All Included Studies

No	Study	Duration of xxx 病程		Sample size		Sex(M/F)		Age(mean or range)		Interventions			Outcomes
		T	C	T	C	T	C	T	C	T	C		
1	Zhaohongxia(2015)	2.3±0.3d	2.1±0.2d	55	55	29/26	27/28	4.4±0.6	4.5±0.7	XYPI(5mg/kg/d)	R(10mg/kg/d)	3d	①②③④
2	Lixinhua(2013)	NA		50	50	65/35		8.0±2.6		XYPI(200mg/d)+ R(500mg/d)	R(500mg/d)	5d	①②③⑤⑥⑦
3	Zhengjianxin(2013)	12.1±2.5h		68	68	78/58		2.3±1.9		XYPI(5mg/kg/d)	R(10mg/kg/d)	7d	②③④⑦
4	Mali(2013)	1~3d		50	50	56/44		6.39±2.14		XYPI(5mg/kg/d)	R(10mg/kg/d)	5d	①②③④⑦
5	Zhaoruijing(2013)	NA		125	125	68/57	67/58	4.0±1.3	4.2±1.4	XYPI(5mg/kg/d)	R(10mg/kg/d)	7d	①②③④
6	Huangwenjing(2014)	3~5d		60	60	64/56		NA		XYPI(5mg/kg/d)	R(10mg/kg/d)	5d	①②④⑦
7	Zhaoyajuan(2014)	1.8±0.8d		60	60	70/50		4.5±1.5		XYPI(0.2~0.4ml/kg/d)	R(10mg/kg/d)	3d	①②
8	Zhangzhouhui(2013)	3.1±0.7d	3.3±0.6d	50	50	23/27	22/28	6.1±1.1	5.5±1.4	XYPI(6mg/kg/d)	R(9mg/kg/d)	7d	①②③④⑦
9	Jiangfei(2014)	5~48h		48	48	54/42		2.6±1.3		XYPI(5mg/kg/d)	R(10mg/kg/d)	5d	①②③④⑥
10	Maolinlin(2021)	8.3±2.1d	8.1±2.1d	35	35	22/13	21/14	7.2±2.3	7.2±2.2	XYPI(6mg/kg/d)	R(9mg/kg/d)	5d	①②③
11	Dinglihong(2014)	NA		25	25	32/18		3~10		XYPI(0.2~0.4ml/kg/d)	R(10mg/kg/d)	7d	①
12	Panjie(2017)	12.9±2.7h	13.2±2.5h	49	49	26/23	28/21	2.2±1.2	2.4±1.0	XYPI(0.2~0.4ml/kg/d)	R(10mg/kg/d)	3d	①②③④
13	Liuji(2016)	2~13h		49	49	54/44		0.5~4		XYPI(5~10mg/kg/d)	R(10mg/kg/d)	5d	①②③④
14	Liujiarong(2016)	NA		30	30	17/13	19/11	2.6±1.4	2.8±1.2	XYPI(5mg/kg/d)	R(10mg/kg/d)	5d	①⑦
15	Moguipei(2016)	NA		40	40	24/16	27/13	4.2±1.6	4.4±1.7	XYPI(0.2~0.4ml/kg/d)	R(10mg/kg/d)	7d	①②③④⑦
16	Wulin(2017)	1.9±0.5d		46	46	49/43		4.5±1.7		XYPI(5mg/kg/d)	R(10mg/kg/d)	7d	①②③④
17	Chenhaijun(2015)	NA		50	50	54/46		4.5±0.8		XYPI(0.2~0.4ml/kg/d)	R(10mg/kg/d)	7d	①
18	Shenguijun(2017)	12.6±3.2h	12.2±3.5h	48	48	25/23	26/22	2.4±1.1	2.5±1.3	XYPI(5mg/kg/d)	R(10mg/kg/d)	7d	①②③④⑤

19	Qinyan(2016)	NA		42	42	22/20	21/21	5.8±1.3	5.5±1.2	XYPI(5mg/kg/d)	R(10mg/kg/d)	5d	①②③④
20	Wangyan(2015)	NA		100	96	38/62	43/53	5.1±0.4	4.7±0.6	XYPI(0.2~0.4ml/kg/d)	R(10mg/kg/d)	7d	①②③④
21	Zhulili(2016)	2.5d	2.0d	98	98	59/39	56/42	2.4±1.5	3.3±1.5	XYPI(5~10mg/kg/d)	R(10mg/kg/d)	7d	①②③
22	Liuguifang(2015)	NA		50	50	32/18	31/19	7.2±2.1	7.5±2.3	XYPI(5~10mg/kg/d)	R(10mg/kg/d)	5d	①
23	Gongtianyin(2016)	NA		98	91	53/46	51/40	3.2	3.8	XYPI(5mg/kg/d)	R(10mg/kg/d)	5d	①
24	Ganxiaohong(2014)	NA		60	60	29/31	28/32	3.5±0.9	3.7±0.8	XYPI(0.2~0.4ml/kg/d)	R(10mg/kg/d)	5d	①②③④
25	Hanchangming(2016)	5.6±1.2d	5.8±1.2d	50	50	25/25	26/24	5.6±1.4	5.8±1.4	XYPI(0.2~0.4ml/kg/d)	R(10mg/kg/d)	5d	①
26	Zhangyuqin(2018)	NA		40	40	59/31		4.3±2.1		XYPI(200mg/d)	R(500mg/d)	5d	①②③④
27	Yanghongying(2015)	NA		41	41	22/19	23/18	5.3±1.1	5.6±1.3	XYPI(5mg/kg/d)	R(10mg/kg/d)	5d	①②③④⑦
28	Zhangjun(2015)	NA		44	44	24/20	20/24	3.5	3.6	XYPI(5mg/kg/d)	R(10mg/kg/d)	5d	②
29	Zhumantang(2017)	1.7±0.6	1.6±0.5	60	60	39/21	37/23	5.8±0.9	5.7±0.8	XYPI(5mg/kg/d)	R(10mg/kg/d)	5d	①⑦
30	Yijingting(2016)	3.0±1.2	3.0±1.5	60	60	39/21	33/27	4.0±1.1	3.0±1.6	XYPI(5~10mg/kg/d)	R(10mg/kg/d)	7d	①②③④⑦
31	Guoyewei(2014)	1.8±0.4	1.6±0.6	50	50	27/23	29/21	1.0±0.6	1.0±0.7	QKLI(0.8~1ml/kg/d)	R(10mg/kg/d)	5d	①
32	Panhong(2015)	NA		100	100	52/48	50/50	5.6±2.3	5.9±2.5	QKLI(0.5~0.6ml/kg/d)	R(10mg/kg/d)	7d	①②③⑦
33	Liuqin(2015)	NA		61	61	30/31	29/32	5.4±1.5	5.5±1.3	RDNI(0.6ml/kg/d)	R(10mg/kg/d)	3d	①②③④⑥
34	Zhangxian(2013)	NA		68	61	NA		1~6		RDNI(0.5~0.8ml/kg/d)	R(10mg/kg/d)	3d	①②③④⑥
35	Dingpei(2013)	NA		58	58	61/55		1~12		RDNI(0.3~0.5ml/kg/d)	R(10mg/kg/d)	5d	①②③④
36	Liuhui(2015)	24~72h		62	61	65/58		10m~11y		RDNI(0.3~0.5ml/kg/d)	R(10mg/kg/d)	5d	①
37	Zhouwenwen(2014)	< 48h		42	42	20/22	19/21	NA		RDNI(0.3~0.5ml/kg/d)	R(10mg/kg/d)	3d	①
38	Cuibeiyong(2013)	NA		120	110	62/58	53/57	3m~3y		RDNI(0.5~0.8ml/kg/d)	R(10mg/kg/d)	5d	①③⑥
39	Liuhaiqin(2013)	2.7±0.5	2.5±0.6	38	38	21/17	23/15	4.4±0.5	4.2±0.6	RDNI(0.5ml/kg/d)	R(10mg/kg/d)	7d	①②
40	Huchunxia(2014)	NA		150	150	75/75	75/75	6m~6y		RDNI(0.5ml/kg/d)	R(10mg/kg/d)	7d	①②③
41	Liumingqi（2014）	NA		104	104	118/90		< 18y		RDNI(0.5~1ml/kg/d)	R(10mg/kg/d)	5d	①②④⑤
42	Limingchun(2014)	< 24h		84	84	44/40	45/39	11m~5y	10m~5y	RDNI(0.5ml/kg/d)	R(10mg/kg/d)	4d	①②③④⑦

43	Guyuxing(2013)	NA		120	120	80/40	78/42	7.2±1.2	7.1±1.6	RDNI(0.5~0.8ml/kg/d)	R(10mg/kg/d)	5d	①②⑦
44	Muchunjie(2013)	NA		68	68	38/30	36/32	5.2±1.4	5.3±1.4	RDNI(0.6ml/kg/d)	R(10mg/kg/d)	3d	①
45	Zhaoqun(2018)	2.2±0.3	2.3±0.1	45	45	28/17	27/18	6.2±2.6	6.3±2.5	RDNI(0.6ml/kg/d)	R(10mg/kg/d)	4d	①②③④⑥⑦
46	Xudongsheng(2015)	NA		90	90	54/36	46/44	7.3±1.2	7.4±1.1	RDNI(0.6ml/kg/d)	R(10mg/kg/d)	4d	①②③④⑤⑦
47	Zhuangtao(2015)	2.2±0.3d	2.2±0.4d	39	39	21/18	20/19	5.1±1.2	5.2±1.2	RDNI(0.5ml/kg/d)	R(10mg/kg/d)	3d	①②③④⑤⑦
48	Fangwanfen(2015)	1.7±0.8d	1.6±0.8d	59	59	32/27	30/29	6.0±1.6	5.8±1.7	RDNI(0.6ml/kg/d)	R(10mg/kg/d)	3d	①②③④⑥⑦
49	Lihao(2017)	50.4±10.6h	50.5±10.2h	34	34	18/16	19/15	8.5±2.4	8.3±2.7	RDNI(0.6ml/kg/d)	R(10mg/kg/d)	3d	①②③④⑥⑦
50	Zhangzhigang(2019)	NA		43	43	25/18	26/17	4.9±4.0	5.4±4.6	RDNI(0.6ml/kg/d)	R(10mg/kg/d)	3d	①②③④⑥
51	Zhangjie(2015)	NA		60	60	32/28	31/29	6.4±1.2	6.5±1.0	RDNI(0.6ml/kg/d)	R(10mg/kg/d)	3d	①②③④⑥⑦
52	Liuyan(2015)	2.1±0.9d	2.4±0.8d	45	45	25/20	24/21	3.3±2.2	3.1±2.3	RDNI(0.5~0.8ml/kg/d)	R(10mg/kg/d)	3d	①
53	Liubinxiu(2015)	< 48h		40	40	20/20	22/18	3.4±1.7	3.4±1.9	RDNI(10ml/d)	R(10mg/kg/d)	3d	①②③④⑥
54	Liuzhongyan(2015)	NA		104	102	NA		NA		RDNI(0.5~0.8ml/kg/d)	R(10mg/kg/d)	3d	①②⑦
55	Weiyueru(2014)	1.8±0.6		102	101	106/97		3.2±1.6		YHNI(5~10mg/kg/d)	R(10mg/kg/d)	3d	①②③④⑥⑦
56	Wangguiying(2014)	NA		50	50	29/31	25/25	NA		YHNI(5~10mg/kg/d)	R(10mg/kg/d)	3d	①②
57	Wuguobin(2013)	4d		25	25	28/22		4.8±1.2		YHNI(5~10mg/kg/d)	R(10mg/kg/d)	3d	①②③⑥
58	Saiqin(2015)	NA		40	40	23/17	19/21	3.8±1.6	3.7±1.5	YHNI(10mg/kg/d)	R(10mg/kg/d)	3d	①
59	Mushuyun(2013)	4d~2m		50	50	56/44		7m~9y		YHNI(5~10mg/kg/d)	R(10mg/kg/d)	3d	①②③⑥⑦
60	Wangxinlian(2019)	2.3±0.7	2.5±1.1	95	95	52/43	45/46	5.3±1.2	5.6±1.1	YHNI(0.16~0.4g/d)	R(10mg/kg/d)	3d	①②③④⑤⑦
61	Miuchunjie(2013)	NA		60	60	34/26	33/27	5.2±1.4	5.3±1.4	YHNI(40~80mg/d)	R(10mg/kg/d)	3d	①
62	Tangliqun(2013)	4.2±0.5d	4.0±0.7d	105	105	61/44	65/40	4.5±0.6	4.7±0.8	YHNI(5~10mg/kg/d)	R(10mg/kg/d)	3d	①②③④⑥
63	Kuangchaobo(2014)	20.56±2.33h		60	60	64/56		4.56±0.33		YHNI(240mg/d)	R(10mg/kg/d)	3d	①
64	Tanghongjun(2015)	NA		82	44	48/34	23/21	48.9±2.8m	48.5±2.6m	YHNI(5~10mg/kg/d)	R(10mg/kg/d)	7d	①
65	Jifengying(2014)	NA		103	103	54/49	53/50	3.0±1.5	3.1±1.3	YHNI(10mg/kg/d)	R(10mg/kg/d)	3d	①②③④⑥
66	Liuaipeng(2014)	3.9±0.2d	3.9±0.2	141	141	72/69	73/68	4.1±0.7	4.2±0.8	YHNI(10mg/kg/d)	R(10mg/kg/d)	3d	①②③④⑥⑦

67	Zhangjunjing(2017)	3.5±1.3	3.4±1.2	40	40	24/16	22/18	5.6±1.1	5.8±1.0	YHNI(5~10mg/kg/d)	R(10mg/kg/d)	3d	①②
68	Baoliang(2016)	6~48h		58	58	76/40		1~6y		YHNI(0.16g/d)	R(10mg/kg/d)	7d	②③④⑥⑦
69	Zhaona(2015)	NA		40	40	22/18	21/19	NA		YHNI(5mg/kg/d)	R(10mg/kg/d)	7d	①
70	Lvhuanming(2019)	3.2±1.0	3.1±1.1d	30	30	18/12	17/13	5.2±1.3	5.1±1.3	YHNI(5~10mg/kg/d)	R(10mg/kg/d)	5d	①②③④⑦
71	Zhuyuemei(2017)	1.8±0.3	1.7±0.4	45	45	25/20	26/19	5.2±1.2	5.2±1.2	YHNI(160~400mg/d)	R(10mg/kg/d)	3d	①②③④⑥
72	Lixiaoli(2018)	NA		39	39	21/18	20/19	2~6y		YHNI(5~10mg/kg/d)	R(10mg/kg/d)	3d	①②
73	Yanghonggui(2015)	3.5±0.5d	4.0±0.7	86	86	51/35	49/37	3.5±0.6	3.7±0.8	YHNI(5~10mg/kg/d)	R(10mg/kg/d)	3d	①②③④⑥
74	Lijiahong(2015)	NA		30	30	16/14	15/15	5.3±4.2	4.8±3.6	YHNI(5~8mg/kg/d)	R(10mg/kg/d)	3d	①②③
75	Haobingfeng(2017)	< 3d		1000	1000	523/477	520/480	4.2±2.1	4.3±2.0	YHNI(10mg/kg/d)	R(10mg/kg/d)	3d	①②③④⑥⑦
76	Yanghaizhen(2023)	3.4±1.1	3.1±1.2	35	35	19/16	20/15	3.3±1.2	3.2±1.1	YHNI(160~400mg/d)	R(10mg/kg/d)	5d	①②③④
77	Cuibingzhong(2013)	2.4±1.2	2.6±1.3	40	40	21/19	18/22	6.6±2.2	6.8±2.1	TRQI(0.3~0.5ml/kg/d)	R(10mg/kg/d)	5d	①⑦
78	Wangchunying(2014)	NA		52	52	56/48		6.5±2.4		TRQI(0.3~0.5ml/kg/d)	R(10mg/kg/d)	7d	①②③⑤
79	Wanmin(2016)	NA		50	50	55/45		2~5.2y		TRQI(0.3~0.5ml/kg/d)	R(5mg/kg/d)	7d	①
80	Kongshanshan(2018)	2.0±0.6	2.2±0.4	40	40	22/18	20/20	5.3±1.2	5.8±1.3	TRQI(0.3~0.5ml/kg/d)	R(10mg/kg/d)	5d	①③④⑤
81	Guohui(2017)	NA	NA	40	40	19/21	21/19	5.0±0.5	4.2±0.5	TRQI(0.8ml/kg/d)	R(8mg/kg/d)	5d	①⑦

① Total effective rate, ② Antipyretic time, ③Time to Relief of Cough ④Time to Relief of Sore Throat ⑤Time to Relief of Runny Nose ⑥Time to Relief of Stuffy Nose ⑦Adverse reactions

XYPI=Xiyanping injection; QKLI=Qingkailing injection; RDNI=Reduning injection; YHNI=Yanhuning injection; TRQI=Tanreqing injection; R=Ribavirin injection

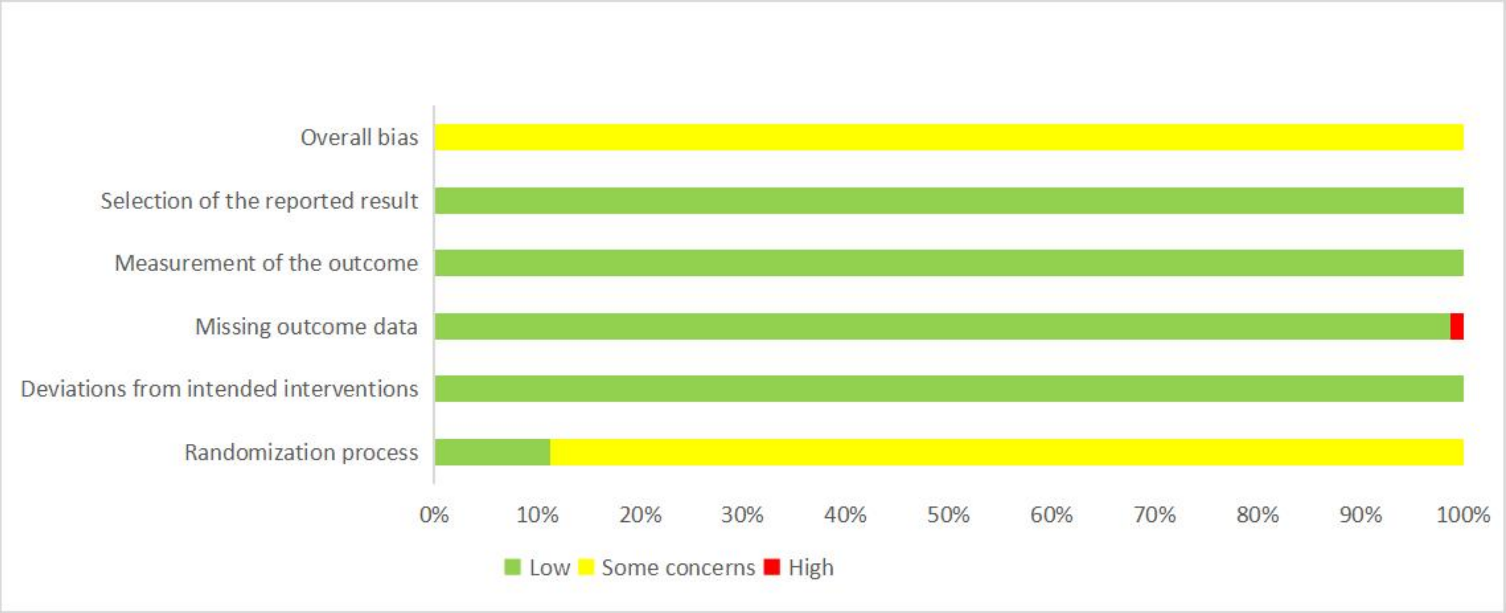

Overall bias
Selection of the reported result
Measurement of the outcome
Missing outcome data
Deviations from intended interventions
Randomization process
0%
10%
20%
30%
40%
50%
60%
70%
80%
90%
100%
Low
Some concerns
High

Supplementary table S1 More details about risk of bias

XYPI VS Ribavirin (n=30)		
Study	Zhaohongxia(2015)	
Domain	Signalling question	Responese
Bias arising from the randomization process	1.1 Was the allocation sequence random?	Y
	1.2 Was the allocation sequence concealed until participants were enrolled and assigned to interventions?	NA
	1.3 Did baseline differences between intervention groups suggest a problem with the randomization process?	NA
	**Risk of bias judgement**	Some concerns
Bias due to deviations from intended interventions	2.1.Were participants aware of their assigned intervention during the trial?	Y
	2.2.Were carers and people delivering the interventions aware of participants' assigned intervention during the trial?	Y
	2.3. If Y/PY/NI to 2.1 or 2.2: Were there deviations from the intended intervention that arose because of the experimental context?	NA
	2.4 If Y/PY to 2.3: Were these deviations likely to have affected the outcome?	NA
	2.5. If Y/PY/NI to 2.4: Were these deviations from intended intervention balanced between groups?	NI
	2.6 Was an appropriate analysis used to estimate the effect of assignment to intervention?	NI
	2.7 If N/PN/NI to 2.6: Was there potential for a substantial impact (on the result) of the failure to analyse participants in the group to which they were randomized?	NA
	**Risk of bias judgement**	Some concerns
Bias due to missing outcome data	3.1 Were data for this outcome available for all, or nearly all, participants randomized?	Y
	3.2 If N/PN/NI to 3.1: Is there evidence that result was not biased by missing outcome data?	NA
	3.3 If N/PN to 3.2: Could missingness in the outcome depend on its true value?	NA
	3.4 If Y/PY/NI to 3.3: Is it likely that missingness in the outcome depended on its true value?	NA
	**Risk of bias judgement**	Low
Bias in measurement of the outcome	4.1 Was the method of measuring the outcome inappropriate?	N
	4.2 Could measurement or ascertainment of the outcome have differed between intervention groups?	N
	4.3 Were outcome assessors aware of the intervention received by study participants?	N
	4.4 If Y/PY/NI to 4.3: Could assessment of the outcome have been influenced by knowledge of intervention received?	NA
	4.5 If Y/PY/NI to 4.4: Is it likely that assessment of the outcome was influenced by knowledge of intervention received?	NA
	**Risk of bias judgement**	Low
Bias in selection	5.1 Were the data that produced this result analysed in accordance with a pre-specified analysis plan that was finalized before unblinded outcome data were available for analysis?	Y

[illegible] of the reported result	5.2 ... multiple eligible outcome measurements (e.g. scales, definitions, time points) within the outcome domain?	Y
	5.3 ... multiple eligible analyses of the data?	NA
	**Risk of bias judgement**	Some concerns
Overall bias	**Risk of bias judgement**	Some concerns
Study	Lixinhua(2013)	
Domain	Signalling question	Responese
Bias arising from the randomization process	1.1 Was the allocation sequence random?	Y
	1.2 Was the allocation sequence concealed until participants were enrolled and assigned to interventions?	NA
	1.3 Did baseline differences between intervention groups suggest a problem with the randomization process?	NA
	**Risk of bias judgement**	Some concerns
Bias due to deviations from intended interventions	2.1.Were participants aware of their assigned intervention during the trial?	N
	2.2.Were carers and people delivering the interventions aware of participants' assigned intervention during the trial?	N
	2.3. If Y/PY/NI to 2.1 or 2.2: Were there deviations from the intended intervention that arose because of the experimental context?	PY
	2.4 If Y/PY to 2.3: Were these deviations likely to have affected the outcome?	PY
	2.5. If Y/PY/NI to 2.4: Were these deviations from intended intervention balanced between groups?	NA
	2.6 Was an appropriate analysis used to estimate the effect of assignment to intervention?	N
	2.7 If N/PN/NI to 2.6: Was there potential for a substantial impact (on the result) of the failure to analyse participants in the group to which they were randomized?	NA
	**Risk of bias judgement**	Some concerns
Bias due to missing outcome data	3.1 Were data for this outcome available for all, or nearly all, participants randomized?	Y
	3.2 If N/PN/NI to 3.1: Is there evidence that result was not biased by missing outcome data?	NI
	3.3 If N/PN to 3.2: Could missingness in the outcome depend on its true value?	NI
	3.4 If Y/PY/NI to 3.3: Is it likely that missingness in the outcome depended on its true value?	NI
	**Risk of bias judgement**	Low
Bias in measurement of the outcome	4.1 Was the method of measuring the outcome inappropriate?	N
	4.2 Could measurement or ascertainment of the outcome have differed between intervention groups?	NA
	4.3 Were outcome assessors aware of the intervention received by study participants?	N
	4.4 If Y/PY/NI to 4.3: Could assessment of the outcome have been influenced by knowledge of intervention received?	NI
	4.5 If Y/PY/NI to 4.4: Is it likely that assessment of the outcome was influenced by knowledge of intervention received?	PY
	**Risk of bias judgement**	Some concerns
Bias in selection of the reported result	5.1 Were the data that produced this result analysed in accordance with a pre-specified analysis plan that was finalized before unblinded outcome data were	NA
	5.2 ... multiple eligible outcome measurements (e.g. scales, definitions, time points) within the outcome domain?	Y
	5.3 ... multiple eligible analyses of the data?	NA
	**Risk of bias judgement**	Some concerns
Overall bias	**Risk of bias judgement**	Some concerns

Study	Zhengjianxin(2013)	
Domain	Signalling question	Responese
Bias arising from the randomization process	1.1 Was the allocation sequence random?	Y
	1.2 Was the allocation sequence concealed until participants were enrolled and assigned to interventions?	NA
	1.3 Did baseline differences between intervention groups suggest a problem with the randomization process?	NA
	**Risk of bias judgement**	Some concerns
Bias due to deviations from intended interventions	2.1.Were participants aware of their assigned intervention during the trial?	Y
	2.2.Were carers and people delivering the interventions aware of participants' assigned intervention during the trial?	Y
	2.3. If Y/PY/NI to 2.1 or 2.2: Were there deviations from the intended intervention that arose because of the experimental context?	NA
	2.4 If Y/PY to 2.3: Were these deviations likely to have affected the outcome?	N
	2.5. If Y/PY/NI to 2.4: Were these deviations from intended intervention balanced between groups?	PY
	2.6 Was an appropriate analysis used to estimate the effect of assignment to intervention?	NI
	2.7 If N/PN/NI to 2.6: Was there potential for a substantial impact (on the result) of the failure to analyse participants in the group to which they were randomized?	PN
	**Risk of bias judgement**	Low
Bias due to missing outcome data	3.1 Were data for this outcome available for all, or nearly all, participants randomized?	Y
	3.2 If N/PN/NI to 3.1: Is there evidence that result was not biased by missing outcome data?	NA
	3.3 If N/PN to 3.2: Could missingness in the outcome depend on its true value?	NA
	3.4 If Y/PY/NI to 3.3: Is it likely that missingness in the outcome depended on its true value?	N
	**Risk of bias judgement**	Some concerns
Bias in measurement of the outcome	4.1 Was the method of measuring the outcome inappropriate?	N
	4.2 Could measurement or ascertainment of the outcome have differed between intervention groups?	N
	4.3 Were outcome assessors aware of the intervention received by study participants?	N
	4.4 If Y/PY/NI to 4.3: Could assessment of the outcome have been influenced by knowledge of intervention received?	NA
	4.5 If Y/PY/NI to 4.4: Is it likely that assessment of the outcome was influenced by knowledge of intervention received?	NA
	**Risk of bias judgement**	Low
Bias in selection of the reported result	5.1 Were the data that produced this result analysed in accordance with a pre-specified analysis plan that was finalized before unblinded outcome data were	Y
	5.2 ... multiple eligible outcome measurements (e.g. scales, definitions, time points) within the outcome domain?	Y
	5.3 ... multiple eligible analyses of the data?	NA
	**Risk of bias judgement**	Some concerns
Overall bias	**Risk of bias judgement**	Some concerns

Study	Mali(2013)	
Domain	Signalling question	Responese
Bias arising from the randomization process	1.1 Was the allocation sequence random?	Y
	1.2 Was the allocation sequence concealed until participants were enrolled and assigned to interventions?	NA
	1.3 Did baseline differences between intervention groups suggest a problem with the randomization process?	NA
	**Risk of bias judgement**	Some concerns
Bias due to	2.1.Were participants aware of their assigned intervention during the trial?	Y
	2.2.Were carers and people delivering the interventions aware of participants' assigned intervention during the trial?	N
	2.3. If Y/PY/NI to 2.1 or 2.2: Were there deviations from the intended intervention that arose because of the experimental context?	NA

[illegible] deviations from intended interventions	2.4 If Y/PY to 2.3: Were these deviations likely to have affected the outcome?	N
	2.5. If Y/PY/NI to 2.4: Were these deviations from intended intervention balanced between groups?	PY
	2.6 Was an appropriate analysis used to estimate the effect of assignment to intervention?	NI
	2.7 If N/PN/NI to 2.6: Was there potential for a substantial impact (on the result) of the failure to analyse participants in the group to which they were randomized?	NI
	**Risk of bias judgement**	Some concerns
Bias due to missing outcome data	3.1 Were data for this outcome available for all, or nearly all, participants randomized?	Y
	3.2 If N/PN/NI to 3.1: Is there evidence that result was not biased by missing outcome data?	NA
	3.3 If N/PN to 3.2: Could missingness in the outcome depend on its true value?	NA
	3.4 If Y/PY/NI to 3.3: Is it likely that missingness in the outcome depended on its true value?	N
	**Risk of bias judgement**	Low
Bias in measurement of the outcome	4.1 Was the method of measuring the outcome inappropriate?	N
	4.2 Could measurement or ascertainment of the outcome have differed between intervention groups?	N
	4.3 Were outcome assessors aware of the intervention received by study participants?	N
	4.4 If Y/PY/NI to 4.3: Could assessment of the outcome have been influenced by knowledge of intervention received?	NA
	4.5 If Y/PY/NI to 4.4: Is it likely that assessment of the outcome was influenced by knowledge of intervention received?	NA
	**Risk of bias judgement**	Low
Bias in selection of the reported result	5.1 Were the data that produced this result analysed in accordance with a pre-specified analysis plan that was finalized before unblinded outcome data were	Y
	5.2 ... multiple eligible outcome measurements (e.g. scales, definitions, time points) within the outcome domain?	Y
	5.3 ... multiple eligible analyses of the data?	NA
	**Risk of bias judgement**	Some concerns
Overall bias	**Risk of bias judgement**	Some concerns
Study	Zhaoruijing(2013)	
Domain	Signalling question	Responese
Bias arising from the randomization process	1.1 Was the allocation sequence random?	Y
	1.2 Was the allocation sequence concealed until participants were enrolled and assigned to interventions?	NA
	1.3 Did baseline differences between intervention groups suggest a problem with the randomization process?	NA
	**Risk of bias judgement**	Some concerns
Bias due to deviations from intended interventions	2.1.Were participants aware of their assigned intervention during the trial?	Y
	2.2.Were carers and people delivering the interventions aware of participants' assigned intervention during the trial?	Y
	2.3. If Y/PY/NI to 2.1 or 2.2: Were there deviations from the intended intervention that arose because of the experimental context?	NA
	2.4 If Y/PY to 2.3: Were these deviations likely to have affected the outcome?	N
	2.5. If Y/PY/NI to 2.4: Were these deviations from intended intervention balanced between groups?	Y
	2.6 Was an appropriate analysis used to estimate the effect of assignment to intervention?	NA
	2.7 If N/PN/NI to 2.6: Was there potential for a substantial impact (on the result) of the failure to analyse participants in the group to which they were randomized?	NI
	**Risk of bias judgement**	Low
Bias due to missing [illegible]	3.1 Were data for this outcome available for all, or nearly all, participants randomized?	N
	3.2 If N/PN/NI to 3.1: Is there evidence that result was not biased by missing outcome data?	PN
	3.3 If N/PN to 3.2: Could missingness in the outcome depend on its true value?	NA
	3.4 If Y/PY/NI to 3.3: Is it likely that missingness in the outcome depended on its true value?	N

outcome data	**Risk of bias judgement**	Some concerns
Bias in measurement of the outcome	4.1 Was the method of measuring the outcome inappropriate?	N
	4.2 Could measurement or ascertainment of the outcome have differed between intervention groups?	N
	4.3 Were outcome assessors aware of the intervention received by study participants?	N
	4.4 If Y/PY/NI to 4.3: Could assessment of the outcome have been influenced by knowledge of intervention received?	PY
	4.5 If Y/PY/NI to 4.4: Is it likely that assessment of the outcome was influenced by knowledge of intervention received?	NA
	**Risk of bias judgement**	Some concerns
Bias in selection of the reported result	5.1 Were the data that produced this result analysed in accordance with a pre-specified analysis plan that was finalized before unblinded outcome data were	Y
	5.2 ... multiple eligible outcome measurements (e.g. scales, definitions, time points) within the outcome domain?	Y
	5.3 ... multiple eligible analyses of the data?	NA
	**Risk of bias judgement**	Some concerns
Overall bias	**Risk of bias judgement**	Some concerns
Study	Huangwenjing(2014)	
Domain	Signalling question	Responese
Bias arising from the randomization process	1.1 Was the allocation sequence random?	Y
	1.2 Was the allocation sequence concealed until participants were enrolled and assigned to interventions?	NA
	1.3 Did baseline differences between intervention groups suggest a problem with the randomization process?	NA
	**Risk of bias judgement**	Some concerns
Bias due to deviations from intended interventions	2.1.Were participants aware of their assigned intervention during the trial?	NA
	2.2.Were carers and people delivering the interventions aware of participants' assigned intervention during the trial?	NA
	2.3. If Y/PY/NI to 2.1 or 2.2: Were there deviations from the intended intervention that arose because of the experimental context?	NI
	2.4 If Y/PY to 2.3: Were these deviations likely to have affected the outcome?	NA
	2.5. If Y/PY/NI to 2.4: Were these deviations from intended intervention balanced between groups?	NI
	2.6 Was an appropriate analysis used to estimate the effect of assignment to intervention?	NA
	2.7 If N/PN/NI to 2.6: Was there potential for a substantial impact (on the result) of the failure to analyse participants in the group to which they were randomized?	NI
	**Risk of bias judgement**	Some concerns
Bias due to missing outcome data	3.1 Were data for this outcome available for all, or nearly all, participants randomized?	N
	3.2 If N/PN/NI to 3.1: Is there evidence that result was not biased by missing outcome data?	PN
	3.3 If N/PN to 3.2: Could missingness in the outcome depend on its true value?	NA
	3.4 If Y/PY/NI to 3.3: Is it likely that missingness in the outcome depended on its true value?	N
	**Risk of bias judgement**	Some concerns
Bias in measurement of the outcome	4.1 Was the method of measuring the outcome inappropriate?	N
	4.2 Could measurement or ascertainment of the outcome have differed between intervention groups?	N
	4.3 Were outcome assessors aware of the intervention received by study participants?	N
	4.4 If Y/PY/NI to 4.3: Could assessment of the outcome have been influenced by knowledge of intervention received?	PY
	4.5 If Y/PY/NI to 4.4: Is it likely that assessment of the outcome was influenced by knowledge of intervention received?	NA

	**Risk of bias judgement**	Some concerns
Bias in selection of the reported result	5.1 Were the data that produced this result analysed in accordance with a pre-specified analysis plan that was finalized before unblinded outcome data were	Y
	5.2 ... multiple eligible outcome measurements (e.g. scales, definitions, time points) within the outcome domain?	Y
	5.3 ... multiple eligible analyses of the data?	NA
	**Risk of bias judgement**	Some concerns
Overall bias	**Risk of bias judgement**	Some concerns
Study	Zhaoyajuan(2014)	
Domain	Signalling question	Responese
Bias arising from the randomization process	1.1 Was the allocation sequence random?	Y
	1.2 Was the allocation sequence concealed until participants were enrolled and assigned to interventions?	NA
	1.3 Did baseline differences between intervention groups suggest a problem with the randomization process?	NA
	**Risk of bias judgement**	Some concerns
Bias due to deviations from intended interventions	2.1.Were participants aware of their assigned intervention during the trial?	Y
	2.2.Were carers and people delivering the interventions aware of participants' assigned intervention during the trial?	Y
	2.3. If Y/PY/NI to 2.1 or 2.2: Were there deviations from the intended intervention that arose because of the experimental context?	NI
	2.4 If Y/PY to 2.3: Were these deviations likely to have affected the outcome?	PY
	2.5. If Y/PY/NI to 2.4: Were these deviations from intended intervention balanced between groups?	Y
	2.6 Was an appropriate analysis used to estimate the effect of assignment to intervention?	Y
	2.7 If N/PN/NI to 2.6: Was there potential for a substantial impact (on the result) of the failure to analyse participants in the group to which they were randomized?	NI
	**Risk of bias judgement**	Low
Bias due to missing outcome data	3.1 Were data for this outcome available for all, or nearly all, participants randomized?	Y
	3.2 If N/PN/NI to 3.1: Is there evidence that result was not biased by missing outcome data?	PN
	3.3 If N/PN to 3.2: Could missingness in the outcome depend on its true value?	NA
	3.4 If Y/PY/NI to 3.3: Is it likely that missingness in the outcome depended on its true value?	N
	**Risk of bias judgement**	Low
Bias in measurement of the outcome	4.1 Was the method of measuring the outcome inappropriate?	N
	4.2 Could measurement or ascertainment of the outcome have differed between intervention groups?	NA
	4.3 Were outcome assessors aware of the intervention received by study participants?	N
	4.4 If Y/PY/NI to 4.3: Could assessment of the outcome have been influenced by knowledge of intervention received?	PY
	4.5 If Y/PY/NI to 4.4: Is it likely that assessment of the outcome was influenced by knowledge of intervention received?	NA
	**Risk of bias judgement**	Low
Bias in selection of the reported result	5.1 Were the data that produced this result analysed in accordance with a pre-specified analysis plan that was finalized before unblinded outcome data were	Y
	5.2 ... multiple eligible outcome measurements (e.g. scales, definitions, time points) within the outcome domain?	Y
	5.3 ... multiple eligible analyses of the data?	NA
	**Risk of bias judgement**	Some concerns
Overall bias	**Risk of bias judgement**	Some concerns

Study	Zhangzhouhui(2013)	
Domain	Signalling question	Responese
Bias arising from the randomization process	1.1 Was the allocation sequence random?	Y
	1.2 Was the allocation sequence concealed until participants were enrolled and assigned to interventions?	NA
	1.3 Did baseline differences between intervention groups suggest a problem with the randomization process?	NA
	**Risk of bias judgement**	Some concerns
Bias due to deviations from intended interventions	2.1.Were participants aware of their assigned intervention during the trial?	Y
	2.2.Were carers and people delivering the interventions aware of participants' assigned intervention during the trial?	Y
	2.3. If Y/PY/NI to 2.1 or 2.2: Were there deviations from the intended intervention that arose because of the experimental context?	NI
	2.4 If Y/PY to 2.3: Were these deviations likely to have affected the outcome?	PY
	2.5. If Y/PY/NI to 2.4: Were these deviations from intended intervention balanced between groups?	Y
	2.6 Was an appropriate analysis used to estimate the effect of assignment to intervention?	Y
	2.7 If N/PN/NI to 2.6: Was there potential for a substantial impact (on the result) of the failure to analyse participants in the group to which they were randomized?	NI
	**Risk of bias judgement**	Low
Bias due to missing outcome data	3.1 Were data for this outcome available for all, or nearly all, participants randomized?	Y
	3.2 If N/PN/NI to 3.1: Is there evidence that result was not biased by missing outcome data?	PN
	3.3 If N/PN to 3.2: Could missingness in the outcome depend on its true value?	NA
	3.4 If Y/PY/NI to 3.3: Is it likely that missingness in the outcome depended on its true value?	N
	**Risk of bias judgement**	Low
Bias in measurement of the outcome	4.1 Was the method of measuring the outcome inappropriate?	N
	4.2 Could measurement or ascertainment of the outcome have differed between intervention groups?	NA
	4.3 Were outcome assessors aware of the intervention received by study participants?	N
	4.4 If Y/PY/NI to 4.3: Could assessment of the outcome have been influenced by knowledge of intervention received?	PY
	4.5 If Y/PY/NI to 4.4: Is it likely that assessment of the outcome was influenced by knowledge of intervention received?	NA
	**Risk of bias judgement**	Low
Bias in selection of the reported result	5.1 Were the data that produced this result analysed in accordance with a pre-specified analysis plan that was finalized before unblinded outcome data were	Y
	5.2 ... multiple eligible outcome measurements (e.g. scales, definitions, time points) within the outcome domain?	Y
	5.3 ... multiple eligible analyses of the data?	NA
	**Risk of bias judgement**	Some concerns
Overall bias	**Risk of bias judgement**	Some concerns
Study	Jiangfei(2014)	
Domain	Signalling question	Responese
Bias arising from the randomization process	1.1 Was the allocation sequence random?	Y
	1.2 Was the allocation sequence concealed until participants were enrolled and assigned to interventions?	NA
	1.3 Did baseline differences between intervention groups suggest a problem with the randomization process?	NA
	**Risk of bias judgement**	Some concerns
Bias due to deviations	2.1.Were participants aware of their assigned intervention during the trial?	N
	2.2.Were carers and people delivering the interventions aware of participants' assigned intervention during the trial?	NA
	2.3. If Y/PY/NI to 2.1 or 2.2: Were there deviations from the intended intervention that arose because of the experimental context?	NI
	2.4 If Y/PY to 2.3: Were these deviations likely to have affected the outcome?	PY

deviations from intended interventions	2.5. If Y/PY/NI to 2.4: Were these deviations from intended intervention balanced between groups?	NA
	2.6 Was an appropriate analysis used to estimate the effect of assignment to intervention?	NI
	2.7 If N/PN/NI to 2.6: Was there potential for a substantial impact (on the result) of the failure to analyse participants in the group to which they were randomized?	NI
	**Risk of bias judgement**	Some concerns
Bias due to missing outcome data	3.1 Were data for this outcome available for all, or nearly all, participants randomized?	Y
	3.2 If N/PN/NI to 3.1: Is there evidence that result was not biased by missing outcome data?	PN
	3.3 If N/PN to 3.2: Could missingness in the outcome depend on its true value?	NA
	3.4 If Y/PY/NI to 3.3: Is it likely that missingness in the outcome depended on its true value?	N
	**Risk of bias judgement**	Low
Bias in measurement of the outcome	4.1 Was the method of measuring the outcome inappropriate?	N
	4.2 Could measurement or ascertainment of the outcome have differed between intervention groups?	NA
	4.3 Were outcome assessors aware of the intervention received by study participants?	N
	4.4 If Y/PY/NI to 4.3: Could assessment of the outcome have been influenced by knowledge of intervention received?	PY
	4.5 If Y/PY/NI to 4.4: Is it likely that assessment of the outcome was influenced by knowledge of intervention received?	NA
	**Risk of bias judgement**	Low
Bias in selection of the reported result	5.1 Were the data that produced this result analysed in accordance with a pre-specified analysis plan that was finalized before unblinded outcome data were	Y
	5.2 ... multiple eligible outcome measurements (e.g. scales, definitions, time points) within the outcome domain?	Y
	5.3 ... multiple eligible analyses of the data?	NA
	**Risk of bias judgement**	Some concerns
Overall bias	**Risk of bias judgement**	Some concerns
Study	Maolinlin(2021)	
Domain	Signalling question	Responese
Bias arising from the randomization process	1.1 Was the allocation sequence random?	Y
	1.2 Was the allocation sequence concealed until participants were enrolled and assigned to interventions?	NA
	1.3 Did baseline differences between intervention groups suggest a problem with the randomization process?	NA
	**Risk of bias judgement**	Some concerns
Bias due to deviations from intended interventions	2.1.Were participants aware of their assigned intervention during the trial?	Y
	2.2.Were carers and people delivering the interventions aware of participants' assigned intervention during the trial?	Y
	2.3. If Y/PY/NI to 2.1 or 2.2: Were there deviations from the intended intervention that arose because of the experimental context?	NI
	2.4 If Y/PY to 2.3: Were these deviations likely to have affected the outcome?	PY
	2.5. If Y/PY/NI to 2.4: Were these deviations from intended intervention balanced between groups?	NA
	2.6 Was an appropriate analysis used to estimate the effect of assignment to intervention?	Y
	2.7 If N/PN/NI to 2.6: Was there potential for a substantial impact (on the result) of the failure to analyse participants in the group to which they were randomized?	NI
	**Risk of bias judgement**	Low
Bias due to missing outcome data	3.1 Were data for this outcome available for all, or nearly all, participants randomized?	Y
	3.2 If N/PN/NI to 3.1: Is there evidence that result was not biased by missing outcome data?	PN
	3.3 If N/PN to 3.2: Could missingness in the outcome depend on its true value?	NA
	3.4 If Y/PY/NI to 3.3: Is it likely that missingness in the outcome depended on its true value?	N
	**Risk of bias judgement**	Low
	4.1 Was the method of measuring the outcome inappropriate?	N

Bias in measurement of the outcome	4.2 Could measurement or ascertainment of the outcome have differed between intervention groups?	NA
	4.3 Were outcome assessors aware of the intervention received by study participants?	N
	4.4 If Y/PY/NI to 4.3: Could assessment of the outcome have been influenced by knowledge of intervention received?	PY
	4.5 If Y/PY/NI to 4.4: Is it likely that assessment of the outcome was influenced by knowledge of intervention received?	NA
	**Risk of bias judgement**	Low
Bias in selection of the reported result	5.1 Were the data that produced this result analysed in accordance with a pre-specified analysis plan that was finalized before unblinded outcome data were	Y
	5.2 ... multiple eligible outcome measurements (e.g. scales, definitions, time points) within the outcome domain?	Y
	5.3 ... multiple eligible analyses of the data?	NA
	**Risk of bias judgement**	Some concerns
Overall bias	**Risk of bias judgement**	Some concerns
Study	Dinglihong(2014)	
Domain	Signalling question	Responese
Bias arising from the randomization process	1.1 Was the allocation sequence random?	Y
	1.2 Was the allocation sequence concealed until participants were enrolled and assigned to interventions?	NA
	1.3 Did baseline differences between intervention groups suggest a problem with the randomization process?	NA
	**Risk of bias judgement**	Some concerns
Bias due to deviations from intended interventions	2.1.Were participants aware of their assigned intervention during the trial?	N
	2.2.Were carers and people delivering the interventions aware of participants' assigned intervention during the trial?	NA
	2.3. If Y/PY/NI to 2.1 or 2.2: Were there deviations from the intended intervention that arose because of the experimental context?	NI
	2.4 If Y/PY to 2.3: Were these deviations likely to have affected the outcome?	PY
	2.5. If Y/PY/NI to 2.4: Were these deviations from intended intervention balanced between groups?	N
	2.6 Was an appropriate analysis used to estimate the effect of assignment to intervention?	NI
	2.7 If N/PN/NI to 2.6: Was there potential for a substantial impact (on the result) of the failure to analyse participants in the group to which they were randomized?	NI
	**Risk of bias judgement**	High
Bias due to missing outcome data	3.1 Were data for this outcome available for all, or nearly all, participants randomized?	Y
	3.2 If N/PN/NI to 3.1: Is there evidence that result was not biased by missing outcome data?	PN
	3.3 If N/PN to 3.2: Could missingness in the outcome depend on its true value?	NA
	3.4 If Y/PY/NI to 3.3: Is it likely that missingness in the outcome depended on its true value?	N
	**Risk of bias judgement**	Low
Bias in measurement of the outcome	4.1 Was the method of measuring the outcome inappropriate?	N
	4.2 Could measurement or ascertainment of the outcome have differed between intervention groups?	NA
	4.3 Were outcome assessors aware of the intervention received by study participants?	N
	4.4 If Y/PY/NI to 4.3: Could assessment of the outcome have been influenced by knowledge of intervention received?	PY
	4.5 If Y/PY/NI to 4.4: Is it likely that assessment of the outcome was influenced by knowledge of intervention received?	NA
	**Risk of bias judgement**	Low
Bias in selection of the reported result	5.1 Were the data that produced this result analysed in accordance with a pre-specified analysis plan that was finalized before unblinded outcome data were	Y
	5.2 ... multiple eligible outcome measurements (e.g. scales, definitions, time points) within the outcome domain?	Y
	5.3 ... multiple eligible analyses of the data?	NA
	**Risk of bias judgement**	Some concerns

Overall bias	**Risk of bias judgement**	Some concerns
Study	Panjie(2017)	
Domain	Signalling question	Responese
Bias arising from the randomization process	1.1 Was the allocation sequence random?	Y
	1.2 Was the allocation sequence concealed until participants were enrolled and assigned to interventions?	NA
	1.3 Did baseline differences between intervention groups suggest a problem with the randomization process?	NA
	**Risk of bias judgement**	Some concerns
Bias due to deviations from intended interventions	2.1.Were participants aware of their assigned intervention during the trial?	Y
	2.2.Were carers and people delivering the interventions aware of participants' assigned intervention during the trial?	Y
	2.3. If Y/PY/NI to 2.1 or 2.2: Were there deviations from the intended intervention that arose because of the experimental context?	Y
	2.4 If Y/PY to 2.3: Were these deviations likely to have affected the outcome?	PY
	2.5. If Y/PY/NI to 2.4: Were these deviations from intended intervention balanced between groups?	NA
	2.6 Was an appropriate analysis used to estimate the effect of assignment to intervention?	NA
	2.7 If N/PN/NI to 2.6: Was there potential for a substantial impact (on the result) of the failure to analyse participants in the group to which they were randomized?	NI
	**Risk of bias judgement**	Low
Bias due to missing outcome data	3.1 Were data for this outcome available for all, or nearly all, participants randomized?	Y
	3.2 If N/PN/NI to 3.1: Is there evidence that result was not biased by missing outcome data?	PN
	3.3 If N/PN to 3.2: Could missingness in the outcome depend on its true value?	NA
	3.4 If Y/PY/NI to 3.3: Is it likely that missingness in the outcome depended on its true value?	N
	**Risk of bias judgement**	Low
Bias in measurement of the outcome	4.1 Was the method of measuring the outcome inappropriate?	N
	4.2 Could measurement or ascertainment of the outcome have differed between intervention groups?	NA
	4.3 Were outcome assessors aware of the intervention received by study participants?	N
	4.4 If Y/PY/NI to 4.3: Could assessment of the outcome have been influenced by knowledge of intervention received?	PY
	4.5 If Y/PY/NI to 4.4: Is it likely that assessment of the outcome was influenced by knowledge of intervention received?	NA
	**Risk of bias judgement**	Low
Bias in selection of the reported result	5.1 Were the data that produced this result analysed in accordance with a pre-specified analysis plan that was finalized before unblinded outcome data were	Y
	5.2 ... multiple eligible outcome measurements (e.g. scales, definitions, time points) within the outcome domain?	Y
	5.3 ... multiple eligible analyses of the data?	NA
	**Risk of bias judgement**	Some concerns
Overall bias	**Risk of bias judgement**	Some concerns
Study	Liuji(2016)	
Domain	Signalling question	Responese
Bias arising from the randomization process	1.1 Was the allocation sequence random?	Y
	1.2 Was the allocation sequence concealed until participants were enrolled and assigned to interventions?	NA
	1.3 Did baseline differences between intervention groups suggest a problem with the randomization process?	NA
	**Risk of bias judgement**	Some concerns

Bias due to deviations from intended interventions	2.1.Were participants aware of their assigned intervention during the trial?	Y
	2.2.Were carers and people delivering the interventions aware of participants' assigned intervention during the trial?	Y
	2.3. If Y/PY/NI to 2.1 or 2.2: Were there deviations from the intended intervention that arose because of the experimental context?	NI
	2.4 If Y/PY to 2.3: Were these deviations likely to have affected the outcome?	PY
	2.5. If Y/PY/NI to 2.4: Were these deviations from intended intervention balanced between groups?	NA
	2.6 Was an appropriate analysis used to estimate the effect of assignment to intervention?	Y
	2.7 If N/PN/NI to 2.6: Was there potential for a substantial impact (on the result) of the failure to analyse participants in the group to which they were randomized?	NI
	**Risk of bias judgement**	Low
Bias due to missing outcome data	3.1 Were data for this outcome available for all, or nearly all, participants randomized?	Y
	3.2 If N/PN/NI to 3.1: Is there evidence that result was not biased by missing outcome data?	PN
	3.3 If N/PN to 3.2: Could missingness in the outcome depend on its true value?	NA
	3.4 If Y/PY/NI to 3.3: Is it likely that missingness in the outcome depended on its true value?	N
	**Risk of bias judgement**	Low
Bias in measurement of the outcome	4.1 Was the method of measuring the outcome inappropriate?	N
	4.2 Could measurement or ascertainment of the outcome have differed between intervention groups?	NA
	4.3 Were outcome assessors aware of the intervention received by study participants?	N
	4.4 If Y/PY/NI to 4.3: Could assessment of the outcome have been influenced by knowledge of intervention received?	PY
	4.5 If Y/PY/NI to 4.4: Is it likely that assessment of the outcome was influenced by knowledge of intervention received?	NA
	**Risk of bias judgement**	Low
Bias in selection of the reported result	5.1 Were the data that produced this result analysed in accordance with a pre-specified analysis plan that was finalized before unblinded outcome data were	Y
	5.2 ... multiple eligible outcome measurements (e.g. scales, definitions, time points) within the outcome domain?	Y
	5.3 ... multiple eligible analyses of the data?	NA
	**Risk of bias judgement**	Some concerns
Overall bias	**Risk of bias judgement**	Some concerns
Study	Liujiarong(2016)	
Domain	Signalling question	Responese
Bias arising from the randomization process	1.1 Was the allocation sequence random?	Y
	1.2 Was the allocation sequence concealed until participants were enrolled and assigned to interventions?	NA
	1.3 Did baseline differences between intervention groups suggest a problem with the randomization process?	NA
	**Risk of bias judgement**	Some concerns
Bias due to deviations from intended interventions	2.1.Were participants aware of their assigned intervention during the trial?	Y
	2.2.Were carers and people delivering the interventions aware of participants' assigned intervention during the trial?	Y
	2.3. If Y/PY/NI to 2.1 or 2.2: Were there deviations from the intended intervention that arose because of the experimental context?	PY
	2.4 If Y/PY to 2.3: Were these deviations likely to have affected the outcome?	Y
	2.5. If Y/PY/NI to 2.4: Were these deviations from intended intervention balanced between groups?	Y
	2.6 Was an appropriate analysis used to estimate the effect of assignment to intervention?	NI
	2.7 If N/PN/NI to 2.6: Was there potential for a substantial impact (on the result) of the failure to analyse participants in the group to which they were randomized?	NI
	**Risk of bias judgement**	Low
Bias due to missing	3.1 Were data for this outcome available for all, or nearly all, participants randomized?	Y
	3.2 If N/PN/NI to 3.1: Is there evidence that result was not biased by missing outcome data?	PN
	3.3 If N/PN to 3.2: Could missingness in the outcome depend on its true value?	NA

outcome data	3.4 If Y/PY/NI to 3.3: Is it likely that missingness in the outcome depended on its true value?	N
	**Risk of bias judgement**	Low
Bias in measurement of the outcome	4.1 Was the method of measuring the outcome inappropriate?	N
	4.2 Could measurement or ascertainment of the outcome have differed between intervention groups?	NA
	4.3 Were outcome assessors aware of the intervention received by study participants?	N
	4.4 If Y/PY/NI to 4.3: Could assessment of the outcome have been influenced by knowledge of intervention received?	PY
	4.5 If Y/PY/NI to 4.4: Is it likely that assessment of the outcome was influenced by knowledge of intervention received?	NA
	**Risk of bias judgement**	Low
Bias in selection of the reported result	5.1 Were the data that produced this result analysed in accordance with a pre-specified analysis plan that was finalized before unblinded outcome data were	Y
	5.2 ... multiple eligible outcome measurements (e.g. scales, definitions, time points) within the outcome domain?	Y
	5.3 ... multiple eligible analyses of the data?	NA
	**Risk of bias judgement**	Some concerns
Overall bias	**Risk of bias judgement**	Some concerns
Study	Moguipei(2016)	
Domain	Signalling question	Responese
Bias arising from the randomization process	1.1 Was the allocation sequence random?	Y
	1.2 Was the allocation sequence concealed until participants were enrolled and assigned to interventions?	NA
	1.3 Did baseline differences between intervention groups suggest a problem with the randomization process?	NA
	**Risk of bias judgement**	Some concerns
Bias due to deviations from intended interventions	2.1.Were participants aware of their assigned intervention during the trial?	N
	2.2.Were carers and people delivering the interventions aware of participants' assigned intervention during the trial?	NA
	2.3. If Y/PY/NI to 2.1 or 2.2: Were there deviations from the intended intervention that arose because of the experimental context?	NI
	2.4 If Y/PY to 2.3: Were these deviations likely to have affected the outcome?	PY
	2.5. If Y/PY/NI to 2.4: Were these deviations from intended intervention balanced between groups?	NA
	2.6 Was an appropriate analysis used to estimate the effect of assignment to intervention?	NI
	2.7 If N/PN/NI to 2.6: Was there potential for a substantial impact (on the result) of the failure to analyse participants in the group to which they were randomized?	NI
	**Risk of bias judgement**	Some concerns
Bias due to missing outcome data	3.1 Were data for this outcome available for all, or nearly all, participants randomized?	Y
	3.2 If N/PN/NI to 3.1: Is there evidence that result was not biased by missing outcome data?	PN
	3.3 If N/PN to 3.2: Could missingness in the outcome depend on its true value?	NA
	3.4 If Y/PY/NI to 3.3: Is it likely that missingness in the outcome depended on its true value?	N
	**Risk of bias judgement**	Some concerns
Bias in measurement of the outcome	4.1 Was the method of measuring the outcome inappropriate?	N
	4.2 Could measurement or ascertainment of the outcome have differed between intervention groups?	NA
	4.3 Were outcome assessors aware of the intervention received by study participants?	N
	4.4 If Y/PY/NI to 4.3: Could assessment of the outcome have been influenced by knowledge of intervention received?	PY
	4.5 If Y/PY/NI to 4.4: Is it likely that assessment of the outcome was influenced by knowledge of intervention received?	NA
	**Risk of bias judgement**	Low
Bias in selection	5.1 Were the data that produced this result analysed in accordance with a pre-specified analysis plan that was finalized before unblinded outcome data were	Y

Bias in selection of the reported result	5.2 ... multiple eligible outcome measurements (e.g. scales, definitions, time points) within the outcome domain?	Y
	5.3 ... multiple eligible analyses of the data?	NA
	**Risk of bias judgement**	Some concerns
Overall bias	**Risk of bias judgement**	Some concerns
Study	Wulin(2017)	
Domain	Signalling question	Responese
Bias arising from the randomization process	1.1 Was the allocation sequence random?	Y
	1.2 Was the allocation sequence concealed until participants were enrolled and assigned to interventions?	NA
	1.3 Did baseline differences between intervention groups suggest a problem with the randomization process?	NA
	**Risk of bias judgement**	Some concerns
Bias due to deviations from intended interventions	2.1.Were participants aware of their assigned intervention during the trial?	N
	2.2.Were carers and people delivering the interventions aware of participants' assigned intervention during the trial?	NA
	2.3. If Y/PY/NI to 2.1 or 2.2: Were there deviations from the intended intervention that arose because of the experimental context?	NI
	2.4 If Y/PY to 2.3: Were these deviations likely to have affected the outcome?	PY
	2.5. If Y/PY/NI to 2.4: Were these deviations from intended intervention balanced between groups?	NA
	2.6 Was an appropriate analysis used to estimate the effect of assignment to intervention?	NI
	2.7 If N/PN/NI to 2.6: Was there potential for a substantial impact (on the result) of the failure to analyse participants in the group to which they were randomized?	NI
	**Risk of bias judgement**	Some concerns
Bias due to missing outcome data	3.1 Were data for this outcome available for all, or nearly all, participants randomized?	Y
	3.2 If N/PN/NI to 3.1: Is there evidence that result was not biased by missing outcome data?	PN
	3.3 If N/PN to 3.2: Could missingness in the outcome depend on its true value?	NA
	3.4 If Y/PY/NI to 3.3: Is it likely that missingness in the outcome depended on its true value?	N
	**Risk of bias judgement**	Low
Bias in measurement of the outcome	4.1 Was the method of measuring the outcome inappropriate?	N
	4.2 Could measurement or ascertainment of the outcome have differed between intervention groups?	NA
	4.3 Were outcome assessors aware of the intervention received by study participants?	N
	4.4 If Y/PY/NI to 4.3: Could assessment of the outcome have been influenced by knowledge of intervention received?	PY
	4.5 If Y/PY/NI to 4.4: Is it likely that assessment of the outcome was influenced by knowledge of intervention received?	NA
	**Risk of bias judgement**	Low
Bias in selection of the reported result	5.1 Were the data that produced this result analysed in accordance with a pre-specified analysis plan that was finalized before unblinded outcome data were	Y
	5.2 ... multiple eligible outcome measurements (e.g. scales, definitions, time points) within the outcome domain?	Y
	5.3 ... multiple eligible analyses of the data?	NA
	**Risk of bias judgement**	Some concerns
Overall bias	**Risk of bias judgement**	Some concerns
Study	Chenhaijun(2015)	
Domain	Signalling question	Responese

Bias arising from the randomization process	1.1 Was the allocation sequence random?	Y
	1.2 Was the allocation sequence concealed until participants were enrolled and assigned to interventions?	NA
	1.3 Did baseline differences between intervention groups suggest a problem with the randomization process?	NA
	**Risk of bias judgement**	Some concerns
Bias due to deviations from intended interventions	2.1.Were participants aware of their assigned intervention during the trial?	Y
	2.2.Were carers and people delivering the interventions aware of participants' assigned intervention during the trial?	Y
	2.3. If Y/PY/NI to 2.1 or 2.2: Were there deviations from the intended intervention that arose because of the experimental context?	Y
	2.4 If Y/PY to 2.3: Were these deviations likely to have affected the outcome?	NI
	2.5. If Y/PY/NI to 2.4: Were these deviations from intended intervention balanced between groups?	PY
	2.6 Was an appropriate analysis used to estimate the effect of assignment to intervention?	N
	2.7 If N/PN/NI to 2.6: Was there potential for a substantial impact (on the result) of the failure to analyse participants in the group to which they were randomized?	NI
	**Risk of bias judgement**	Low
Bias due to missing outcome data	3.1 Were data for this outcome available for all, or nearly all, participants randomized?	Y
	3.2 If N/PN/NI to 3.1: Is there evidence that result was not biased by missing outcome data?	NI
	3.3 If N/PN to 3.2: Could missingness in the outcome depend on its true value?	NI
	3.4 If Y/PY/NI to 3.3: Is it likely that missingness in the outcome depended on its true value?	N
	**Risk of bias judgement**	Some concerns
Bias in measurement of the outcome	4.1 Was the method of measuring the outcome inappropriate?	N
	4.2 Could measurement or ascertainment of the outcome have differed between intervention groups?	NA
	4.3 Were outcome assessors aware of the intervention received by study participants?	N
	4.4 If Y/PY/NI to 4.3: Could assessment of the outcome have been influenced by knowledge of intervention received?	PY
	4.5 If Y/PY/NI to 4.4: Is it likely that assessment of the outcome was influenced by knowledge of intervention received?	NA
	**Risk of bias judgement**	Low
Bias in selection of the reported result	5.1 Were the data that produced this result analysed in accordance with a pre-specified analysis plan that was finalized before unblinded outcome data were	Y
	5.2 ... multiple eligible outcome measurements (e.g. scales, definitions, time points) within the outcome domain?	Y
	5.3 ... multiple eligible analyses of the data?	NA
	**Risk of bias judgement**	Some concerns
Overall bias	**Risk of bias judgement**	Some concerns
Study	Shenguijun(2017)	
Domain	Signalling question	Responese
Bias arising from the randomization process	1.1 Was the allocation sequence random?	Y
	1.2 Was the allocation sequence concealed until participants were enrolled and assigned to interventions?	NA
	1.3 Did baseline differences between intervention groups suggest a problem with the randomization process?	NA
	**Risk of bias judgement**	Some concerns
Bias due to deviations from intended	2.1.Were participants aware of their assigned intervention during the trial?	N
	2.2.Were carers and people delivering the interventions aware of participants' assigned intervention during the trial?	NA
	2.3. If Y/PY/NI to 2.1 or 2.2: Were there deviations from the intended intervention that arose because of the experimental context?	NI
	2.4 If Y/PY to 2.3: Were these deviations likely to have affected the outcome?	PY
	2.5. If Y/PY/NI to 2.4: Were these deviations from intended intervention balanced between groups?	NA

from intended interventions	2.6 Was an appropriate analysis used to estimate the effect of assignment to intervention?	NI
	2.7 If N/PN/NI to 2.6: Was there potential for a substantial impact (on the result) of the failure to analyse participants in the group to which they were randomized?	NI
	**Risk of bias judgement**	Some concerns
Bias due to missing outcome data	3.1 Were data for this outcome available for all, or nearly all, participants randomized?	Y
	3.2 If N/PN/NI to 3.1: Is there evidence that result was not biased by missing outcome data?	PN
	3.3 If N/PN to 3.2: Could missingness in the outcome depend on its true value?	NA
	3.4 If Y/PY/NI to 3.3: Is it likely that missingness in the outcome depended on its true value?	N
	**Risk of bias judgement**	Low
Bias in measurement of the outcome	4.1 Was the method of measuring the outcome inappropriate?	N
	4.2 Could measurement or ascertainment of the outcome have differed between intervention groups?	NA
	4.3 Were outcome assessors aware of the intervention received by study participants?	N
	4.4 If Y/PY/NI to 4.3: Could assessment of the outcome have been influenced by knowledge of intervention received?	PY
	4.5 If Y/PY/NI to 4.4: Is it likely that assessment of the outcome was influenced by knowledge of intervention received?	NA
	**Risk of bias judgement**	Low
Bias in selection of the reported result	5.1 Were the data that produced this result analysed in accordance with a pre-specified analysis plan that was finalized before unblinded outcome data were	Y
	5.2 ... multiple eligible outcome measurements (e.g. scales, definitions, time points) within the outcome domain?	Y
	5.3 ... multiple eligible analyses of the data?	NA
	**Risk of bias judgement**	Some concerns
Overall bias	**Risk of bias judgement**	Some concerns
Study	Qinyan(2016)	
Domain	Signalling question	Responese
Bias arising from the randomization process	1.1 Was the allocation sequence random?	Y
	1.2 Was the allocation sequence concealed until participants were enrolled and assigned to interventions?	NA
	1.3 Did baseline differences between intervention groups suggest a problem with the randomization process?	NA
	**Risk of bias judgement**	Some concerns
Bias due to deviations from intended interventions	2.1.Were participants aware of their assigned intervention during the trial?	Y
	2.2.Were carers and people delivering the interventions aware of participants' assigned intervention during the trial?	Y
	2.3. If Y/PY/NI to 2.1 or 2.2: Were there deviations from the intended intervention that arose because of the experimental context?	NI
	2.4 If Y/PY to 2.3: Were these deviations likely to have affected the outcome?	PY
	2.5. If Y/PY/NI to 2.4: Were these deviations from intended intervention balanced between groups?	Y
	2.6 Was an appropriate analysis used to estimate the effect of assignment to intervention?	NI
	2.7 If N/PN/NI to 2.6: Was there potential for a substantial impact (on the result) of the failure to analyse participants in the group to which they were randomized?	NI
	**Risk of bias judgement**	Low
Bias due to missing outcome data	3.1 Were data for this outcome available for all, or nearly all, participants randomized?	Y
	3.2 If N/PN/NI to 3.1: Is there evidence that result was not biased by missing outcome data?	PN
	3.3 If N/PN to 3.2: Could missingness in the outcome depend on its true value?	NA
	3.4 If Y/PY/NI to 3.3: Is it likely that missingness in the outcome depended on its true value?	N
	**Risk of bias judgement**	Low
Bias in	4.1 Was the method of measuring the outcome inappropriate?	N
	4.2 Could measurement or ascertainment of the outcome have differed between intervention groups?	NA

[illegible] measurement of the outcome	4.3 Were outcome assessors aware of the intervention received by study participants?	N
	4.4 If Y/PY/NI to 4.3: Could assessment of the outcome have been influenced by knowledge of intervention received?	PY
	4.5 If Y/PY/NI to 4.4: Is it likely that assessment of the outcome was influenced by knowledge of intervention received?	NA
	**Risk of bias judgement**	Some concerns
Bias in selection of the reported result	5.1 Were the data that produced this result analysed in accordance with a pre-specified analysis plan that was finalized before unblinded outcome data were	Y
	5.2 ... multiple eligible outcome measurements (e.g. scales, definitions, time points) within the outcome domain?	Y
	5.3 ... multiple eligible analyses of the data?	NA
	**Risk of bias judgement**	Some concerns
Overall bias	**Risk of bias judgement**	Some concerns
Study	Wangyan(2015)	
Domain	Signalling question	Responese
Bias arising from the randomization process	1.1 Was the allocation sequence random?	Y
	1.2 Was the allocation sequence concealed until participants were enrolled and assigned to interventions?	NA
	1.3 Did baseline differences between intervention groups suggest a problem with the randomization process?	NA
	**Risk of bias judgement**	Some concerns
Bias due to deviations from intended interventions	2.1.Were participants aware of their assigned intervention during the trial?	Y
	2.2.Were carers and people delivering the interventions aware of participants' assigned intervention during the trial?	Y
	2.3. If Y/PY/NI to 2.1 or 2.2: Were there deviations from the intended intervention that arose because of the experimental context?	NI
	2.4 If Y/PY to 2.3: Were these deviations likely to have affected the outcome?	PY
	2.5. If Y/PY/NI to 2.4: Were these deviations from intended intervention balanced between groups?	NA
	2.6 Was an appropriate analysis used to estimate the effect of assignment to intervention?	NI
	2.7 If N/PN/NI to 2.6: Was there potential for a substantial impact (on the result) of the failure to analyse participants in the group to which they were randomized?	NI
	**Risk of bias judgement**	Low
Bias due to missing outcome data	3.1 Were data for this outcome available for all, or nearly all, participants randomized?	Y
	3.2 If N/PN/NI to 3.1: Is there evidence that result was not biased by missing outcome data?	PN
	3.3 If N/PN to 3.2: Could missingness in the outcome depend on its true value?	NA
	3.4 If Y/PY/NI to 3.3: Is it likely that missingness in the outcome depended on its true value?	N
	**Risk of bias judgement**	Low
Bias in measurement of the outcome	4.1 Was the method of measuring the outcome inappropriate?	N
	4.2 Could measurement or ascertainment of the outcome have differed between intervention groups?	NA
	4.3 Were outcome assessors aware of the intervention received by study participants?	N
	4.4 If Y/PY/NI to 4.3: Could assessment of the outcome have been influenced by knowledge of intervention received?	PY
	4.5 If Y/PY/NI to 4.4: Is it likely that assessment of the outcome was influenced by knowledge of intervention received?	NA
	**Risk of bias judgement**	Some concerns
Bias in selection of the reported result	5.1 Were the data that produced this result analysed in accordance with a pre-specified analysis plan that was finalized before unblinded outcome data were	Y
	5.2 ... multiple eligible outcome measurements (e.g. scales, definitions, time points) within the outcome domain?	Y
	5.3 ... multiple eligible analyses of the data?	NA
	**Risk of bias judgement**	Low

Overall bias	**Risk of bias judgement**	Some concerns
Study	Zhulili(2016)	
Domain	Signalling question	Responese
Bias arising from the randomization process	1.1 Was the allocation sequence random?	Y
	1.2 Was the allocation sequence concealed until participants were enrolled and assigned to interventions?	NA
	1.3 Did baseline differences between intervention groups suggest a problem with the randomization process?	NA
	**Risk of bias judgement**	Some concerns
Bias due to deviations from intended interventions	2.1.Were participants aware of their assigned intervention during the trial?	N
	2.2.Were carers and people delivering the interventions aware of participants' assigned intervention during the trial?	N
	2.3. If Y/PY/NI to 2.1 or 2.2: Were there deviations from the intended intervention that arose because of the experimental context?	NI
	2.4 If Y/PY to 2.3: Were these deviations likely to have affected the outcome?	PY
	2.5. If Y/PY/NI to 2.4: Were these deviations from intended intervention balanced between groups?	NI
	2.6 Was an appropriate analysis used to estimate the effect of assignment to intervention?	N
	2.7 If N/PN/NI to 2.6: Was there potential for a substantial impact (on the result) of the failure to analyse participants in the group to which they were randomized?	NI
	**Risk of bias judgement**	High
Bias due to missing outcome data	3.1 Were data for this outcome available for all, or nearly all, participants randomized?	Y
	3.2 If N/PN/NI to 3.1: Is there evidence that result was not biased by missing outcome data?	PN
	3.3 If N/PN to 3.2: Could missingness in the outcome depend on its true value?	NA
	3.4 If Y/PY/NI to 3.3: Is it likely that missingness in the outcome depended on its true value?	N
	**Risk of bias judgement**	Low
Bias in measurement of the outcome	4.1 Was the method of measuring the outcome inappropriate?	N
	4.2 Could measurement or ascertainment of the outcome have differed between intervention groups?	NA
	4.3 Were outcome assessors aware of the intervention received by study participants?	N
	4.4 If Y/PY/NI to 4.3: Could assessment of the outcome have been influenced by knowledge of intervention received?	PY
	4.5 If Y/PY/NI to 4.4: Is it likely that assessment of the outcome was influenced by knowledge of intervention received?	NA
	**Risk of bias judgement**	Low
Bias in selection of the reported result	5.1 Were the data that produced this result analysed in accordance with a pre-specified analysis plan that was finalized before unblinded outcome data were	Y
	5.2 ... multiple eligible outcome measurements (e.g. scales, definitions, time points) within the outcome domain?	Y
	5.3 ... multiple eligible analyses of the data?	NA
	**Risk of bias judgement**	Some concerns
Overall bias	**Risk of bias judgement**	Some concerns
Study	Liuguifang(2015)	
Domain	Signalling question	Responese
Bias arising from the randomization process	1.1 Was the allocation sequence random?	Y
	1.2 Was the allocation sequence concealed until participants were enrolled and assigned to interventions?	NA
	1.3 Did baseline differences between intervention groups suggest a problem with the randomization process?	NA
	**Risk of bias judgement**	Some concerns

Bias due to deviations from intended interventions	2.1.Were participants aware of their assigned intervention during the trial?	Y
	2.2.Were carers and people delivering the interventions aware of participants' assigned intervention during the trial?	NA
	2.3. If Y/PY/NI to 2.1 or 2.2: Were there deviations from the intended intervention that arose because of the experimental context?	NI
	2.4 If Y/PY to 2.3: Were these deviations likely to have affected the outcome?	PY
	2.5. If Y/PY/NI to 2.4: Were these deviations from intended intervention balanced between groups?	NA
	2.6 Was an appropriate analysis used to estimate the effect of assignment to intervention?	NI
	2.7 If N/PN/NI to 2.6: Was there potential for a substantial impact (on the result) of the failure to analyse participants in the group to which they were randomized?	NI
	**Risk of bias judgement**	Some concerns
Bias due to missing outcome data	3.1 Were data for this outcome available for all, or nearly all, participants randomized?	Y
	3.2 If N/PN/NI to 3.1: Is there evidence that result was not biased by missing outcome data?	PN
	3.3 If N/PN to 3.2: Could missingness in the outcome depend on its true value?	NA
	3.4 If Y/PY/NI to 3.3: Is it likely that missingness in the outcome depended on its true value?	N
	**Risk of bias judgement**	Low
Bias in measurement of the outcome	4.1 Was the method of measuring the outcome inappropriate?	N
	4.2 Could measurement or ascertainment of the outcome have differed between intervention groups?	NA
	4.3 Were outcome assessors aware of the intervention received by study participants?	N
	4.4 If Y/PY/NI to 4.3: Could assessment of the outcome have been influenced by knowledge of intervention received?	PY
	4.5 If Y/PY/NI to 4.4: Is it likely that assessment of the outcome was influenced by knowledge of intervention received?	NA
	**Risk of bias judgement**	Low
Bias in selection of the reported result	5.1 Were the data that produced this result analysed in accordance with a pre-specified analysis plan that was finalized before unblinded outcome data were	Y
	5.2 ... multiple eligible outcome measurements (e.g. scales, definitions, time points) within the outcome domain?	Y
	5.3 ... multiple eligible analyses of the data?	NA
	**Risk of bias judgement**	Some concerns
Overall bias	**Risk of bias judgement**	Some concerns
Study	Gongtianyin(2016)	
Domain	Signalling question	Responese
Bias arising from the randomization process	1.1 Was the allocation sequence random?	Y
	1.2 Was the allocation sequence concealed until participants were enrolled and assigned to interventions?	NA
	1.3 Did baseline differences between intervention groups suggest a problem with the randomization process?	NA
	**Risk of bias judgement**	Some concerns
Bias due to deviations from intended interventions	2.1.Were participants aware of their assigned intervention during the trial?	Y
	2.2.Were carers and people delivering the interventions aware of participants' assigned intervention during the trial?	NA
	2.3. If Y/PY/NI to 2.1 or 2.2: Were there deviations from the intended intervention that arose because of the experimental context?	NI
	2.4 If Y/PY to 2.3: Were these deviations likely to have affected the outcome?	PY
	2.5. If Y/PY/NI to 2.4: Were these deviations from intended intervention balanced between groups?	NA
	2.6 Was an appropriate analysis used to estimate the effect of assignment to intervention?	N
	2.7 If N/PN/NI to 2.6: Was there potential for a substantial impact (on the result) of the failure to analyse participants in the group to which they were randomized?	NI
	**Risk of bias judgement**	Low
Bias due to	3.1 Were data for this outcome available for all, or nearly all, participants randomized?	Y
	3.2 If N/PN/NI to 3.1: Is there evidence that result was not biased by missing outcome data?	PN

missing outcome data	3.3 If N/PN to 3.2: Could missingness in the outcome depend on its true value?	NA
	3.4 If Y/PY/NI to 3.3: Is it likely that missingness in the outcome depended on its true value?	N
	**Risk of bias judgement**	Low
Bias in measurement of the outcome	4.1 Was the method of measuring the outcome inappropriate?	N
	4.2 Could measurement or ascertainment of the outcome have differed between intervention groups?	NA
	4.3 Were outcome assessors aware of the intervention received by study participants?	N
	4.4 If Y/PY/NI to 4.3: Could assessment of the outcome have been influenced by knowledge of intervention received?	PY
	4.5 If Y/PY/NI to 4.4: Is it likely that assessment of the outcome was influenced by knowledge of intervention received?	NA
	**Risk of bias judgement**	Low
Bias in selection of the reported result	5.1 Were the data that produced this result analysed in accordance with a pre-specified analysis plan that was finalized before unblinded outcome data were	Y
	5.2 ... multiple eligible outcome measurements (e.g. scales, definitions, time points) within the outcome domain?	Y
	5.3 ... multiple eligible analyses of the data?	NA
	**Risk of bias judgement**	Some concerns
Overall bias	**Risk of bias judgement**	Some concerns
Study	Ganxiaohong(2014)	
Domain	Signalling question	Responese
Bias arising from the randomization process	1.1 Was the allocation sequence random?	Y
	1.2 Was the allocation sequence concealed until participants were enrolled and assigned to interventions?	NA
	1.3 Did baseline differences between intervention groups suggest a problem with the randomization process?	NA
	**Risk of bias judgement**	Some concerns
Bias due to deviations from intended interventions	2.1.Were participants aware of their assigned intervention during the trial?	Y
	2.2.Were carers and people delivering the interventions aware of participants' assigned intervention during the trial?	NI
	2.3. If Y/PY/NI to 2.1 or 2.2: Were there deviations from the intended intervention that arose because of the experimental context?	Y
	2.4 If Y/PY to 2.3: Were these deviations likely to have affected the outcome?	PY
	2.5. If Y/PY/NI to 2.4: Were these deviations from intended intervention balanced between groups?	Y
	2.6 Was an appropriate analysis used to estimate the effect of assignment to intervention?	NA
	2.7 If N/PN/NI to 2.6: Was there potential for a substantial impact (on the result) of the failure to analyse participants in the group to which they were randomized?	NA
	**Risk of bias judgement**	Low
Bias due to missing outcome data	3.1 Were data for this outcome available for all, or nearly all, participants randomized?	NA
	3.2 If N/PN/NI to 3.1: Is there evidence that result was not biased by missing outcome data?	PN
	3.3 If N/PN to 3.2: Could missingness in the outcome depend on its true value?	NA
	3.4 If Y/PY/NI to 3.3: Is it likely that missingness in the outcome depended on its true value?	N
	**Risk of bias judgement**	Some concerns
Bias in measurement of the outcome	4.1 Was the method of measuring the outcome inappropriate?	N
	4.2 Could measurement or ascertainment of the outcome have differed between intervention groups?	NA
	4.3 Were outcome assessors aware of the intervention received by study participants?	N
	4.4 If Y/PY/NI to 4.3: Could assessment of the outcome have been influenced by knowledge of intervention received?	PY
	4.5 If Y/PY/NI to 4.4: Is it likely that assessment of the outcome was influenced by knowledge of intervention received?	NA
	**Risk of bias judgement**	Low
Bias in selection	5.1 Were the data that produced this result analysed in accordance with a pre-specified analysis plan that was finalized before unblinded outcome data were	Y

Bias in selection of the reported result	5.2 ... multiple eligible outcome measurements (e.g. scales, definitions, time points) within the outcome domain?	Y
	5.3 ... multiple eligible analyses of the data?	NA
	**Risk of bias judgement**	Some concerns
Overall bias	**Risk of bias judgement**	Some concerns
Study	Hanchangming(2016)	
Domain	Signalling question	Responese
Bias arising from the randomization process	1.1 Was the allocation sequence random?	Y
	1.2 Was the allocation sequence concealed until participants were enrolled and assigned to interventions?	Y
	1.3 Did baseline differences between intervention groups suggest a problem with the randomization process?	NA
	**Risk of bias judgement**	Low
Bias due to deviations from intended interventions	2.1.Were participants aware of their assigned intervention during the trial?	NI
	2.2.Were carers and people delivering the interventions aware of participants' assigned intervention during the trial?	N
	2.3. If Y/PY/NI to 2.1 or 2.2: Were there deviations from the intended intervention that arose because of the experimental context?	NI
	2.4 If Y/PY to 2.3: Were these deviations likely to have affected the outcome?	PY
	2.5. If Y/PY/NI to 2.4: Were these deviations from intended intervention balanced between groups?	NI
	2.6 Was an appropriate analysis used to estimate the effect of assignment to intervention?	NI
	2.7 If N/PN/NI to 2.6: Was there potential for a substantial impact (on the result) of the failure to analyse participants in the group to which they were randomized?	NI
	**Risk of bias judgement**	High
Bias due to missing outcome data	3.1 Were data for this outcome available for all, or nearly all, participants randomized?	Y
	3.2 If N/PN/NI to 3.1: Is there evidence that result was not biased by missing outcome data?	PN
	3.3 If N/PN to 3.2: Could missingness in the outcome depend on its true value?	NA
	3.4 If Y/PY/NI to 3.3: Is it likely that missingness in the outcome depended on its true value?	N
	**Risk of bias judgement**	Low
Bias in measurement of the outcome	4.1 Was the method of measuring the outcome inappropriate?	N
	4.2 Could measurement or ascertainment of the outcome have differed between intervention groups?	N
	4.3 Were outcome assessors aware of the intervention received by study participants?	N
	4.4 If Y/PY/NI to 4.3: Could assessment of the outcome have been influenced by knowledge of intervention received?	PY
	4.5 If Y/PY/NI to 4.4: Is it likely that assessment of the outcome was influenced by knowledge of intervention received?	NA
	**Risk of bias judgement**	Low
Bias in selection of the reported result	5.1 Were the data that produced this result analysed in accordance with a pre-specified analysis plan that was finalized before unblinded outcome data were	Y
	5.2 ... multiple eligible outcome measurements (e.g. scales, definitions, time points) within the outcome domain?	Y
	5.3 ... multiple eligible analyses of the data?	NA
	**Risk of bias judgement**	Some concerns
Overall bias	**Risk of bias judgement**	Some concerns
Study	Zhangyuqin(2018)	
Domain	Signalling question	Responese
Bias arising from the	1.1 Was the allocation sequence random?	Y
	1.2 Was the allocation sequence concealed until participants were enrolled and assigned to interventions?	NA

the randomization process	1.3 Did baseline differences between intervention groups suggest a problem with the randomization process?	NA
	**Risk of bias judgement**	Some concerns
Bias due to deviations from intended interventions	2.1.Were participants aware of their assigned intervention during the trial?	Y
	2.2.Were carers and people delivering the interventions aware of participants' assigned intervention during the trial?	Y
	2.3. If Y/PY/NI to 2.1 or 2.2: Were there deviations from the intended intervention that arose because of the experimental context?	Y
	2.4 If Y/PY to 2.3: Were these deviations likely to have affected the outcome?	PY
	2.5. If Y/PY/NI to 2.4: Were these deviations from intended intervention balanced between groups?	NA
	2.6 Was an appropriate analysis used to estimate the effect of assignment to intervention?	NA
	2.7 If N/PN/NI to 2.6: Was there potential for a substantial impact (on the result) of the failure to analyse participants in the group to which they were randomized?	PN
	**Risk of bias judgement**	Low
Bias due to missing outcome data	3.1 Were data for this outcome available for all, or nearly all, participants randomized?	Y
	3.2 If N/PN/NI to 3.1: Is there evidence that result was not biased by missing outcome data?	PN
	3.3 If N/PN to 3.2: Could missingness in the outcome depend on its true value?	NA
	3.4 If Y/PY/NI to 3.3: Is it likely that missingness in the outcome depended on its true value?	Y
	**Risk of bias judgement**	Low
Bias in measurement of the outcome	4.1 Was the method of measuring the outcome inappropriate?	N
	4.2 Could measurement or ascertainment of the outcome have differed between intervention groups?	NA
	4.3 Were outcome assessors aware of the intervention received by study participants?	N
	4.4 If Y/PY/NI to 4.3: Could assessment of the outcome have been influenced by knowledge of intervention received?	PY
	4.5 If Y/PY/NI to 4.4: Is it likely that assessment of the outcome was influenced by knowledge of intervention received?	NA
	**Risk of bias judgement**	Low
Bias in selection of the reported result	5.1 Were the data that produced this result analysed in accordance with a pre-specified analysis plan that was finalized before unblinded outcome data were	Y
	5.2 ... multiple eligible outcome measurements (e.g. scales, definitions, time points) within the outcome domain?	Y
	5.3 ... multiple eligible analyses of the data?	NA
	**Risk of bias judgement**	Some concerns
Overall bias	**Risk of bias judgement**	Some concerns
Study	Yanghongying(2015)	
Domain	Signalling question	Responese
Bias arising from the randomization process	1.1 Was the allocation sequence random?	Y
	1.2 Was the allocation sequence concealed until participants were enrolled and assigned to interventions?	N
	1.3 Did baseline differences between intervention groups suggest a problem with the randomization process?	NA
	**Risk of bias judgement**	Some concerns
Bias due to deviations from intended interventions	2.1.Were participants aware of their assigned intervention during the trial?	Y
	2.2.Were carers and people delivering the interventions aware of participants' assigned intervention during the trial?	Y
	2.3. If Y/PY/NI to 2.1 or 2.2: Were there deviations from the intended intervention that arose because of the experimental context?	NA
	2.4 If Y/PY to 2.3: Were these deviations likely to have affected the outcome?	PY
	2.5. If Y/PY/NI to 2.4: Were these deviations from intended intervention balanced between groups?	NA
	2.6 Was an appropriate analysis used to estimate the effect of assignment to intervention?	NI
	2.7 If N/PN/NI to 2.6: Was there potential for a substantial impact (on the result) of the failure to analyse participants in the group to which they were randomized?	NI
	**Risk of bias judgement**	Low

Bias due to missing outcome data	3.1 Were data for this outcome available for all, or nearly all, participants randomized?	Y
	3.2 If N/PN/NI to 3.1: Is there evidence that result was not biased by missing outcome data?	NI
	3.3 If N/PN to 3.2: Could missingness in the outcome depend on its true value?	NA
	3.4 If Y/PY/NI to 3.3: Is it likely that missingness in the outcome depended on its true value?	N
	**Risk of bias judgement**	Some concerns
Bias in measurement of the outcome	4.1 Was the method of measuring the outcome inappropriate?	N
	4.2 Could measurement or ascertainment of the outcome have differed between intervention groups?	NA
	4.3 Were outcome assessors aware of the intervention received by study participants?	N
	4.4 If Y/PY/NI to 4.3: Could assessment of the outcome have been influenced by knowledge of intervention received?	NI
	4.5 If Y/PY/NI to 4.4: Is it likely that assessment of the outcome was influenced by knowledge of intervention received?	NA
	**Risk of bias judgement**	Some concerns
Bias in selection of the reported result	5.1 Were the data that produced this result analysed in accordance with a pre-specified analysis plan that was finalized before unblinded outcome data were	Y
	5.2 ... multiple eligible outcome measurements (e.g. scales, definitions, time points) within the outcome domain?	Y
	5.3 ... multiple eligible analyses of the data?	NA
	**Risk of bias judgement**	Some concerns
Overall bias	**Risk of bias judgement**	Some concerns
Study	Zhangjun(2015)	
Domain	Signalling question	Responese
Bias arising from the randomization process	1.1 Was the allocation sequence random?	Y
	1.2 Was the allocation sequence concealed until participants were enrolled and assigned to interventions?	NA
	1.3 Did baseline differences between intervention groups suggest a problem with the randomization process?	NA
	**Risk of bias judgement**	Some concerns
Bias due to deviations from intended interventions	2.1.Were participants aware of their assigned intervention during the trial?	N
	2.2.Were carers and people delivering the interventions aware of participants' assigned intervention during the trial?	NA
	2.3. If Y/PY/NI to 2.1 or 2.2: Were there deviations from the intended intervention that arose because of the experimental context?	NI
	2.4 If Y/PY to 2.3: Were these deviations likely to have affected the outcome?	PY
	2.5. If Y/PY/NI to 2.4: Were these deviations from intended intervention balanced between groups?	NA
	2.6 Was an appropriate analysis used to estimate the effect of assignment to intervention?	NI
	2.7 If N/PN/NI to 2.6: Was there potential for a substantial impact (on the result) of the failure to analyse participants in the group to which they were randomized?	NI
	**Risk of bias judgement**	Low
Bias due to missing outcome data	3.1 Were data for this outcome available for all, or nearly all, participants randomized?	Y
	3.2 If N/PN/NI to 3.1: Is there evidence that result was not biased by missing outcome data?	NI
	3.3 If N/PN to 3.2: Could missingness in the outcome depend on its true value?	NA
	3.4 If Y/PY/NI to 3.3: Is it likely that missingness in the outcome depended on its true value?	N
	**Risk of bias judgement**	Low
Bias in measurement [illegible]	4.1 Was the method of measuring the outcome inappropriate?	N
	4.2 Could measurement or ascertainment of the outcome have differed between intervention groups?	NA
	4.3 Were outcome assessors aware of the intervention received by study participants?	N
	4.4 If Y/PY/NI to 4.3: Could assessment of the outcome have been influenced by knowledge of intervention received?	NI

of the outcome	4.5 If Y/PY/NI to 4.4: Is it likely that assessment of the outcome was influenced by knowledge of intervention received?	NA
	**Risk of bias judgement**	Some concerns
Bias in selection of the reported result	5.1 Were the data that produced this result analysed in accordance with a pre-specified analysis plan that was finalized before unblinded outcome data were	Y
	5.2 ... multiple eligible outcome measurements (e.g. scales, definitions, time points) within the outcome domain?	Y
	5.3 ... multiple eligible analyses of the data?	NA
	**Risk of bias judgement**	Some concerns
Overall bias	**Risk of bias judgement**	Some concerns
Study	Zhumantang(2017)	
Domain	Signalling question	Responese
Bias arising from the randomization process	1.1 Was the allocation sequence random?	Y
	1.2 Was the allocation sequence concealed until participants were enrolled and assigned to interventions?	NA
	1.3 Did baseline differences between intervention groups suggest a problem with the randomization process?	NA
	**Risk of bias judgement**	Some concerns
Bias due to deviations from intended interventions	2.1.Were participants aware of their assigned intervention during the trial?	Y
	2.2.Were carers and people delivering the interventions aware of participants' assigned intervention during the trial?	Y
	2.3. If Y/PY/NI to 2.1 or 2.2: Were there deviations from the intended intervention that arose because of the experimental context?	NI
	2.4 If Y/PY to 2.3: Were these deviations likely to have affected the outcome?	PY
	2.5. If Y/PY/NI to 2.4: Were these deviations from intended intervention balanced between groups?	NI
	2.6 Was an appropriate analysis used to estimate the effect of assignment to intervention?	NA
	2.7 If N/PN/NI to 2.6: Was there potential for a substantial impact (on the result) of the failure to analyse participants in the group to which they were randomized?	N
	**Risk of bias judgement**	Low
Bias due to missing outcome data	3.1 Were data for this outcome available for all, or nearly all, participants randomized?	Y
	3.2 If N/PN/NI to 3.1: Is there evidence that result was not biased by missing outcome data?	PN
	3.3 If N/PN to 3.2: Could missingness in the outcome depend on its true value?	NA
	3.4 If Y/PY/NI to 3.3: Is it likely that missingness in the outcome depended on its true value?	N
	**Risk of bias judgement**	Low
Bias in measurement of the outcome	4.1 Was the method of measuring the outcome inappropriate?	N
	4.2 Could measurement or ascertainment of the outcome have differed between intervention groups?	NA
	4.3 Were outcome assessors aware of the intervention received by study participants?	N
	4.4 If Y/PY/NI to 4.3: Could assessment of the outcome have been influenced by knowledge of intervention received?	PY
	4.5 If Y/PY/NI to 4.4: Is it likely that assessment of the outcome was influenced by knowledge of intervention received?	Y
	**Risk of bias judgement**	Low
Bias in selection of the reported result	5.1 Were the data that produced this result analysed in accordance with a pre-specified analysis plan that was finalized before unblinded outcome data were	Y
	5.2 ... multiple eligible outcome measurements (e.g. scales, definitions, time points) within the outcome domain?	Y
	5.3 ... multiple eligible analyses of the data?	NA
	**Risk of bias judgement**	Some concerns
Overall bias	**Risk of bias judgement**	Some concerns

Study	Yijingting(2016)	
Domain	Signalling question	Responese
Bias arising from the randomization process	1.1 Was the allocation sequence random?	Y
	1.2 Was the allocation sequence concealed until participants were enrolled and assigned to interventions?	NA
	1.3 Did baseline differences between intervention groups suggest a problem with the randomization process?	NA
	**Risk of bias judgement**	Some concerns
Bias due to deviations from intended interventions	2.1.Were participants aware of their assigned intervention during the trial?	Y
	2.2.Were carers and people delivering the interventions aware of participants' assigned intervention during the trial?	Y
	2.3. If Y/PY/NI to 2.1 or 2.2: Were there deviations from the intended intervention that arose because of the experimental context?	PY
	2.4 If Y/PY to 2.3: Were these deviations likely to have affected the outcome?	Y
	2.5. If Y/PY/NI to 2.4: Were these deviations from intended intervention balanced between groups?	NA
	2.6 Was an appropriate analysis used to estimate the effect of assignment to intervention?	Y
	2.7 If N/PN/NI to 2.6: Was there potential for a substantial impact (on the result) of the failure to analyse participants in the group to which they were randomized?	NI
	**Risk of bias judgement**	Low
Bias due to missing outcome data	3.1 Were data for this outcome available for all, or nearly all, participants randomized?	Y
	3.2 If N/PN/NI to 3.1: Is there evidence that result was not biased by missing outcome data?	PN
	3.3 If N/PN to 3.2: Could missingness in the outcome depend on its true value?	NA
	3.4 If Y/PY/NI to 3.3: Is it likely that missingness in the outcome depended on its true value?	N
	**Risk of bias judgement**	Low
Bias in measurement of the outcome	4.1 Was the method of measuring the outcome inappropriate?	N
	4.2 Could measurement or ascertainment of the outcome have differed between intervention groups?	NA
	4.3 Were outcome assessors aware of the intervention received by study participants?	N
	4.4 If Y/PY/NI to 4.3: Could assessment of the outcome have been influenced by knowledge of intervention received?	PY
	4.5 If Y/PY/NI to 4.4: Is it likely that assessment of the outcome was influenced by knowledge of intervention received?	NA
	**Risk of bias judgement**	Low
Bias in selection of the reported result	5.1 Were the data that produced this result analysed in accordance with a pre-specified analysis plan that was finalized before unblinded outcome data were	Y
	5.2 ... multiple eligible outcome measurements (e.g. scales, definitions, time points) within the outcome domain?	Y
	5.3 ... multiple eligible analyses of the data?	NA
	**Risk of bias judgement**	Some concerns
Overall bias	**Risk of bias judgement**	Some concerns
QKLI VS Ribavirin (n=2)		
Study	Guoyewei(2014)	
Domain	Signalling question	Responese
Bias arising from the randomization process	1.1 Was the allocation sequence random?	Y
	1.2 Was the allocation sequence concealed until participants were enrolled and assigned to interventions?	NA
	1.3 Did baseline differences between intervention groups suggest a problem with the randomization process?	NA
	**Risk of bias judgement**	Some concerns
	2.1.Were participants aware of their assigned intervention during the trial?	Y
	2.2.Were carers and people delivering the interventions aware of participants' assigned intervention during the trial?	Y

Bias due to deviations from intended interventions	2.3. If Y/PY/NI to 2.1 or 2.2: Were there deviations from the intended intervention that arose because of the experimental context?	PY
	2.4 If Y/PY to 2.3: Were these deviations likely to have affected the outcome?	Y
	2.5. If Y/PY/NI to 2.4: Were these deviations from intended intervention balanced between groups?	NA
	2.6 Was an appropriate analysis used to estimate the effect of assignment to intervention?	Y
	2.7 If N/PN/NI to 2.6: Was there potential for a substantial impact (on the result) of the failure to analyse participants in the group to which they were randomized?	NI
	**Risk of bias judgement**	Low
Bias due to missing outcome data	3.1 Were data for this outcome available for all, or nearly all, participants randomized?	Y
	3.2 If N/PN/NI to 3.1: Is there evidence that result was not biased by missing outcome data?	PN
	3.3 If N/PN to 3.2: Could missingness in the outcome depend on its true value?	NA
	3.4 If Y/PY/NI to 3.3: Is it likely that missingness in the outcome depended on its true value?	N
	**Risk of bias judgement**	Low
Bias in measurement of the outcome	4.1 Was the method of measuring the outcome inappropriate?	N
	4.2 Could measurement or ascertainment of the outcome have differed between intervention groups?	NA
	4.3 Were outcome assessors aware of the intervention received by study participants?	N
	4.4 If Y/PY/NI to 4.3: Could assessment of the outcome have been influenced by knowledge of intervention received?	PY
	4.5 If Y/PY/NI to 4.4: Is it likely that assessment of the outcome was influenced by knowledge of intervention received?	NA
	**Risk of bias judgement**	Low
Bias in selection of the reported result	5.1 Were the data that produced this result analysed in accordance with a pre-specified analysis plan that was finalized before unblinded outcome data were	Y
	5.2 ... multiple eligible outcome measurements (e.g. scales, definitions, time points) within the outcome domain?	Y
	5.3 ... multiple eligible analyses of the data?	NA
	**Risk of bias judgement**	Some concerns
Overall bias	**Risk of bias judgement**	Some concerns
Study	Panhong(2015)	
Domain	Signalling question	Responese
Bias arising from the randomization process	1.1 Was the allocation sequence random?	Y
	1.2 Was the allocation sequence concealed until participants were enrolled and assigned to interventions?	NA
	1.3 Did baseline differences between intervention groups suggest a problem with the randomization process?	NA
	**Risk of bias judgement**	Some concerns
Bias due to deviations from intended interventions	2.1.Were participants aware of their assigned intervention during the trial?	NI
	2.2.Were carers and people delivering the interventions aware of participants' assigned intervention during the trial?	NA
	2.3. If Y/PY/NI to 2.1 or 2.2: Were there deviations from the intended intervention that arose because of the experimental context?	NI
	2.4 If Y/PY to 2.3: Were these deviations likely to have affected the outcome?	PY
	2.5. If Y/PY/NI to 2.4: Were these deviations from intended intervention balanced between groups?	NI
	2.6 Was an appropriate analysis used to estimate the effect of assignment to intervention?	NA
	2.7 If N/PN/NI to 2.6: Was there potential for a substantial impact (on the result) of the failure to analyse participants in the group to which they were randomized?	PN
	**Risk of bias judgement**	Some concerns
Bias due to missing outcome data	3.1 Were data for this outcome available for all, or nearly all, participants randomized?	NA
	3.2 If N/PN/NI to 3.1: Is there evidence that result was not biased by missing outcome data?	N
	3.3 If N/PN to 3.2: Could missingness in the outcome depend on its true value?	NA
	3.4 If Y/PY/NI to 3.3: Is it likely that missingness in the outcome depended on its true value?	NA

outcome data	**Risk of bias judgement**	Some concerns
Bias in measurement of the outcome	4.1 Was the method of measuring the outcome inappropriate?	N
	4.2 Could measurement or ascertainment of the outcome have differed between intervention groups?	NA
	4.3 Were outcome assessors aware of the intervention received by study participants?	N
	4.4 If Y/PY/NI to 4.3: Could assessment of the outcome have been influenced by knowledge of intervention received?	PY
	4.5 If Y/PY/NI to 4.4: Is it likely that assessment of the outcome was influenced by knowledge of intervention received?	NA
	**Risk of bias judgement**	Low
Bias in selection of the reported result	5.1 Were the data that produced this result analysed in accordance with a pre-specified analysis plan that was finalized before unblinded outcome data were	Y
	5.2 ... multiple eligible outcome measurements (e.g. scales, definitions, time points) within the outcome domain?	Y
	5.3 ... multiple eligible analyses of the data?	NA
	**Risk of bias judgement**	Some concerns
Overall bias	**Risk of bias judgement**	Some concerns
RDNI VS Ribavirin (n=22)		
Study	Liuqin(2015)	
Domain	Signalling question	Responese
Bias arising from the randomization process	1.1 Was the allocation sequence random?	Y
	1.2 Was the allocation sequence concealed until participants were enrolled and assigned to interventions?	NA
	1.3 Did baseline differences between intervention groups suggest a problem with the randomization process?	NA
	**Risk of bias judgement**	Some concerns
Bias due to deviations from intended interventions	2.1.Were participants aware of their assigned intervention during the trial?	NA
	2.2.Were carers and people delivering the interventions aware of participants' assigned intervention during the trial?	NA
	2.3. If Y/PY/NI to 2.1 or 2.2: Were there deviations from the intended intervention that arose because of the experimental context?	PY
	2.4 If Y/PY to 2.3: Were these deviations likely to have affected the outcome?	Y
	2.5. If Y/PY/NI to 2.4: Were these deviations from intended intervention balanced between groups?	NA
	2.6 Was an appropriate analysis used to estimate the effect of assignment to intervention?	Y
	2.7 If N/PN/NI to 2.6: Was there potential for a substantial impact (on the result) of the failure to analyse participants in the group to which they were randomized?	NI
	**Risk of bias judgement**	Some concerns
Bias due to missing outcome data	3.1 Were data for this outcome available for all, or nearly all, participants randomized?	Y
	3.2 If N/PN/NI to 3.1: Is there evidence that result was not biased by missing outcome data?	PN
	3.3 If N/PN to 3.2: Could missingness in the outcome depend on its true value?	NA
	3.4 If Y/PY/NI to 3.3: Is it likely that missingness in the outcome depended on its true value?	N
	**Risk of bias judgement**	Low
Bias in measurement of the outcome	4.1 Was the method of measuring the outcome inappropriate?	NI
	4.2 Could measurement or ascertainment of the outcome have differed between intervention groups?	PY
	4.3 Were outcome assessors aware of the intervention received by study participants?	N
	4.4 If Y/PY/NI to 4.3: Could assessment of the outcome have been influenced by knowledge of intervention received?	PY
	4.5 If Y/PY/NI to 4.4: Is it likely that assessment of the outcome was influenced by knowledge of intervention received?	NA
	**Risk of bias judgement**	Some concerns

Bias in selection of the reported result	5.1 Were the data that produced this result analysed in accordance with a pre-specified analysis plan that was finalized before unblinded outcome data were	Y
	5.2 ... multiple eligible outcome measurements (e.g. scales, definitions, time points) within the outcome domain?	Y
	5.3 ... multiple eligible analyses of the data?	NA
	**Risk of bias judgement**	Some concerns
Overall bias	**Risk of bias judgement**	Some concerns
Study	Zhangxian(2013)	
Domain	Signalling question	Responese
Bias arising from the randomization process	1.1 Was the allocation sequence random?	Y
	1.2 Was the allocation sequence concealed until participants were enrolled and assigned to interventions?	NA
	1.3 Did baseline differences between intervention groups suggest a problem with the randomization process?	NA
	**Risk of bias judgement**	Some concerns
Bias due to deviations from intended interventions	2.1.Were participants aware of their assigned intervention during the trial?	NA
	2.2.Were carers and people delivering the interventions aware of participants' assigned intervention during the trial?	N
	2.3. If Y/PY/NI to 2.1 or 2.2: Were there deviations from the intended intervention that arose because of the experimental context?	PY
	2.4 If Y/PY to 2.3: Were these deviations likely to have affected the outcome?	PY
	2.5. If Y/PY/NI to 2.4: Were these deviations from intended intervention balanced between groups?	NA
	2.6 Was an appropriate analysis used to estimate the effect of assignment to intervention?	Y
	2.7 If N/PN/NI to 2.6: Was there potential for a substantial impact (on the result) of the failure to analyse participants in the group to which they were randomized?	NI
	**Risk of bias judgement**	Some concerns
Bias due to missing outcome data	3.1 Were data for this outcome available for all, or nearly all, participants randomized?	Y
	3.2 If N/PN/NI to 3.1: Is there evidence that result was not biased by missing outcome data?	PN
	3.3 If N/PN to 3.2: Could missingness in the outcome depend on its true value?	NA
	3.4 If Y/PY/NI to 3.3: Is it likely that missingness in the outcome depended on its true value?	N
	**Risk of bias judgement**	Low
Bias in measurement of the outcome	4.1 Was the method of measuring the outcome inappropriate?	N
	4.2 Could measurement or ascertainment of the outcome have differed between intervention groups?	NA
	4.3 Were outcome assessors aware of the intervention received by study participants?	N
	4.4 If Y/PY/NI to 4.3: Could assessment of the outcome have been influenced by knowledge of intervention received?	PY
	4.5 If Y/PY/NI to 4.4: Is it likely that assessment of the outcome was influenced by knowledge of intervention received?	NA
	**Risk of bias judgement**	Low
Bias in selection of the reported result	5.1 Were the data that produced this result analysed in accordance with a pre-specified analysis plan that was finalized before unblinded outcome data were	Y
	5.2 ... multiple eligible outcome measurements (e.g. scales, definitions, time points) within the outcome domain?	Y
	5.3 ... multiple eligible analyses of the data?	NA
	**Risk of bias judgement**	Some concerns
Overall bias	**Risk of bias judgement**	Some concerns
Study	Dingpei(2013)	

Domain	Signalling question	Responese
Bias arising from the randomization process	1.1 Was the allocation sequence random?	Y
	1.2 Was the allocation sequence concealed until participants were enrolled and assigned to interventions?	Y
	1.3 Did baseline differences between intervention groups suggest a problem with the randomization process?	NA
	**Risk of bias judgement**	Low
Bias due to deviations from intended interventions	2.1.Were participants aware of their assigned intervention during the trial?	Y
	2.2.Were carers and people delivering the interventions aware of participants' assigned intervention during the trial?	Y
	2.3. If Y/PY/NI to 2.1 or 2.2: Were there deviations from the intended intervention that arose because of the experimental context?	PY
	2.4 If Y/PY to 2.3: Were these deviations likely to have affected the outcome?	Y
	2.5. If Y/PY/NI to 2.4: Were these deviations from intended intervention balanced between groups?	NA
	2.6 Was an appropriate analysis used to estimate the effect of assignment to intervention?	Y
	2.7 If N/PN/NI to 2.6: Was there potential for a substantial impact (on the result) of the failure to analyse participants in the group to which they were randomized?	NI
	**Risk of bias judgement**	Low
Bias due to missing outcome data	3.1 Were data for this outcome available for all, or nearly all, participants randomized?	NI
	3.2 If N/PN/NI to 3.1: Is there evidence that result was not biased by missing outcome data?	PN
	3.3 If N/PN to 3.2: Could missingness in the outcome depend on its true value?	NA
	3.4 If Y/PY/NI to 3.3: Is it likely that missingness in the outcome depended on its true value?	Y
	**Risk of bias judgement**	Low
Bias in measurement of the outcome	4.1 Was the method of measuring the outcome inappropriate?	N
	4.2 Could measurement or ascertainment of the outcome have differed between intervention groups?	NA
	4.3 Were outcome assessors aware of the intervention received by study participants?	N
	4.4 If Y/PY/NI to 4.3: Could assessment of the outcome have been influenced by knowledge of intervention received?	PY
	4.5 If Y/PY/NI to 4.4: Is it likely that assessment of the outcome was influenced by knowledge of intervention received?	NA
	**Risk of bias judgement**	Low
Bias in selection of the reported result	5.1 Were the data that produced this result analysed in accordance with a pre-specified analysis plan that was finalized before unblinded outcome data were	Y
	5.2 ... multiple eligible outcome measurements (e.g. scales, definitions, time points) within the outcome domain?	Y
	5.3 ... multiple eligible analyses of the data?	NA
	**Risk of bias judgement**	Some concerns
Overall bias	**Risk of bias judgement**	Some concerns

Study	Liuhui(2015)	
Domain	Signalling question	Responese
Bias arising from the randomization process	1.1 Was the allocation sequence random?	Y
	1.2 Was the allocation sequence concealed until participants were enrolled and assigned to interventions?	NA
	1.3 Did baseline differences between intervention groups suggest a problem with the randomization process?	NA
	**Risk of bias judgement**	Some concerns
Bias due to deviations from intended interventions	2.1.Were participants aware of their assigned intervention during the trial?	NA
	2.2.Were carers and people delivering the interventions aware of participants' assigned intervention during the trial?	N
	2.3. If Y/PY/NI to 2.1 or 2.2: Were there deviations from the intended intervention that arose because of the experimental context?	PY
	2.4 If Y/PY to 2.3: Were these deviations likely to have affected the outcome?	PY
	2.5. If Y/PY/NI to 2.4: Were these deviations from intended intervention balanced between groups?	NA
	2.6 Was an appropriate analysis used to estimate the effect of assignment to intervention?	Y

interventions	2.7 If N/PN/NI to 2.6: Was there potential for a substantial impact (on the result) of the failure to analyse participants in the group to which they were randomized?	NI
	**Risk of bias judgement**	Some concerns
Bias due to missing outcome data	3.1 Were data for this outcome available for all, or nearly all, participants randomized?	Y
	3.2 If N/PN/NI to 3.1: Is there evidence that result was not biased by missing outcome data?	PN
	3.3 If N/PN to 3.2: Could missingness in the outcome depend on its true value?	NA
	3.4 If Y/PY/NI to 3.3: Is it likely that missingness in the outcome depended on its true value?	N
	**Risk of bias judgement**	Low
Bias in measurement of the outcome	4.1 Was the method of measuring the outcome inappropriate?	N
	4.2 Could measurement or ascertainment of the outcome have differed between intervention groups?	NA
	4.3 Were outcome assessors aware of the intervention received by study participants?	N
	4.4 If Y/PY/NI to 4.3: Could assessment of the outcome have been influenced by knowledge of intervention received?	PY
	4.5 If Y/PY/NI to 4.4: Is it likely that assessment of the outcome was influenced by knowledge of intervention received?	NA
	**Risk of bias judgement**	Low
Bias in selection of the reported result	5.1 Were the data that produced this result analysed in accordance with a pre-specified analysis plan that was finalized before unblinded outcome data were	Y
	5.2 ... multiple eligible outcome measurements (e.g. scales, definitions, time points) within the outcome domain?	Y
	5.3 ... multiple eligible analyses of the data?	NA
	**Risk of bias judgement**	Some concerns
Overall bias	**Risk of bias judgement**	Some concerns
Study	Zhouwenwen(2014)	
Domain	Signalling question	Responese
Bias arising from the randomization process	1.1 Was the allocation sequence random?	Y
	1.2 Was the allocation sequence concealed until participants were enrolled and assigned to interventions?	NA
	1.3 Did baseline differences between intervention groups suggest a problem with the randomization process?	NA
	**Risk of bias judgement**	Some concerns
Bias due to deviations from intended interventions	2.1.Were participants aware of their assigned intervention during the trial?	NA
	2.2.Were carers and people delivering the interventions aware of participants' assigned intervention during the trial?	NA
	2.3. If Y/PY/NI to 2.1 or 2.2: Were there deviations from the intended intervention that arose because of the experimental context?	PY
	2.4 If Y/PY to 2.3: Were these deviations likely to have affected the outcome?	Y
	2.5. If Y/PY/NI to 2.4: Were these deviations from intended intervention balanced between groups?	NA
	2.6 Was an appropriate analysis used to estimate the effect of assignment to intervention?	Y
	2.7 If N/PN/NI to 2.6: Was there potential for a substantial impact (on the result) of the failure to analyse participants in the group to which they were randomized?	NI
	**Risk of bias judgement**	Some concerns
Bias due to missing outcome data	3.1 Were data for this outcome available for all, or nearly all, participants randomized?	Y
	3.2 If N/PN/NI to 3.1: Is there evidence that result was not biased by missing outcome data?	PN
	3.3 If N/PN to 3.2: Could missingness in the outcome depend on its true value?	NA
	3.4 If Y/PY/NI to 3.3: Is it likely that missingness in the outcome depended on its true value?	N
	**Risk of bias judgement**	Low
Bias in	4.1 Was the method of measuring the outcome inappropriate?	N
	4.2 Could measurement or ascertainment of the outcome have differed between intervention groups?	NA

measurement of the outcome	4.3 Were outcome assessors aware of the intervention received by study participants?	N
	4.4 If Y/PY/NI to 4.3: Could assessment of the outcome have been influenced by knowledge of intervention received?	PY
	4.5 If Y/PY/NI to 4.4: Is it likely that assessment of the outcome was influenced by knowledge of intervention received?	NA
	**Risk of bias judgement**	Low
Bias in selection of the reported result	5.1 Were the data that produced this result analysed in accordance with a pre-specified analysis plan that was finalized before unblinded outcome data were	Y
	5.2 ... multiple eligible outcome measurements (e.g. scales, definitions, time points) within the outcome domain?	Y
	5.3 ... multiple eligible analyses of the data?	NA
	**Risk of bias judgement**	Some concerns
Overall bias	**Risk of bias judgement**	Some concerns
Study	Cuibeiyong(2013)	
Domain	Signalling question	Responese
Bias arising from the randomization process	1.1 Was the allocation sequence random?	Y
	1.2 Was the allocation sequence concealed until participants were enrolled and assigned to interventions?	Y
	1.3 Did baseline differences between intervention groups suggest a problem with the randomization process?	Y
	**Risk of bias judgement**	Low
Bias due to deviations from intended interventions	2.1.Were participants aware of their assigned intervention during the trial?	NA
	2.2.Were carers and people delivering the interventions aware of participants' assigned intervention during the trial?	N
	2.3. If Y/PY/NI to 2.1 or 2.2: Were there deviations from the intended intervention that arose because of the experimental context?	PY
	2.4 If Y/PY to 2.3: Were these deviations likely to have affected the outcome?	Y
	2.5. If Y/PY/NI to 2.4: Were these deviations from intended intervention balanced between groups?	NA
	2.6 Was an appropriate analysis used to estimate the effect of assignment to intervention?	Y
	2.7 If N/PN/NI to 2.6: Was there potential for a substantial impact (on the result) of the failure to analyse participants in the group to which they were randomized?	NI
	**Risk of bias judgement**	Some concerns
Bias due to missing outcome data	3.1 Were data for this outcome available for all, or nearly all, participants randomized?	Y
	3.2 If N/PN/NI to 3.1: Is there evidence that result was not biased by missing outcome data?	PN
	3.3 If N/PN to 3.2: Could missingness in the outcome depend on its true value?	NA
	3.4 If Y/PY/NI to 3.3: Is it likely that missingness in the outcome depended on its true value?	N
	**Risk of bias judgement**	Low
Bias in measurement of the outcome	4.1 Was the method of measuring the outcome inappropriate?	N
	4.2 Could measurement or ascertainment of the outcome have differed between intervention groups?	NA
	4.3 Were outcome assessors aware of the intervention received by study participants?	N
	4.4 If Y/PY/NI to 4.3: Could assessment of the outcome have been influenced by knowledge of intervention received?	PY
	4.5 If Y/PY/NI to 4.4: Is it likely that assessment of the outcome was influenced by knowledge of intervention received?	NA
	**Risk of bias judgement**	Low
Bias in selection of the reported result	5.1 Were the data that produced this result analysed in accordance with a pre-specified analysis plan that was finalized before unblinded outcome data were	Y
	5.2 ... multiple eligible outcome measurements (e.g. scales, definitions, time points) within the outcome domain?	Y
	5.3 ... multiple eligible analyses of the data?	NA
	**Risk of bias judgement**	Some concerns
Overall bias	**Risk of bias judgement**	Some concerns

Study	Liuhaiqin(2013)	
Domain	Signalling question	Responese
Bias arising from the randomization process	1.1 Was the allocation sequence random?	Y
	1.2 Was the allocation sequence concealed until participants were enrolled and assigned to interventions?	NA
	1.3 Did baseline differences between intervention groups suggest a problem with the randomization process?	NA
	**Risk of bias judgement**	Some concerns
Bias due to deviations from intended interventions	2.1.Were participants aware of their assigned intervention during the trial?	N
	2.2.Were carers and people delivering the interventions aware of participants' assigned intervention during the trial?	N
	2.3. If Y/PY/NI to 2.1 or 2.2: Were there deviations from the intended intervention that arose because of the experimental context?	NI
	2.4 If Y/PY to 2.3: Were these deviations likely to have affected the outcome?	PY
	2.5. If Y/PY/NI to 2.4: Were these deviations from intended intervention balanced between groups?	I
	2.6 Was an appropriate analysis used to estimate the effect of assignment to intervention?	NI
	2.7 If N/PN/NI to 2.6: Was there potential for a substantial impact (on the result) of the failure to analyse participants in the group to which they were randomized?	N
	**Risk of bias judgement**	Some concerns
Bias due to missing outcome data	3.1 Were data for this outcome available for all, or nearly all, participants randomized?	Y
	3.2 If N/PN/NI to 3.1: Is there evidence that result was not biased by missing outcome data?	PN
	3.3 If N/PN to 3.2: Could missingness in the outcome depend on its true value?	NA
	3.4 If Y/PY/NI to 3.3: Is it likely that missingness in the outcome depended on its true value?	N
	**Risk of bias judgement**	Low
Bias in measurement of the outcome	4.1 Was the method of measuring the outcome inappropriate?	N
	4.2 Could measurement or ascertainment of the outcome have differed between intervention groups?	NA
	4.3 Were outcome assessors aware of the intervention received by study participants?	N
	4.4 If Y/PY/NI to 4.3: Could assessment of the outcome have been influenced by knowledge of intervention received?	PY
	4.5 If Y/PY/NI to 4.4: Is it likely that assessment of the outcome was influenced by knowledge of intervention received?	NA
	**Risk of bias judgement**	Low
Bias in selection of the reported result	5.1 Were the data that produced this result analysed in accordance with a pre-specified analysis plan that was finalized before unblinded outcome data were	Y
	5.2 ... multiple eligible outcome measurements (e.g. scales, definitions, time points) within the outcome domain?	Y
	5.3 ... multiple eligible analyses of the data?	NA
	**Risk of bias judgement**	Some concerns
Overall bias	**Risk of bias judgement**	Some concerns
Study	Huchunxia(2014)	
Domain	Signalling question	Responese
Bias arising from the randomization process	1.1 Was the allocation sequence random?	Y
	1.2 Was the allocation sequence concealed until participants were enrolled and assigned to interventions?	NA
	1.3 Did baseline differences between intervention groups suggest a problem with the randomization process?	NA
	**Risk of bias judgement**	Some concerns
	2.1.Were participants aware of their assigned intervention during the trial?	N

Bias due to deviations from intended interventions	2.2.Were carers and people delivering the interventions aware of participants' assigned intervention during the trial?	NA
	2.3. If Y/PY/NI to 2.1 or 2.2: Were there deviations from the intended intervention that arose because of the experimental context?	PY
	2.4 If Y/PY to 2.3: Were these deviations likely to have affected the outcome?	PY
	2.5. If Y/PY/NI to 2.4: Were these deviations from intended intervention balanced between groups?	NA
	2.6 Was an appropriate analysis used to estimate the effect of assignment to intervention?	Y
	2.7 If N/PN/NI to 2.6: Was there potential for a substantial impact (on the result) of the failure to analyse participants in the group to which they were randomized?	NI
	**Risk of bias judgement**	Some concerns
Bias due to missing outcome data	3.1 Were data for this outcome available for all, or nearly all, participants randomized?	NA
	3.2 If N/PN/NI to 3.1: Is there evidence that result was not biased by missing outcome data?	PN
	3.3 If N/PN to 3.2: Could missingness in the outcome depend on its true value?	NA
	3.4 If Y/PY/NI to 3.3: Is it likely that missingness in the outcome depended on its true value?	N
	**Risk of bias judgement**	Some concerns
Bias in measurement of the outcome	4.1 Was the method of measuring the outcome inappropriate?	N
	4.2 Could measurement or ascertainment of the outcome have differed between intervention groups?	NA
	4.3 Were outcome assessors aware of the intervention received by study participants?	N
	4.4 If Y/PY/NI to 4.3: Could assessment of the outcome have been influenced by knowledge of intervention received?	PY
	4.5 If Y/PY/NI to 4.4: Is it likely that assessment of the outcome was influenced by knowledge of intervention received?	NA
	**Risk of bias judgement**	Low
Bias in selection of the reported result	5.1 Were the data that produced this result analysed in accordance with a pre-specified analysis plan that was finalized before unblinded outcome data were	Y
	5.2 ... multiple eligible outcome measurements (e.g. scales, definitions, time points) within the outcome domain?	Y
	5.3 ... multiple eligible analyses of the data?	NA
	**Risk of bias judgement**	Some concerns
Overall bias	**Risk of bias judgement**	Some concerns
Study	Limingchun(2014)	
Domain	Signalling question	Responese
Bias arising from the randomization process	1.1 Was the allocation sequence random?	Y
	1.2 Was the allocation sequence concealed until participants were enrolled and assigned to interventions?	NA
	1.3 Did baseline differences between intervention groups suggest a problem with the randomization process?	NA
	**Risk of bias judgement**	Some concerns
Bias due to deviations from intended interventions	2.1.Were participants aware of their assigned intervention during the trial?	NA
	2.2.Were carers and people delivering the interventions aware of participants' assigned intervention during the trial?	NA
	2.3. If Y/PY/NI to 2.1 or 2.2: Were there deviations from the intended intervention that arose because of the experimental context?	PY
	2.4 If Y/PY to 2.3: Were these deviations likely to have affected the outcome?	PY
	2.5. If Y/PY/NI to 2.4: Were these deviations from intended intervention balanced between groups?	NA
	2.6 Was an appropriate analysis used to estimate the effect of assignment to intervention?	Y
	2.7 If N/PN/NI to 2.6: Was there potential for a substantial impact (on the result) of the failure to analyse participants in the group to which they were randomized?	NI
	**Risk of bias judgement**	Some concerns
	3.1 Were data for this outcome available for all, or nearly all, participants randomized?	Y

Bias due to missing outcome data	3.2 If N/PN/NI to 3.1: Is there evidence that result was not biased by missing outcome data?	PN
	3.3 If N/PN to 3.2: Could missingness in the outcome depend on its true value?	NA
	3.4 If Y/PY/NI to 3.3: Is it likely that missingness in the outcome depended on its true value?	N
	**Risk of bias judgement**	Low
Bias in measurement of the outcome	4.1 Was the method of measuring the outcome inappropriate?	N
	4.2 Could measurement or ascertainment of the outcome have differed between intervention groups?	NA
	4.3 Were outcome assessors aware of the intervention received by study participants?	N
	4.4 If Y/PY/NI to 4.3: Could assessment of the outcome have been influenced by knowledge of intervention received?	PY
	4.5 If Y/PY/NI to 4.4: Is it likely that assessment of the outcome was influenced by knowledge of intervention received?	NA
	**Risk of bias judgement**	Low
Bias in selection of the reported result	5.1 Were the data that produced this result analysed in accordance with a pre-specified analysis plan that was finalized before unblinded outcome data were	Y
	5.2 ... multiple eligible outcome measurements (e.g. scales, definitions, time points) within the outcome domain?	Y
	5.3 ... multiple eligible analyses of the data?	NA
	**Risk of bias judgement**	Some concerns
Overall bias	**Risk of bias judgement**	Some concerns
Study	Guyuxing(2013)	
Domain	Signalling question	Responese
Bias arising from the randomization process	1.1 Was the allocation sequence random?	Y
	1.2 Was the allocation sequence concealed until participants were enrolled and assigned to interventions?	Y
	1.3 Did baseline differences between intervention groups suggest a problem with the randomization process?	Y
	**Risk of bias judgement**	Low
Bias due to deviations from intended interventions	2.1.Were participants aware of their assigned intervention during the trial?	Y
	2.2.Were carers and people delivering the interventions aware of participants' assigned intervention during the trial?	Y
	2.3. If Y/PY/NI to 2.1 or 2.2: Were there deviations from the intended intervention that arose because of the experimental context?	PY
	2.4 If Y/PY to 2.3: Were these deviations likely to have affected the outcome?	Y
	2.5. If Y/PY/NI to 2.4: Were these deviations from intended intervention balanced between groups?	NA
	2.6 Was an appropriate analysis used to estimate the effect of assignment to intervention?	Y
	2.7 If N/PN/NI to 2.6: Was there potential for a substantial impact (on the result) of the failure to analyse participants in the group to which they were randomized?	NI
	**Risk of bias judgement**	Low
Bias due to missing outcome data	3.1 Were data for this outcome available for all, or nearly all, participants randomized?	Y
	3.2 If N/PN/NI to 3.1: Is there evidence that result was not biased by missing outcome data?	PN
	3.3 If N/PN to 3.2: Could missingness in the outcome depend on its true value?	NA
	3.4 If Y/PY/NI to 3.3: Is it likely that missingness in the outcome depended on its true value?	N
	**Risk of bias judgement**	Low
Bias in measurement of the outcome	4.1 Was the method of measuring the outcome inappropriate?	N
	4.2 Could measurement or ascertainment of the outcome have differed between intervention groups?	NA
	4.3 Were outcome assessors aware of the intervention received by study participants?	N
	4.4 If Y/PY/NI to 4.3: Could assessment of the outcome have been influenced by knowledge of intervention received?	PY
	4.5 If Y/PY/NI to 4.4: Is it likely that assessment of the outcome was influenced by knowledge of intervention received?	NA
	**Risk of bias judgement**	Low
Bias in selection of	5.1 Were the data that produced this result analysed in accordance with a pre-specified analysis plan that was finalized before unblinded outcome data were	Y
	5.2 ... multiple eligible outcome measurements (e.g. scales, definitions, time points) within the outcome domain?	Y

of the reported result	5.3 ... multiple eligible analyses of the data?	NA
	**Risk of bias judgement**	Some concerns
Overall bias	**Risk of bias judgement**	Some concerns
Study	Muchunjie(2013)	
Domain	Signalling question	Responese
Bias arising from the randomization process	1.1 Was the allocation sequence random?	Y
	1.2 Was the allocation sequence concealed until participants were enrolled and assigned to interventions?	NA
	1.3 Did baseline differences between intervention groups suggest a problem with the randomization process?	NA
	**Risk of bias judgement**	Some concerns
Bias due to deviations from intended interventions	2.1.Were participants aware of their assigned intervention during the trial?	N
	2.2.Were carers and people delivering the interventions aware of participants' assigned intervention during the trial?	Y
	2.3. If Y/PY/NI to 2.1 or 2.2: Were there deviations from the intended intervention that arose because of the experimental context?	PY
	2.4 If Y/PY to 2.3: Were these deviations likely to have affected the outcome?	Y
	2.5. If Y/PY/NI to 2.4: Were these deviations from intended intervention balanced between groups?	NA
	2.6 Was an appropriate analysis used to estimate the effect of assignment to intervention?	Y
	2.7 If N/PN/NI to 2.6: Was there potential for a substantial impact (on the result) of the failure to analyse participants in the group to which they were randomized?	NI
	**Risk of bias judgement**	Some concerns
Bias due to missing outcome data	3.1 Were data for this outcome available for all, or nearly all, participants randomized?	Y
	3.2 If N/PN/NI to 3.1: Is there evidence that result was not biased by missing outcome data?	PN
	3.3 If N/PN to 3.2: Could missingness in the outcome depend on its true value?	NA
	3.4 If Y/PY/NI to 3.3: Is it likely that missingness in the outcome depended on its true value?	N
	**Risk of bias judgement**	Low
Bias in measurement of the outcome	4.1 Was the method of measuring the outcome inappropriate?	N
	4.2 Could measurement or ascertainment of the outcome have differed between intervention groups?	NA
	4.3 Were outcome assessors aware of the intervention received by study participants?	N
	4.4 If Y/PY/NI to 4.3: Could assessment of the outcome have been influenced by knowledge of intervention received?	PY
	4.5 If Y/PY/NI to 4.4: Is it likely that assessment of the outcome was influenced by knowledge of intervention received?	NA
	**Risk of bias judgement**	Low
Bias in selection of the reported result	5.1 Were the data that produced this result analysed in accordance with a pre-specified analysis plan that was finalized before unblinded outcome data were	Y
	5.2 ... multiple eligible outcome measurements (e.g. scales, definitions, time points) within the outcome domain?	Y
	5.3 ... multiple eligible analyses of the data?	NA
	**Risk of bias judgement**	Some concerns
Overall bias	**Risk of bias judgement**	Some concerns
Study	Zhaoqun(2018)	
Domain	Signalling question	Responese
Bias arising from	1.1 Was the allocation sequence random?	Y

the randomization process	1.2 Was the allocation sequence concealed until participants were enrolled and assigned to interventions?	Y
	1.3 Did baseline differences between intervention groups suggest a problem with the randomization process?	Y
	**Risk of bias judgement**	Low
Bias due to deviations from intended interventions	2.1.Were participants aware of their assigned intervention during the trial?	Y
	2.2.Were carers and people delivering the interventions aware of participants' assigned intervention during the trial?	Y
	2.3. If Y/PY/NI to 2.1 or 2.2: Were there deviations from the intended intervention that arose because of the experimental context?	PY
	2.4 If Y/PY to 2.3: Were these deviations likely to have affected the outcome?	Y
	2.5. If Y/PY/NI to 2.4: Were these deviations from intended intervention balanced between groups?	NA
	2.6 Was an appropriate analysis used to estimate the effect of assignment to intervention?	Y
	2.7 If N/PN/NI to 2.6: Was there potential for a substantial impact (on the result) of the failure to analyse participants in the group to which they were randomized?	NI
	**Risk of bias judgement**	Low
Bias due to missing outcome data	3.1 Were data for this outcome available for all, or nearly all, participants randomized?	Y
	3.2 If N/PN/NI to 3.1: Is there evidence that result was not biased by missing outcome data?	PN
	3.3 If N/PN to 3.2: Could missingness in the outcome depend on its true value?	NA
	3.4 If Y/PY/NI to 3.3: Is it likely that missingness in the outcome depended on its true value?	N
	**Risk of bias judgement**	Low
Bias in measurement of the outcome	4.1 Was the method of measuring the outcome inappropriate?	N
	4.2 Could measurement or ascertainment of the outcome have differed between intervention groups?	NA
	4.3 Were outcome assessors aware of the intervention received by study participants?	N
	4.4 If Y/PY/NI to 4.3: Could assessment of the outcome have been influenced by knowledge of intervention received?	PY
	4.5 If Y/PY/NI to 4.4: Is it likely that assessment of the outcome was influenced by knowledge of intervention received?	NA
	**Risk of bias judgement**	Some concerns
Bias in selection of the reported result	5.1 Were the data that produced this result analysed in accordance with a pre-specified analysis plan that was finalized before unblinded outcome data were	Y
	5.2 ... multiple eligible outcome measurements (e.g. scales, definitions, time points) within the outcome domain?	Y
	5.3 ... multiple eligible analyses of the data?	NA
	**Risk of bias judgement**	Some concerns
Overall bias	**Risk of bias judgement**	Some concerns
Study	Xudongsheng(2015)	
Domain	Signalling question	Responese
Bias arising from the randomization process	1.1 Was the allocation sequence random?	Y
	1.2 Was the allocation sequence concealed until participants were enrolled and assigned to interventions?	NA
	1.3 Did baseline differences between intervention groups suggest a problem with the randomization process?	NA
	**Risk of bias judgement**	Some concerns
Bias due to deviations from intended interventions	2.1.Were participants aware of their assigned intervention during the trial?	Y
	2.2.Were carers and people delivering the interventions aware of participants' assigned intervention during the trial?	Y
	2.3. If Y/PY/NI to 2.1 or 2.2: Were there deviations from the intended intervention that arose because of the experimental context?	PY
	2.4 If Y/PY to 2.3: Were these deviations likely to have affected the outcome?	Y
	2.5. If Y/PY/NI to 2.4: Were these deviations from intended intervention balanced between groups?	NA
	2.6 Was an appropriate analysis used to estimate the effect of assignment to intervention?	Y
	2.7 If N/PN/NI to 2.6: Was there potential for a substantial impact (on the result) of the failure to analyse participants in the group to which they were randomized?	NI

	**Risk of bias judgement**	Some concerns
Bias due to missing outcome data	3.1 Were data for this outcome available for all, or nearly all, participants randomized?	Y
	3.2 If N/PN/NI to 3.1: Is there evidence that result was not biased by missing outcome data?	PN
	3.3 If N/PN to 3.2: Could missingness in the outcome depend on its true value?	NA
	3.4 If Y/PY/NI to 3.3: Is it likely that missingness in the outcome depended on its true value?	N
	**Risk of bias judgement**	Low
Bias in measurement of the outcome	4.1 Was the method of measuring the outcome inappropriate?	N
	4.2 Could measurement or ascertainment of the outcome have differed between intervention groups?	NA
	4.3 Were outcome assessors aware of the intervention received by study participants?	N
	4.4 If Y/PY/NI to 4.3: Could assessment of the outcome have been influenced by knowledge of intervention received?	PY
	4.5 If Y/PY/NI to 4.4: Is it likely that assessment of the outcome was influenced by knowledge of intervention received?	NA
	**Risk of bias judgement**	Low
Bias in selection of the reported result	5.1 Were the data that produced this result analysed in accordance with a pre-specified analysis plan that was finalized before unblinded outcome data were	Y
	5.2 ... multiple eligible outcome measurements (e.g. scales, definitions, time points) within the outcome domain?	Y
	5.3 ... multiple eligible analyses of the data?	NA
	**Risk of bias judgement**	Some concerns
Overall bias	**Risk of bias judgement**	Some concerns
Study	Zhuangtao(2015)	
Domain	Signalling question	Responese
Bias arising from the randomization process	1.1 Was the allocation sequence random?	Y
	1.2 Was the allocation sequence concealed until participants were enrolled and assigned to interventions?	Y
	1.3 Did baseline differences between intervention groups suggest a problem with the randomization process?	Y
	**Risk of bias judgement**	Low
Bias due to deviations from intended interventions	2.1.Were participants aware of their assigned intervention during the trial?	NA
	2.2.Were carers and people delivering the interventions aware of participants' assigned intervention during the trial?	NA
	2.3. If Y/PY/NI to 2.1 or 2.2: Were there deviations from the intended intervention that arose because of the experimental context?	PY
	2.4 If Y/PY to 2.3: Were these deviations likely to have affected the outcome?	Y
	2.5. If Y/PY/NI to 2.4: Were these deviations from intended intervention balanced between groups?	NA
	2.6 Was an appropriate analysis used to estimate the effect of assignment to intervention?	PY
	2.7 If N/PN/NI to 2.6: Was there potential for a substantial impact (on the result) of the failure to analyse participants in the group to which they were randomized?	NI
	**Risk of bias judgement**	Some concerns
Bias due to missing outcome data	3.1 Were data for this outcome available for all, or nearly all, participants randomized?	Y
	3.2 If N/PN/NI to 3.1: Is there evidence that result was not biased by missing outcome data?	PN
	3.3 If N/PN to 3.2: Could missingness in the outcome depend on its true value?	NA
	3.4 If Y/PY/NI to 3.3: Is it likely that missingness in the outcome depended on its true value?	N
	**Risk of bias judgement**	Low
Bias in measurement of the outcome	4.1 Was the method of measuring the outcome inappropriate?	N
	4.2 Could measurement or ascertainment of the outcome have differed between intervention groups?	NA
	4.3 Were outcome assessors aware of the intervention received by study participants?	N
	4.4 If Y/PY/NI to 4.3: Could assessment of the outcome have been influenced by knowledge of intervention received?	PY

	4.5 If Y/PY/NI to 4.4: Is it likely that assessment of the outcome was influenced by knowledge of intervention received?	NA
	**Risk of bias judgement**	Low
Bias in selection of the reported result	5.1 Were the data that produced this result analysed in accordance with a pre-specified analysis plan that was finalized before unblinded outcome data were	Y
	5.2 ... multiple eligible outcome measurements (e.g. scales, definitions, time points) within the outcome domain?	Y
	5.3 ... multiple eligible analyses of the data?	NA
	**Risk of bias judgement**	Some concerns
Overall bias	**Risk of bias judgement**	Some concerns
Study	Fangwanfen(2015)	
Domain	Signalling question	Responese
Bias arising from the randomization process	1.1 Was the allocation sequence random?	Y
	1.2 Was the allocation sequence concealed until participants were enrolled and assigned to interventions?	NA
	1.3 Did baseline differences between intervention groups suggest a problem with the randomization process?	NA
	**Risk of bias judgement**	Some concerns
Bias due to deviations from intended interventions	2.1.Were participants aware of their assigned intervention during the trial?	NA
	2.2.Were carers and people delivering the interventions aware of participants' assigned intervention during the trial?	NA
	2.3. If Y/PY/NI to 2.1 or 2.2: Were there deviations from the intended intervention that arose because of the experimental context?	PY
	2.4 If Y/PY to 2.3: Were these deviations likely to have affected the outcome?	N
	2.5. If Y/PY/NI to 2.4: Were these deviations from intended intervention balanced between groups?	NA
	2.6 Was an appropriate analysis used to estimate the effect of assignment to intervention?	Y
	2.7 If N/PN/NI to 2.6: Was there potential for a substantial impact (on the result) of the failure to analyse participants in the group to which they were randomized?	PY
	**Risk of bias judgement**	Some concerns
Bias due to missing outcome data	3.1 Were data for this outcome available for all, or nearly all, participants randomized?	Y
	3.2 If N/PN/NI to 3.1: Is there evidence that result was not biased by missing outcome data?	PN
	3.3 If N/PN to 3.2: Could missingness in the outcome depend on its true value?	NA
	3.4 If Y/PY/NI to 3.3: Is it likely that missingness in the outcome depended on its true value?	N
	**Risk of bias judgement**	Low
Bias in measurement of the outcome	4.1 Was the method of measuring the outcome inappropriate?	N
	4.2 Could measurement or ascertainment of the outcome have differed between intervention groups?	NA
	4.3 Were outcome assessors aware of the intervention received by study participants?	N
	4.4 If Y/PY/NI to 4.3: Could assessment of the outcome have been influenced by knowledge of intervention received?	PY
	4.5 If Y/PY/NI to 4.4: Is it likely that assessment of the outcome was influenced by knowledge of intervention received?	NA
	**Risk of bias judgement**	Low
Bias in selection of the reported result	5.1 Were the data that produced this result analysed in accordance with a pre-specified analysis plan that was finalized before unblinded outcome data were	Y
	5.2 ... multiple eligible outcome measurements (e.g. scales, definitions, time points) within the outcome domain?	Y
	5.3 ... multiple eligible analyses of the data?	NA
	**Risk of bias judgement**	Low
Overall bias	**Risk of bias judgement**	Some concerns

Study	Lihao(2017)	
Domain	Signalling question	Responese
Bias arising from the randomization process	1.1 Was the allocation sequence random?	Y
	1.2 Was the allocation sequence concealed until participants were enrolled and assigned to interventions?	NA
	1.3 Did baseline differences between intervention groups suggest a problem with the randomization process?	NA
	**Risk of bias judgement**	Some concerns
Bias due to deviations from intended interventions	2.1.Were participants aware of their assigned intervention during the trial?	N
	2.2.Were carers and people delivering the interventions aware of participants' assigned intervention during the trial?	N
	2.3. If Y/PY/NI to 2.1 or 2.2: Were there deviations from the intended intervention that arose because of the experimental context?	NA
	2.4 If Y/PY to 2.3: Were these deviations likely to have affected the outcome?	Y
	2.5. If Y/PY/NI to 2.4: Were these deviations from intended intervention balanced between groups?	NA
	2.6 Was an appropriate analysis used to estimate the effect of assignment to intervention?	PY
	2.7 If N/PN/NI to 2.6: Was there potential for a substantial impact (on the result) of the failure to analyse participants in the group to which they were randomized?	PN
	**Risk of bias judgement**	Some concerns
Bias due to missing outcome data	3.1 Were data for this outcome available for all, or nearly all, participants randomized?	Y
	3.2 If N/PN/NI to 3.1: Is there evidence that result was not biased by missing outcome data?	PN
	3.3 If N/PN to 3.2: Could missingness in the outcome depend on its true value?	NA
	3.4 If Y/PY/NI to 3.3: Is it likely that missingness in the outcome depended on its true value?	N
	**Risk of bias judgement**	Low
Bias in measurement of the outcome	4.1 Was the method of measuring the outcome inappropriate?	N
	4.2 Could measurement or ascertainment of the outcome have differed between intervention groups?	NA
	4.3 Were outcome assessors aware of the intervention received by study participants?	N
	4.4 If Y/PY/NI to 4.3: Could assessment of the outcome have been influenced by knowledge of intervention received?	PY
	4.5 If Y/PY/NI to 4.4: Is it likely that assessment of the outcome was influenced by knowledge of intervention received?	NA
	**Risk of bias judgement**	Low
Bias in selection of the reported result	5.1 Were the data that produced this result analysed in accordance with a pre-specified analysis plan that was finalized before unblinded outcome data were	Y
	5.2 ... multiple eligible outcome measurements (e.g. scales, definitions, time points) within the outcome domain?	Y
	5.3 ... multiple eligible analyses of the data?	NA
	**Risk of bias judgement**	Low
Overall bias	**Risk of bias judgement**	Some concerns
Study	Zhangzhigang(2019)	
Domain	Signalling question	Responese
Bias arising from the randomization process	1.1 Was the allocation sequence random?	Y
	1.2 Was the allocation sequence concealed until participants were enrolled and assigned to interventions?	NA
	1.3 Did baseline differences between intervention groups suggest a problem with the randomization process?	NA
	**Risk of bias judgement**	Some concerns
Bias due to deviations	2.1.Were participants aware of their assigned intervention during the trial?	NA
	2.2.Were carers and people delivering the interventions aware of participants' assigned intervention during the trial?	NA
	2.3. If Y/PY/NI to 2.1 or 2.2: Were there deviations from the intended intervention that arose because of the experimental context?	PY
	2.4 If Y/PY to 2.3: Were these deviations likely to have affected the outcome?	PY

deviations from intended interventions	2.5. If Y/PY/NI to 2.4: Were these deviations from intended intervention balanced between groups?	NA
	2.6 Was an appropriate analysis used to estimate the effect of assignment to intervention?	NA
	2.7 If N/PN/NI to 2.6: Was there potential for a substantial impact (on the result) of the failure to analyse participants in the group to which they were randomized?	NI
	**Risk of bias judgement**	Some concerns
Bias due to missing outcome data	3.1 Were data for this outcome available for all, or nearly all, participants randomized?	Y
	3.2 If N/PN/NI to 3.1: Is there evidence that result was not biased by missing outcome data?	PN
	3.3 If N/PN to 3.2: Could missingness in the outcome depend on its true value?	NA
	3.4 If Y/PY/NI to 3.3: Is it likely that missingness in the outcome depended on its true value?	N
	**Risk of bias judgement**	Low
Bias in measurement of the outcome	4.1 Was the method of measuring the outcome inappropriate?	N
	4.2 Could measurement or ascertainment of the outcome have differed between intervention groups?	NA
	4.3 Were outcome assessors aware of the intervention received by study participants?	N
	4.4 If Y/PY/NI to 4.3: Could assessment of the outcome have been influenced by knowledge of intervention received?	PY
	4.5 If Y/PY/NI to 4.4: Is it likely that assessment of the outcome was influenced by knowledge of intervention received?	NA
	**Risk of bias judgement**	Low
Bias in selection of the reported result	5.1 Were the data that produced this result analysed in accordance with a pre-specified analysis plan that was finalized before unblinded outcome data were	Y
	5.2 ... multiple eligible outcome measurements (e.g. scales, definitions, time points) within the outcome domain?	Y
	5.3 ... multiple eligible analyses of the data?	NA
	**Risk of bias judgement**	Low
Overall bias	**Risk of bias judgement**	Some concerns
Study	Zhangjie(2015)	
Domain	Signalling question	Responese
Bias arising from the randomization process	1.1 Was the allocation sequence random?	Y
	1.2 Was the allocation sequence concealed until participants were enrolled and assigned to interventions?	NA
	1.3 Did baseline differences between intervention groups suggest a problem with the randomization process?	NA
	**Risk of bias judgement**	Some concerns
Bias due to deviations from intended interventions	2.1.Were participants aware of their assigned intervention during the trial?	NA
	2.2.Were carers and people delivering the interventions aware of participants' assigned intervention during the trial?	N
	2.3. If Y/PY/NI to 2.1 or 2.2: Were there deviations from the intended intervention that arose because of the experimental context?	PY
	2.4 If Y/PY to 2.3: Were these deviations likely to have affected the outcome?	Y
	2.5. If Y/PY/NI to 2.4: Were these deviations from intended intervention balanced between groups?	NA
	2.6 Was an appropriate analysis used to estimate the effect of assignment to intervention?	Y
	2.7 If N/PN/NI to 2.6: Was there potential for a substantial impact (on the result) of the failure to analyse participants in the group to which they were randomized?	NI
	**Risk of bias judgement**	Some concerns
Bias due to missing outcome data	3.1 Were data for this outcome available for all, or nearly all, participants randomized?	Y
	3.2 If N/PN/NI to 3.1: Is there evidence that result was not biased by missing outcome data?	PN
	3.3 If N/PN to 3.2: Could missingness in the outcome depend on its true value?	NA
	3.4 If Y/PY/NI to 3.3: Is it likely that missingness in the outcome depended on its true value?	N
	**Risk of bias judgement**	Low
	4.1 Was the method of measuring the outcome inappropriate?	N

Bias in measurement of the outcome	4.2 Could measurement or ascertainment of the outcome have differed between intervention groups?	NA
	4.3 Were outcome assessors aware of the intervention received by study participants?	N
	4.4 If Y/PY/NI to 4.3: Could assessment of the outcome have been influenced by knowledge of intervention received?	PY
	4.5 If Y/PY/NI to 4.4: Is it likely that assessment of the outcome was influenced by knowledge of intervention received?	NA
	**Risk of bias judgement**	Low
Bias in selection of the reported result	5.1 Were the data that produced this result analysed in accordance with a pre-specified analysis plan that was finalized before unblinded outcome data were	Y
	5.2 ... multiple eligible outcome measurements (e.g. scales, definitions, time points) within the outcome domain?	Y
	5.3 ... multiple eligible analyses of the data?	NA
	**Risk of bias judgement**	Some concerns
Overall bias	**Risk of bias judgement**	Some concerns
Study	Liuyan(2015)	
Domain	Signalling question	Responese
Bias arising from the randomization process	1.1 Was the allocation sequence random?	Y
	1.2 Was the allocation sequence concealed until participants were enrolled and assigned to interventions?	NA
	1.3 Did baseline differences between intervention groups suggest a problem with the randomization process?	NA
	**Risk of bias judgement**	Some concerns
Bias due to deviations from intended interventions	2.1.Were participants aware of their assigned intervention during the trial?	Y
	2.2.Were carers and people delivering the interventions aware of participants' assigned intervention during the trial?	Y
	2.3. If Y/PY/NI to 2.1 or 2.2: Were there deviations from the intended intervention that arose because of the experimental context?	PY
	2.4 If Y/PY to 2.3: Were these deviations likely to have affected the outcome?	Y
	2.5. If Y/PY/NI to 2.4: Were these deviations from intended intervention balanced between groups?	NA
	2.6 Was an appropriate analysis used to estimate the effect of assignment to intervention?	Y
	2.7 If N/PN/NI to 2.6: Was there potential for a substantial impact (on the result) of the failure to analyse participants in the group to which they were randomized?	NI
	**Risk of bias judgement**	Low
Bias due to missing outcome data	3.1 Were data for this outcome available for all, or nearly all, participants randomized?	Y
	3.2 If N/PN/NI to 3.1: Is there evidence that result was not biased by missing outcome data?	PN
	3.3 If N/PN to 3.2: Could missingness in the outcome depend on its true value?	NA
	3.4 If Y/PY/NI to 3.3: Is it likely that missingness in the outcome depended on its true value?	N
	**Risk of bias judgement**	Low
Bias in measurement of the outcome	4.1 Was the method of measuring the outcome inappropriate?	N
	4.2 Could measurement or ascertainment of the outcome have differed between intervention groups?	NA
	4.3 Were outcome assessors aware of the intervention received by study participants?	N
	4.4 If Y/PY/NI to 4.3: Could assessment of the outcome have been influenced by knowledge of intervention received?	PY
	4.5 If Y/PY/NI to 4.4: Is it likely that assessment of the outcome was influenced by knowledge of intervention received?	NA
	**Risk of bias judgement**	Some concerns
Bias in selection of the reported result	5.1 Were the data that produced this result analysed in accordance with a pre-specified analysis plan that was finalized before unblinded outcome data were	Y
	5.2 ... multiple eligible outcome measurements (e.g. scales, definitions, time points) within the outcome domain?	Y
	5.3 ... multiple eligible analyses of the data?	NA
	**Risk of bias judgement**	Low

Overall bias	**Risk of bias judgement**	Some concerns
Study	Liubinxiu(2015)	
Domain	Signalling question	Responese
Bias arising from the randomization process	1.1 Was the allocation sequence random?	Y
	1.2 Was the allocation sequence concealed until participants were enrolled and assigned to interventions?	NA
	1.3 Did baseline differences between intervention groups suggest a problem with the randomization process?	NA
	**Risk of bias judgement**	Some concerns
Bias due to deviations from intended interventions	2.1.Were participants aware of their assigned intervention during the trial?	Y
	2.2.Were carers and people delivering the interventions aware of participants' assigned intervention during the trial?	Y
	2.3. If Y/PY/NI to 2.1 or 2.2: Were there deviations from the intended intervention that arose because of the experimental context?	PY
	2.4 If Y/PY to 2.3: Were these deviations likely to have affected the outcome?	Y
	2.5. If Y/PY/NI to 2.4: Were these deviations from intended intervention balanced between groups?	NA
	2.6 Was an appropriate analysis used to estimate the effect of assignment to intervention?	Y
	2.7 If N/PN/NI to 2.6: Was there potential for a substantial impact (on the result) of the failure to analyse participants in the group to which they were randomized?	NI
	**Risk of bias judgement**	Low
Bias due to missing outcome data	3.1 Were data for this outcome available for all, or nearly all, participants randomized?	Y
	3.2 If N/PN/NI to 3.1: Is there evidence that result was not biased by missing outcome data?	PN
	3.3 If N/PN to 3.2: Could missingness in the outcome depend on its true value?	NA
	3.4 If Y/PY/NI to 3.3: Is it likely that missingness in the outcome depended on its true value?	N
	**Risk of bias judgement**	Low
Bias in measurement of the outcome	4.1 Was the method of measuring the outcome inappropriate?	N
	4.2 Could measurement or ascertainment of the outcome have differed between intervention groups?	NA
	4.3 Were outcome assessors aware of the intervention received by study participants?	N
	4.4 If Y/PY/NI to 4.3: Could assessment of the outcome have been influenced by knowledge of intervention received?	PY
	4.5 If Y/PY/NI to 4.4: Is it likely that assessment of the outcome was influenced by knowledge of intervention received?	NA
	**Risk of bias judgement**	Low
Bias in selection of the reported result	5.1 Were the data that produced this result analysed in accordance with a pre-specified analysis plan that was finalized before unblinded outcome data were	Y
	5.2 ... multiple eligible outcome measurements (e.g. scales, definitions, time points) within the outcome domain?	Y
	5.3 ... multiple eligible analyses of the data?	NA
	**Risk of bias judgement**	Low
Overall bias	**Risk of bias judgement**	Some concerns
Study	Liuzhongyan(2015)	
Domain	Signalling question	Responese
Bias arising from the randomization process	1.1 Was the allocation sequence random?	Y
	1.2 Was the allocation sequence concealed until participants were enrolled and assigned to interventions?	NA
	1.3 Did baseline differences between intervention groups suggest a problem with the randomization process?	NA
	**Risk of bias judgement**	Some concerns
	2.1.Were participants aware of their assigned intervention during the trial?	NA

Bias due to deviations from intended interventions	2.2.Were carers and people delivering the interventions aware of participants' assigned intervention during the trial?	NA
	2.3. If Y/PY/NI to 2.1 or 2.2: Were there deviations from the intended intervention that arose because of the experimental context?	PY
	2.4 If Y/PY to 2.3: Were these deviations likely to have affected the outcome?	Y
	2.5. If Y/PY/NI to 2.4: Were these deviations from intended intervention balanced between groups?	NA
	2.6 Was an appropriate analysis used to estimate the effect of assignment to intervention?	N
	2.7 If N/PN/NI to 2.6: Was there potential for a substantial impact (on the result) of the failure to analyse participants in the group to which they were randomized?	PN
	**Risk of bias judgement**	Some concerns
Bias due to missing outcome data	3.1 Were data for this outcome available for all, or nearly all, participants randomized?	Y
	3.2 If N/PN/NI to 3.1: Is there evidence that result was not biased by missing outcome data?	PN
	3.3 If N/PN to 3.2: Could missingness in the outcome depend on its true value?	NA
	3.4 If Y/PY/NI to 3.3: Is it likely that missingness in the outcome depended on its true value?	N
	**Risk of bias judgement**	Low
Bias in measurement of the outcome	4.1 Was the method of measuring the outcome inappropriate?	N
	4.2 Could measurement or ascertainment of the outcome have differed between intervention groups?	NA
	4.3 Were outcome assessors aware of the intervention received by study participants?	N
	4.4 If Y/PY/NI to 4.3: Could assessment of the outcome have been influenced by knowledge of intervention received?	PY
	4.5 If Y/PY/NI to 4.4: Is it likely that assessment of the outcome was influenced by knowledge of intervention received?	NA
	**Risk of bias judgement**	Low
Bias in selection of the reported result	5.1 Were the data that produced this result analysed in accordance with a pre-specified analysis plan that was finalized before unblinded outcome data were	Y
	5.2 ... multiple eligible outcome measurements (e.g. scales, definitions, time points) within the outcome domain?	Y
	5.3 ... multiple eligible analyses of the data?	NA
	**Risk of bias judgement**	Low
Overall bias	**Risk of bias judgement**	Some concerns
	YHNI VS Ribavirin (n=22)	
Study	Weiyueru(2014)	
Domain	Signalling question	Responese
Bias arising from the randomization process	1.1 Was the allocation sequence random?	Y
	1.2 Was the allocation sequence concealed until participants were enrolled and assigned to interventions?	NA
	1.3 Did baseline differences between intervention groups suggest a problem with the randomization process?	NA
	**Risk of bias judgement**	Some concerns
Bias due to deviations from intended interventions	2.1.Were participants aware of their assigned intervention during the trial?	NA
	2.2.Were carers and people delivering the interventions aware of participants' assigned intervention during the trial?	NA
	2.3. If Y/PY/NI to 2.1 or 2.2: Were there deviations from the intended intervention that arose because of the experimental context?	PY
	2.4 If Y/PY to 2.3: Were these deviations likely to have affected the outcome?	PY
	2.5. If Y/PY/NI to 2.4: Were these deviations from intended intervention balanced between groups?	N
	2.6 Was an appropriate analysis used to estimate the effect of assignment to intervention?	Y
	2.7 If N/PN/NI to 2.6: Was there potential for a substantial impact (on the result) of the failure to analyse participants in the group to which they were randomized?	NI
	**Risk of bias judgement**	Some concerns
Bias due to	3.1 Were data for this outcome available for all, or nearly all, participants randomized?	Y
	3.2 If N/PN/NI to 3.1: Is there evidence that result was not biased by missing outcome data?	PN

missing outcome data	3.3 If N/PN to 3.2: Could missingness in the outcome depend on its true value?	NA
	3.4 If Y/PY/NI to 3.3: Is it likely that missingness in the outcome depended on its true value?	N
	**Risk of bias judgement**	Low
Bias in measurement of the outcome	4.1 Was the method of measuring the outcome inappropriate?	N
	4.2 Could measurement or ascertainment of the outcome have differed between intervention groups?	NA
	4.3 Were outcome assessors aware of the intervention received by study participants?	N
	4.4 If Y/PY/NI to 4.3: Could assessment of the outcome have been influenced by knowledge of intervention received?	PY
	4.5 If Y/PY/NI to 4.4: Is it likely that assessment of the outcome was influenced by knowledge of intervention received?	NA
	**Risk of bias judgement**	Low
Bias in selection of the reported result	5.1 Were the data that produced this result analysed in accordance with a pre-specified analysis plan that was finalized before unblinded outcome data were	NA
	5.2 ... multiple eligible outcome measurements (e.g. scales, definitions, time points) within the outcome domain?	N
	5.3 ... multiple eligible analyses of the data?	NA
	**Risk of bias judgement**	Low
Overall bias	**Risk of bias judgement**	Some concerns
Study	Wangguiying(2014)	
Domain	Signalling question	Responese
Bias arising from the randomization process	1.1 Was the allocation sequence random?	Y
	1.2 Was the allocation sequence concealed until participants were enrolled and assigned to interventions?	NA
	1.3 Did baseline differences between intervention groups suggest a problem with the randomization process?	NA
	**Risk of bias judgement**	Some concerns
Bias due to deviations from intended interventions	2.1.Were participants aware of their assigned intervention during the trial?	N
	2.2.Were carers and people delivering the interventions aware of participants' assigned intervention during the trial?	Y
	2.3. If Y/PY/NI to 2.1 or 2.2: Were there deviations from the intended intervention that arose because of the experimental context?	PY
	2.4 If Y/PY to 2.3: Were these deviations likely to have affected the outcome?	Y
	2.5. If Y/PY/NI to 2.4: Were these deviations from intended intervention balanced between groups?	NA
	2.6 Was an appropriate analysis used to estimate the effect of assignment to intervention?	Y
	2.7 If N/PN/NI to 2.6: Was there potential for a substantial impact (on the result) of the failure to analyse participants in the group to which they were randomized?	NI
	**Risk of bias judgement**	Some concerns
Bias due to missing outcome data	3.1 Were data for this outcome available for all, or nearly all, participants randomized?	Y
	3.2 If N/PN/NI to 3.1: Is there evidence that result was not biased by missing outcome data?	PN
	3.3 If N/PN to 3.2: Could missingness in the outcome depend on its true value?	NA
	3.4 If Y/PY/NI to 3.3: Is it likely that missingness in the outcome depended on its true value?	N
	**Risk of bias judgement**	Low
Bias in measurement of the outcome	4.1 Was the method of measuring the outcome inappropriate?	N
	4.2 Could measurement or ascertainment of the outcome have differed between intervention groups?	NA
	4.3 Were outcome assessors aware of the intervention received by study participants?	N
	4.4 If Y/PY/NI to 4.3: Could assessment of the outcome have been influenced by knowledge of intervention received?	PY
	4.5 If Y/PY/NI to 4.4: Is it likely that assessment of the outcome was influenced by knowledge of intervention received?	NA
	**Risk of bias judgement**	Low
Bias in selection of	5.1 Were the data that produced this result analysed in accordance with a pre-specified analysis plan that was finalized before unblinded outcome data were	Y
	5.2 ... multiple eligible outcome measurements (e.g. scales, definitions, time points) within the outcome domain?	Y

the reported result	5.3 ... multiple eligible analyses of the data?	NA
	**Risk of bias judgement**	Low
Overall bias	**Risk of bias judgement**	Some concerns
Study	Wuguobin(2013)	
Domain	Signalling question	Responese
Bias arising from the randomization process	1.1 Was the allocation sequence random?	Y
	1.2 Was the allocation sequence concealed until participants were enrolled and assigned to interventions?	NA
	1.3 Did baseline differences between intervention groups suggest a problem with the randomization process?	NA
	**Risk of bias judgement**	Some concerns
Bias due to deviations from intended interventions	2.1.Were participants aware of their assigned intervention during the trial?	NA
	2.2.Were carers and people delivering the interventions aware of participants' assigned intervention during the trial?	Y
	2.3. If Y/PY/NI to 2.1 or 2.2: Were there deviations from the intended intervention that arose because of the experimental context?	PY
	2.4 If Y/PY to 2.3: Were these deviations likely to have affected the outcome?	Y
	2.5. If Y/PY/NI to 2.4: Were these deviations from intended intervention balanced between groups?	NA
	2.6 Was an appropriate analysis used to estimate the effect of assignment to intervention?	Y
	2.7 If N/PN/NI to 2.6: Was there potential for a substantial impact (on the result) of the failure to analyse participants in the group to which they were randomized?	NI
	**Risk of bias judgement**	Some concerns
Bias due to missing outcome data	3.1 Were data for this outcome available for all, or nearly all, participants randomized?	Y
	3.2 If N/PN/NI to 3.1: Is there evidence that result was not biased by missing outcome data?	PN
	3.3 If N/PN to 3.2: Could missingness in the outcome depend on its true value?	NA
	3.4 If Y/PY/NI to 3.3: Is it likely that missingness in the outcome depended on its true value?	N
	**Risk of bias judgement**	Low
Bias in measurement of the outcome	4.1 Was the method of measuring the outcome inappropriate?	N
	4.2 Could measurement or ascertainment of the outcome have differed between intervention groups?	NA
	4.3 Were outcome assessors aware of the intervention received by study participants?	N
	4.4 If Y/PY/NI to 4.3: Could assessment of the outcome have been influenced by knowledge of intervention received?	PY
	4.5 If Y/PY/NI to 4.4: Is it likely that assessment of the outcome was influenced by knowledge of intervention received?	NA
	**Risk of bias judgement**	Low
Bias in selection of the reported result	5.1 Were the data that produced this result analysed in accordance with a pre-specified analysis plan that was finalized before unblinded outcome data were	Y
	5.2 ... multiple eligible outcome measurements (e.g. scales, definitions, time points) within the outcome domain?	Y
	5.3 ... multiple eligible analyses of the data?	NA
	**Risk of bias judgement**	Low
Overall bias	**Risk of bias judgement**	Some concerns
Study	Saiqin(2015)	
Domain	Signalling question	Responese
Bias arising from the randomization	1.1 Was the allocation sequence random?	Y
	1.2 Was the allocation sequence concealed until participants were enrolled and assigned to interventions?	NA
	1.3 Did baseline differences between intervention groups suggest a problem with the randomization process?	NA

randomization process	**Risk of bias judgement**	Some concerns
Bias due to deviations from intended interventions	2.1.Were participants aware of their assigned intervention during the trial?	NA
	2.2.Were carers and people delivering the interventions aware of participants' assigned intervention during the trial?	NA
	2.3. If Y/PY/NI to 2.1 or 2.2: Were there deviations from the intended intervention that arose because of the experimental context?	PY
	2.4 If Y/PY to 2.3: Were these deviations likely to have affected the outcome?	PY
	2.5. If Y/PY/NI to 2.4: Were these deviations from intended intervention balanced between groups?	NA
	2.6 Was an appropriate analysis used to estimate the effect of assignment to intervention?	Y
	2.7 If N/PN/NI to 2.6: Was there potential for a substantial impact (on the result) of the failure to analyse participants in the group to which they were randomized?	NI
	**Risk of bias judgement**	Some concerns
Bias due to missing outcome data	3.1 Were data for this outcome available for all, or nearly all, participants randomized?	Y
	3.2 If N/PN/NI to 3.1: Is there evidence that result was not biased by missing outcome data?	PN
	3.3 If N/PN to 3.2: Could missingness in the outcome depend on its true value?	NA
	3.4 If Y/PY/NI to 3.3: Is it likely that missingness in the outcome depended on its true value?	N
	**Risk of bias judgement**	Low
Bias in measurement of the outcome	4.1 Was the method of measuring the outcome inappropriate?	N
	4.2 Could measurement or ascertainment of the outcome have differed between intervention groups?	NA
	4.3 Were outcome assessors aware of the intervention received by study participants?	N
	4.4 If Y/PY/NI to 4.3: Could assessment of the outcome have been influenced by knowledge of intervention received?	PY
	4.5 If Y/PY/NI to 4.4: Is it likely that assessment of the outcome was influenced by knowledge of intervention received?	NA
	**Risk of bias judgement**	Low
Bias in selection of the reported result	5.1 Were the data that produced this result analysed in accordance with a pre-specified analysis plan that was finalized before unblinded outcome data were	Y
	5.2 ... multiple eligible outcome measurements (e.g. scales, definitions, time points) within the outcome domain?	Y
	5.3 ... multiple eligible analyses of the data?	NA
	**Risk of bias judgement**	Low
Overall bias	**Risk of bias judgement**	Some concerns
Study	Mushuyun(2013)	
Domain	Signalling question	Responese
Bias arising from the randomization process	1.1 Was the allocation sequence random?	Y
	1.2 Was the allocation sequence concealed until participants were enrolled and assigned to interventions?	NA
	1.3 Did baseline differences between intervention groups suggest a problem with the randomization process?	NA
	**Risk of bias judgement**	Some concerns
Bias due to deviations from intended interventions	2.1.Were participants aware of their assigned intervention during the trial?	NA
	2.2.Were carers and people delivering the interventions aware of participants' assigned intervention during the trial?	NA
	2.3. If Y/PY/NI to 2.1 or 2.2: Were there deviations from the intended intervention that arose because of the experimental context?	PY
	2.4 If Y/PY to 2.3: Were these deviations likely to have affected the outcome?	PY
	2.5. If Y/PY/NI to 2.4: Were these deviations from intended intervention balanced between groups?	NA
	2.6 Was an appropriate analysis used to estimate the effect of assignment to intervention?	Y
	2.7 If N/PN/NI to 2.6: Was there potential for a substantial impact (on the result) of the failure to analyse participants in the group to which they were randomized?	NI
	**Risk of bias judgement**	Some concerns

Bias due to missing outcome data	3.1 Were data for this outcome available for all, or nearly all, participants randomized?	Y
	3.2 If N/PN/NI to 3.1: Is there evidence that result was not biased by missing outcome data?	PN
	3.3 If N/PN to 3.2: Could missingness in the outcome depend on its true value?	NA
	3.4 If Y/PY/NI to 3.3: Is it likely that missingness in the outcome depended on its true value?	N
	**Risk of bias judgement**	Low
Bias in measurement of the outcome	4.1 Was the method of measuring the outcome inappropriate?	N
	4.2 Could measurement or ascertainment of the outcome have differed between intervention groups?	NA
	4.3 Were outcome assessors aware of the intervention received by study participants?	N
	4.4 If Y/PY/NI to 4.3: Could assessment of the outcome have been influenced by knowledge of intervention received?	PY
	4.5 If Y/PY/NI to 4.4: Is it likely that assessment of the outcome was influenced by knowledge of intervention received?	NA
	**Risk of bias judgement**	Low
Bias in selection of the reported result	5.1 Were the data that produced this result analysed in accordance with a pre-specified analysis plan that was finalized before unblinded outcome data were	Y
	5.2 ... multiple eligible outcome measurements (e.g. scales, definitions, time points) within the outcome domain?	Y
	5.3 ... multiple eligible analyses of the data?	NA
	**Risk of bias judgement**	Low
Overall bias	**Risk of bias judgement**	Some concerns
Study	Wangxinlian(2019)	
Domain	Signalling question	Responese
Bias arising from the randomization process	1.1 Was the allocation sequence random?	Y
	1.2 Was the allocation sequence concealed until participants were enrolled and assigned to interventions?	Y
	1.3 Did baseline differences between intervention groups suggest a problem with the randomization process?	Y
	**Risk of bias judgement**	Low
Bias due to deviations from intended interventions	2.1.Were participants aware of their assigned intervention during the trial?	Y
	2.2.Were carers and people delivering the interventions aware of participants' assigned intervention during the trial?	Y
	2.3. If Y/PY/NI to 2.1 or 2.2: Were there deviations from the intended intervention that arose because of the experimental context?	PY
	2.4 If Y/PY to 2.3: Were these deviations likely to have affected the outcome?	Y
	2.5. If Y/PY/NI to 2.4: Were these deviations from intended intervention balanced between groups?	NA
	2.6 Was an appropriate analysis used to estimate the effect of assignment to intervention?	Y
	2.7 If N/PN/NI to 2.6: Was there potential for a substantial impact (on the result) of the failure to analyse participants in the group to which they were randomized?	NI
	**Risk of bias judgement**	Some concerns
Bias due to missing outcome data	3.1 Were data for this outcome available for all, or nearly all, participants randomized?	Y
	3.2 If N/PN/NI to 3.1: Is there evidence that result was not biased by missing outcome data?	PN
	3.3 If N/PN to 3.2: Could missingness in the outcome depend on its true value?	NA
	3.4 If Y/PY/NI to 3.3: Is it likely that missingness in the outcome depended on its true value?	N
	**Risk of bias judgement**	Low
Bias in measurement of the outcome	4.1 Was the method of measuring the outcome inappropriate?	N
	4.2 Could measurement or ascertainment of the outcome have differed between intervention groups?	NA
	4.3 Were outcome assessors aware of the intervention received by study participants?	N
	4.4 If Y/PY/NI to 4.3: Could assessment of the outcome have been influenced by knowledge of intervention received?	PY
	4.5 If Y/PY/NI to 4.4: Is it likely that assessment of the outcome was influenced by knowledge of intervention received?	NA
	**Risk of bias judgement**	Low
Bias in selection	5.1 Were the data that produced this result analysed in accordance with a pre-specified analysis plan that was finalized before unblinded outcome data were	Y

Bias in selection of the reported result	5.2 ... multiple eligible outcome measurements (e.g. scales, definitions, time points) within the outcome domain?	Y
	5.3 ... multiple eligible analyses of the data?	NA
	**Risk of bias judgement**	Some concerns
Overall bias	**Risk of bias judgement**	Some concerns
Study	Miuchunjie(2013)	
Domain	Signalling question	Responese
Bias arising from the randomization process	1.1 Was the allocation sequence random?	Y
	1.2 Was the allocation sequence concealed until participants were enrolled and assigned to interventions?	NA
	1.3 Did baseline differences between intervention groups suggest a problem with the randomization process?	NA
	**Risk of bias judgement**	Some concerns
Bias due to deviations from intended interventions	2.1.Were participants aware of their assigned intervention during the trial?	Y
	2.2.Were carers and people delivering the interventions aware of participants' assigned intervention during the trial?	Y
	2.3. If Y/PY/NI to 2.1 or 2.2: Were there deviations from the intended intervention that arose because of the experimental context?	PY
	2.4 If Y/PY to 2.3: Were these deviations likely to have affected the outcome?	Y
	2.5. If Y/PY/NI to 2.4: Were these deviations from intended intervention balanced between groups?	NA
	2.6 Was an appropriate analysis used to estimate the effect of assignment to intervention?	Y
	2.7 If N/PN/NI to 2.6: Was there potential for a substantial impact (on the result) of the failure to analyse participants in the group to which they were randomized?	NI
	**Risk of bias judgement**	Low
Bias due to missing outcome data	3.1 Were data for this outcome available for all, or nearly all, participants randomized?	Y
	3.2 If N/PN/NI to 3.1: Is there evidence that result was not biased by missing outcome data?	PN
	3.3 If N/PN to 3.2: Could missingness in the outcome depend on its true value?	NA
	3.4 If Y/PY/NI to 3.3: Is it likely that missingness in the outcome depended on its true value?	N
	**Risk of bias judgement**	Low
Bias in measurement of the outcome	4.1 Was the method of measuring the outcome inappropriate?	N
	4.2 Could measurement or ascertainment of the outcome have differed between intervention groups?	NA
	4.3 Were outcome assessors aware of the intervention received by study participants?	N
	4.4 If Y/PY/NI to 4.3: Could assessment of the outcome have been influenced by knowledge of intervention received?	PY
	4.5 If Y/PY/NI to 4.4: Is it likely that assessment of the outcome was influenced by knowledge of intervention received?	NA
	**Risk of bias judgement**	Low
Bias in selection of the reported result	5.1 Were the data that produced this result analysed in accordance with a pre-specified analysis plan that was finalized before unblinded outcome data were	Y
	5.2 ... multiple eligible outcome measurements (e.g. scales, definitions, time points) within the outcome domain?	Y
	5.3 ... multiple eligible analyses of the data?	NA
	**Risk of bias judgement**	Some concerns
Overall bias	**Risk of bias judgement**	Some concerns
Study	Tangliqun(2013)	
Domain	Signalling question	Responese
Bias arising from	1.1 Was the allocation sequence random?	Y

Bias arising from the randomization process	1.2 Was the allocation sequence concealed until participants were enrolled and assigned to interventions?	NA
	1.3 Did baseline differences between intervention groups suggest a problem with the randomization process?	NA
	**Risk of bias judgement**	Some concerns
Bias due to deviations from intended interventions	2.1.Were participants aware of their assigned intervention during the trial?	Y
	2.2.Were carers and people delivering the interventions aware of participants' assigned intervention during the trial?	Y
	2.3. If Y/PY/NI to 2.1 or 2.2: Were there deviations from the intended intervention that arose because of the experimental context?	PY
	2.4 If Y/PY to 2.3: Were these deviations likely to have affected the outcome?	Y
	2.5. If Y/PY/NI to 2.4: Were these deviations from intended intervention balanced between groups?	NA
	2.6 Was an appropriate analysis used to estimate the effect of assignment to intervention?	Y
	2.7 If N/PN/NI to 2.6: Was there potential for a substantial impact (on the result) of the failure to analyse participants in the group to which they were randomized?	NI
	**Risk of bias judgement**	Low
Bias due to missing outcome data	3.1 Were data for this outcome available for all, or nearly all, participants randomized?	Y
	3.2 If N/PN/NI to 3.1: Is there evidence that result was not biased by missing outcome data?	PN
	3.3 If N/PN to 3.2: Could missingness in the outcome depend on its true value?	NA
	3.4 If Y/PY/NI to 3.3: Is it likely that missingness in the outcome depended on its true value?	N
	**Risk of bias judgement**	Low
Bias in measurement of the outcome	4.1 Was the method of measuring the outcome inappropriate?	N
	4.2 Could measurement or ascertainment of the outcome have differed between intervention groups?	NA
	4.3 Were outcome assessors aware of the intervention received by study participants?	N
	4.4 If Y/PY/NI to 4.3: Could assessment of the outcome have been influenced by knowledge of intervention received?	PY
	4.5 If Y/PY/NI to 4.4: Is it likely that assessment of the outcome was influenced by knowledge of intervention received?	NA
	**Risk of bias judgement**	Low
Bias in selection of the reported result	5.1 Were the data that produced this result analysed in accordance with a pre-specified analysis plan that was finalized before unblinded outcome data were	Y
	5.2 ... multiple eligible outcome measurements (e.g. scales, definitions, time points) within the outcome domain?	Y
	5.3 ... multiple eligible analyses of the data?	NA
	**Risk of bias judgement**	Low
Overall bias	**Risk of bias judgement**	Some concerns
Study	Kuangchaobo(2014)	
Domain	Signalling question	Responese
Bias arising from the randomization process	1.1 Was the allocation sequence random?	Y
	1.2 Was the allocation sequence concealed until participants were enrolled and assigned to interventions?	NA
	1.3 Did baseline differences between intervention groups suggest a problem with the randomization process?	NA
	**Risk of bias judgement**	Some concerns
Bias due to deviations from intended interventions	2.1.Were participants aware of their assigned intervention during the trial?	NA
	2.2.Were carers and people delivering the interventions aware of participants' assigned intervention during the trial?	NA
	2.3. If Y/PY/NI to 2.1 or 2.2: Were there deviations from the intended intervention that arose because of the experimental context?	PY
	2.4 If Y/PY to 2.3: Were these deviations likely to have affected the outcome?	Y
	2.5. If Y/PY/NI to 2.4: Were these deviations from intended intervention balanced between groups?	NA
	2.6 Was an appropriate analysis used to estimate the effect of assignment to intervention?	Y
	2.7 If N/PN/NI to 2.6: Was there potential for a substantial impact (on the result) of the failure to analyse participants in the group to which they were randomized?	NI

	**Risk of bias judgement**	Some concerns
Bias due to missing outcome data	3.1 Were data for this outcome available for all, or nearly all, participants randomized?	Y
	3.2 If N/PN/NI to 3.1: Is there evidence that result was not biased by missing outcome data?	PN
	3.3 If N/PN to 3.2: Could missingness in the outcome depend on its true value?	NA
	3.4 If Y/PY/NI to 3.3: Is it likely that missingness in the outcome depended on its true value?	N
	**Risk of bias judgement**	Low
Bias in measurement of the outcome	4.1 Was the method of measuring the outcome inappropriate?	N
	4.2 Could measurement or ascertainment of the outcome have differed between intervention groups?	NA
	4.3 Were outcome assessors aware of the intervention received by study participants?	N
	4.4 If Y/PY/NI to 4.3: Could assessment of the outcome have been influenced by knowledge of intervention received?	PY
	4.5 If Y/PY/NI to 4.4: Is it likely that assessment of the outcome was influenced by knowledge of intervention received?	NA
	**Risk of bias judgement**	Low
Bias in selection of the reported result	5.1 Were the data that produced this result analysed in accordance with a pre-specified analysis plan that was finalized before unblinded outcome data were	Y
	5.2 ... multiple eligible outcome measurements (e.g. scales, definitions, time points) within the outcome domain?	Y
	5.3 ... multiple eligible analyses of the data?	NA
	**Risk of bias judgement**	Low
Overall bias	**Risk of bias judgement**	Some concerns
Study	Tanghongjun(2015)	
Domain	Signalling question	Responese
Bias arising from the randomization process	1.1 Was the allocation sequence random?	Y
	1.2 Was the allocation sequence concealed until participants were enrolled and assigned to interventions?	NA
	1.3 Did baseline differences between intervention groups suggest a problem with the randomization process?	NA
	**Risk of bias judgement**	Some concerns
Bias due to deviations from intended interventions	2.1.Were participants aware of their assigned intervention during the trial?	NA
	2.2.Were carers and people delivering the interventions aware of participants' assigned intervention during the trial?	NA
	2.3. If Y/PY/NI to 2.1 or 2.2: Were there deviations from the intended intervention that arose because of the experimental context?	PY
	2.4 If Y/PY to 2.3: Were these deviations likely to have affected the outcome?	Y
	2.5. If Y/PY/NI to 2.4: Were these deviations from intended intervention balanced between groups?	NA
	2.6 Was an appropriate analysis used to estimate the effect of assignment to intervention?	Y
	2.7 If N/PN/NI to 2.6: Was there potential for a substantial impact (on the result) of the failure to analyse participants in the group to which they were randomized?	NI
	**Risk of bias judgement**	Some concerns
Bias due to missing outcome data	3.1 Were data for this outcome available for all, or nearly all, participants randomized?	Y
	3.2 If N/PN/NI to 3.1: Is there evidence that result was not biased by missing outcome data?	PN
	3.3 If N/PN to 3.2: Could missingness in the outcome depend on its true value?	NA
	3.4 If Y/PY/NI to 3.3: Is it likely that missingness in the outcome depended on its true value?	N
	**Risk of bias judgement**	Low
Bias in measurement of the outcome	4.1 Was the method of measuring the outcome inappropriate?	N
	4.2 Could measurement or ascertainment of the outcome have differed between intervention groups?	NA
	4.3 Were outcome assessors aware of the intervention received by study participants?	N
	4.4 If Y/PY/NI to 4.3: Could assessment of the outcome have been influenced by knowledge of intervention received?	PY

	4.5 If Y/PY/NI to 4.4: Is it likely that assessment of the outcome was influenced by knowledge of intervention received?	NA
	**Risk of bias judgement**	Low
Bias in selection of the reported result	5.1 Were the data that produced this result analysed in accordance with a pre-specified analysis plan that was finalized before unblinded outcome data were	Y
	5.2 ... multiple eligible outcome measurements (e.g. scales, definitions, time points) within the outcome domain?	Y
	5.3 ... multiple eligible analyses of the data?	NA
	**Risk of bias judgement**	Low
Overall bias	**Risk of bias judgement**	Some concerns
Study	Jifengying(2014)	
Domain	Signalling question	Responese
Bias arising from the randomization process	1.1 Was the allocation sequence random?	Y
	1.2 Was the allocation sequence concealed until participants were enrolled and assigned to interventions?	Y
	1.3 Did baseline differences between intervention groups suggest a problem with the randomization process?	Y
	**Risk of bias judgement**	Low
Bias due to deviations from intended interventions	2.1.Were participants aware of their assigned intervention during the trial?	NA
	2.2.Were carers and people delivering the interventions aware of participants' assigned intervention during the trial?	NA
	2.3. If Y/PY/NI to 2.1 or 2.2: Were there deviations from the intended intervention that arose because of the experimental context?	PY
	2.4 If Y/PY to 2.3: Were these deviations likely to have affected the outcome?	Y
	2.5. If Y/PY/NI to 2.4: Were these deviations from intended intervention balanced between groups?	NA
	2.6 Was an appropriate analysis used to estimate the effect of assignment to intervention?	Y
	2.7 If N/PN/NI to 2.6: Was there potential for a substantial impact (on the result) of the failure to analyse participants in the group to which they were randomized?	NI
	**Risk of bias judgement**	Some concerns
Bias due to missing outcome data	3.1 Were data for this outcome available for all, or nearly all, participants randomized?	Y
	3.2 If N/PN/NI to 3.1: Is there evidence that result was not biased by missing outcome data?	PN
	3.3 If N/PN to 3.2: Could missingness in the outcome depend on its true value?	NA
	3.4 If Y/PY/NI to 3.3: Is it likely that missingness in the outcome depended on its true value?	N
	**Risk of bias judgement**	Low
Bias in measurement of the outcome	4.1 Was the method of measuring the outcome inappropriate?	N
	4.2 Could measurement or ascertainment of the outcome have differed between intervention groups?	NA
	4.3 Were outcome assessors aware of the intervention received by study participants?	N
	4.4 If Y/PY/NI to 4.3: Could assessment of the outcome have been influenced by knowledge of intervention received?	PY
	4.5 If Y/PY/NI to 4.4: Is it likely that assessment of the outcome was influenced by knowledge of intervention received?	NA
	**Risk of bias judgement**	Low
Bias in selection of the reported result	5.1 Were the data that produced this result analysed in accordance with a pre-specified analysis plan that was finalized before unblinded outcome data were	Y
	5.2 ... multiple eligible outcome measurements (e.g. scales, definitions, time points) within the outcome domain?	Y
	5.3 ... multiple eligible analyses of the data?	NA
	**Risk of bias judgement**	Some concerns
Overall bias	**Risk of bias judgement**	Some concerns
Study	Liuaipeng(2014)	

Domain	Signalling question	Responese
Bias arising from the randomization process	1.1 Was the allocation sequence random?	Y
	1.2 Was the allocation sequence concealed until participants were enrolled and assigned to interventions?	NA
	1.3 Did baseline differences between intervention groups suggest a problem with the randomization process?	NA
	**Risk of bias judgement**	Some concerns
Bias due to deviations from intended interventions	2.1.Were participants aware of their assigned intervention during the trial?	NA
	2.2.Were carers and people delivering the interventions aware of participants' assigned intervention during the trial?	NA
	2.3. If Y/PY/NI to 2.1 or 2.2: Were there deviations from the intended intervention that arose because of the experimental context?	PY
	2.4 If Y/PY to 2.3: Were these deviations likely to have affected the outcome?	Y
	2.5. If Y/PY/NI to 2.4: Were these deviations from intended intervention balanced between groups?	NA
	2.6 Was an appropriate analysis used to estimate the effect of assignment to intervention?	Y
	2.7 If N/PN/NI to 2.6: Was there potential for a substantial impact (on the result) of the failure to analyse participants in the group to which they were randomized?	NI
	**Risk of bias judgement**	Some concerns
Bias due to missing outcome data	3.1 Were data for this outcome available for all, or nearly all, participants randomized?	Y
	3.2 If N/PN/NI to 3.1: Is there evidence that result was not biased by missing outcome data?	PN
	3.3 If N/PN to 3.2: Could missingness in the outcome depend on its true value?	NA
	3.4 If Y/PY/NI to 3.3: Is it likely that missingness in the outcome depended on its true value?	N
	**Risk of bias judgement**	Low
Bias in measurement of the outcome	4.1 Was the method of measuring the outcome inappropriate?	N
	4.2 Could measurement or ascertainment of the outcome have differed between intervention groups?	NA
	4.3 Were outcome assessors aware of the intervention received by study participants?	N
	4.4 If Y/PY/NI to 4.3: Could assessment of the outcome have been influenced by knowledge of intervention received?	PY
	4.5 If Y/PY/NI to 4.4: Is it likely that assessment of the outcome was influenced by knowledge of intervention received?	NA
	**Risk of bias judgement**	Low
Bias in selection of the reported result	5.1 Were the data that produced this result analysed in accordance with a pre-specified analysis plan that was finalized before unblinded outcome data were	Y
	5.2 ... multiple eligible outcome measurements (e.g. scales, definitions, time points) within the outcome domain?	Y
	5.3 ... multiple eligible analyses of the data?	NA
	**Risk of bias judgement**	Low
Overall bias	**Risk of bias judgement**	Some concerns
Study	Zhangjunjing(2017)	
Domain	Signalling question	Responese
Bias arising from the randomization process	1.1 Was the allocation sequence random?	Y
	1.2 Was the allocation sequence concealed until participants were enrolled and assigned to interventions?	NA
	1.3 Did baseline differences between intervention groups suggest a problem with the randomization process?	NA
	**Risk of bias judgement**	Some concerns
Bias due to deviations from intended	2.1.Were participants aware of their assigned intervention during the trial?	NA
	2.2.Were carers and people delivering the interventions aware of participants' assigned intervention during the trial?	NA
	2.3. If Y/PY/NI to 2.1 or 2.2: Were there deviations from the intended intervention that arose because of the experimental context?	PY
	2.4 If Y/PY to 2.3: Were these deviations likely to have affected the outcome?	Y
	2.5. If Y/PY/NI to 2.4: Were these deviations from intended intervention balanced between groups?	NA

from intended interventions	2.6 Was an appropriate analysis used to estimate the effect of assignment to intervention?	Y
	2.7 If N/PN/NI to 2.6: Was there potential for a substantial impact (on the result) of the failure to analyse participants in the group to which they were randomized?	NI
	**Risk of bias judgement**	Some concerns
Bias due to missing outcome data	3.1 Were data for this outcome available for all, or nearly all, participants randomized?	Y
	3.2 If N/PN/NI to 3.1: Is there evidence that result was not biased by missing outcome data?	PN
	3.3 If N/PN to 3.2: Could missingness in the outcome depend on its true value?	NA
	3.4 If Y/PY/NI to 3.3: Is it likely that missingness in the outcome depended on its true value?	N
	**Risk of bias judgement**	Low
Bias in measurement of the outcome	4.1 Was the method of measuring the outcome inappropriate?	N
	4.2 Could measurement or ascertainment of the outcome have differed between intervention groups?	NA
	4.3 Were outcome assessors aware of the intervention received by study participants?	N
	4.4 If Y/PY/NI to 4.3: Could assessment of the outcome have been influenced by knowledge of intervention received?	PY
	4.5 If Y/PY/NI to 4.4: Is it likely that assessment of the outcome was influenced by knowledge of intervention received?	NA
	**Risk of bias judgement**	Low
Bias in selection of the reported result	5.1 Were the data that produced this result analysed in accordance with a pre-specified analysis plan that was finalized before unblinded outcome data were	Y
	5.2 ... multiple eligible outcome measurements (e.g. scales, definitions, time points) within the outcome domain?	NS
	5.3 ... multiple eligible analyses of the data?	NA
	**Risk of bias judgement**	Low
Overall bias	**Risk of bias judgement**	Some concerns
Study	Baoliang(2016)	
Domain	Signalling question	Responese
Bias arising from the randomization process	1.1 Was the allocation sequence random?	Y
	1.2 Was the allocation sequence concealed until participants were enrolled and assigned to interventions?	NA
	1.3 Did baseline differences between intervention groups suggest a problem with the randomization process?	NA
	**Risk of bias judgement**	Some concerns
Bias due to deviations from intended interventions	2.1.Were participants aware of their assigned intervention during the trial?	NA
	2.2.Were carers and people delivering the interventions aware of participants' assigned intervention during the trial?	NA
	2.3. If Y/PY/NI to 2.1 or 2.2: Were there deviations from the intended intervention that arose because of the experimental context?	PY
	2.4 If Y/PY to 2.3: Were these deviations likely to have affected the outcome?	Y
	2.5. If Y/PY/NI to 2.4: Were these deviations from intended intervention balanced between groups?	NA
	2.6 Was an appropriate analysis used to estimate the effect of assignment to intervention?	Y
	2.7 If N/PN/NI to 2.6: Was there potential for a substantial impact (on the result) of the failure to analyse participants in the group to which they were randomized?	NI
	**Risk of bias judgement**	Some concerns
Bias due to missing outcome data	3.1 Were data for this outcome available for all, or nearly all, participants randomized?	Y
	3.2 If N/PN/NI to 3.1: Is there evidence that result was not biased by missing outcome data?	PN
	3.3 If N/PN to 3.2: Could missingness in the outcome depend on its true value?	NA
	3.4 If Y/PY/NI to 3.3: Is it likely that missingness in the outcome depended on its true value?	N
	**Risk of bias judgement**	Low
Bias in	4.1 Was the method of measuring the outcome inappropriate?	N
	4.2 Could measurement or ascertainment of the outcome have differed between intervention groups?	NA

measurement of the outcome	4.3 Were outcome assessors aware of the intervention received by study participants?	N
	4.4 If Y/PY/NI to 4.3: Could assessment of the outcome have been influenced by knowledge of intervention received?	PY
	4.5 If Y/PY/NI to 4.4: Is it likely that assessment of the outcome was influenced by knowledge of intervention received?	NA
	**Risk of bias judgement**	Low
Bias in selection of the reported result	5.1 Were the data that produced this result analysed in accordance with a pre-specified analysis plan that was finalized before unblinded outcome data were	Y
	5.2 ... multiple eligible outcome measurements (e.g. scales, definitions, time points) within the outcome domain?	Y
	5.3 ... multiple eligible analyses of the data?	NA
	**Risk of bias judgement**	Low
Overall bias	**Risk of bias judgement**	Some concerns
Study	Zhaona(2015)	
Domain	Signalling question	Responese
Bias arising from the randomization process	1.1 Was the allocation sequence random?	Y
	1.2 Was the allocation sequence concealed until participants were enrolled and assigned to interventions?	NA
	1.3 Did baseline differences between intervention groups suggest a problem with the randomization process?	NA
	**Risk of bias judgement**	Some concerns
Bias due to deviations from intended interventions	2.1.Were participants aware of their assigned intervention during the trial?	NA
	2.2.Were carers and people delivering the interventions aware of participants' assigned intervention during the trial?	NA
	2.3. If Y/PY/NI to 2.1 or 2.2: Were there deviations from the intended intervention that arose because of the experimental context?	PY
	2.4 If Y/PY to 2.3: Were these deviations likely to have affected the outcome?	Y
	2.5. If Y/PY/NI to 2.4: Were these deviations from intended intervention balanced between groups?	NA
	2.6 Was an appropriate analysis used to estimate the effect of assignment to intervention?	Y
	2.7 If N/PN/NI to 2.6: Was there potential for a substantial impact (on the result) of the failure to analyse participants in the group to which they were randomized?	NI
	**Risk of bias judgement**	Some concerns
Bias due to missing outcome data	3.1 Were data for this outcome available for all, or nearly all, participants randomized?	Y
	3.2 If N/PN/NI to 3.1: Is there evidence that result was not biased by missing outcome data?	PN
	3.3 If N/PN to 3.2: Could missingness in the outcome depend on its true value?	NA
	3.4 If Y/PY/NI to 3.3: Is it likely that missingness in the outcome depended on its true value?	N
	**Risk of bias judgement**	Low
Bias in measurement of the outcome	4.1 Was the method of measuring the outcome inappropriate?	N
	4.2 Could measurement or ascertainment of the outcome have differed between intervention groups?	NA
	4.3 Were outcome assessors aware of the intervention received by study participants?	N
	4.4 If Y/PY/NI to 4.3: Could assessment of the outcome have been influenced by knowledge of intervention received?	PY
	4.5 If Y/PY/NI to 4.4: Is it likely that assessment of the outcome was influenced by knowledge of intervention received?	NA
	**Risk of bias judgement**	Low
Bias in selection of the reported result	5.1 Were the data that produced this result analysed in accordance with a pre-specified analysis plan that was finalized before unblinded outcome data were	Y
	5.2 ... multiple eligible outcome measurements (e.g. scales, definitions, time points) within the outcome domain?	Y
	5.3 ... multiple eligible analyses of the data?	NA
	**Risk of bias judgement**	Low
Overall bias	**Risk of bias judgement**	Some concerns

Study	Lvhuanming(2019)	
Domain	Signalling question	Responese
Bias arising from the randomization process	1.1 Was the allocation sequence random?	Y
	1.2 Was the allocation sequence concealed until participants were enrolled and assigned to interventions?	Y
	1.3 Did baseline differences between intervention groups suggest a problem with the randomization process?	NA
	**Risk of bias judgement**	Low
Bias due to deviations from intended interventions	2.1.Were participants aware of their assigned intervention during the trial?	Y
	2.2.Were carers and people delivering the interventions aware of participants' assigned intervention during the trial?	Y
	2.3. If Y/PY/NI to 2.1 or 2.2: Were there deviations from the intended intervention that arose because of the experimental context?	PY
	2.4 If Y/PY to 2.3: Were these deviations likely to have affected the outcome?	Y
	2.5. If Y/PY/NI to 2.4: Were these deviations from intended intervention balanced between groups?	NA
	2.6 Was an appropriate analysis used to estimate the effect of assignment to intervention?	Y
	2.7 If N/PN/NI to 2.6: Was there potential for a substantial impact (on the result) of the failure to analyse participants in the group to which they were randomized?	NI
	**Risk of bias judgement**	Low
Bias due to missing outcome data	3.1 Were data for this outcome available for all, or nearly all, participants randomized?	Y
	3.2 If N/PN/NI to 3.1: Is there evidence that result was not biased by missing outcome data?	PN
	3.3 If N/PN to 3.2: Could missingness in the outcome depend on its true value?	NA
	3.4 If Y/PY/NI to 3.3: Is it likely that missingness in the outcome depended on its true value?	N
	**Risk of bias judgement**	Low
Bias in measurement of the outcome	4.1 Was the method of measuring the outcome inappropriate?	N
	4.2 Could measurement or ascertainment of the outcome have differed between intervention groups?	NA
	4.3 Were outcome assessors aware of the intervention received by study participants?	N
	4.4 If Y/PY/NI to 4.3: Could assessment of the outcome have been influenced by knowledge of intervention received?	PY
	4.5 If Y/PY/NI to 4.4: Is it likely that assessment of the outcome was influenced by knowledge of intervention received?	NA
	**Risk of bias judgement**	Low
Bias in selection of the reported result	5.1 Were the data that produced this result analysed in accordance with a pre-specified analysis plan that was finalized before unblinded outcome data were	Y
	5.2 ... multiple eligible outcome measurements (e.g. scales, definitions, time points) within the outcome domain?	Y
	5.3 ... multiple eligible analyses of the data?	NA
	**Risk of bias judgement**	Some concerns
Overall bias	**Risk of bias judgement**	Some concerns

Study	Zhuyuemei(2017)	
Domain	Signalling question	Responese
Bias arising from the randomization process	1.1 Was the allocation sequence random?	Y
	1.2 Was the allocation sequence concealed until participants were enrolled and assigned to interventions?	NA
	1.3 Did baseline differences between intervention groups suggest a problem with the randomization process?	NA
	**Risk of bias judgement**	Some concerns
Bias due to deviations	2.1.Were participants aware of their assigned intervention during the trial?	Y
	2.2.Were carers and people delivering the interventions aware of participants' assigned intervention during the trial?	Y
	2.3. If Y/PY/NI to 2.1 or 2.2: Were there deviations from the intended intervention that arose because of the experimental context?	PY
	2.4 If Y/PY to 2.3: Were these deviations likely to have affected the outcome?	Y

deviations from intended interventions	2.5. If Y/PY/NI to 2.4: Were these deviations from intended intervention balanced between groups?	NA
	2.6 Was an appropriate analysis used to estimate the effect of assignment to intervention?	Y
	2.7 If N/PN/NI to 2.6: Was there potential for a substantial impact (on the result) of the failure to analyse participants in the group to which they were randomized?	NI
	**Risk of bias judgement**	Some concerns
Bias due to missing outcome data	3.1 Were data for this outcome available for all, or nearly all, participants randomized?	Y
	3.2 If N/PN/NI to 3.1: Is there evidence that result was not biased by missing outcome data?	PN
	3.3 If N/PN to 3.2: Could missingness in the outcome depend on its true value?	NA
	3.4 If Y/PY/NI to 3.3: Is it likely that missingness in the outcome depended on its true value?	N
	**Risk of bias judgement**	Low
Bias in measurement of the outcome	4.1 Was the method of measuring the outcome inappropriate?	N
	4.2 Could measurement or ascertainment of the outcome have differed between intervention groups?	NA
	4.3 Were outcome assessors aware of the intervention received by study participants?	N
	4.4 If Y/PY/NI to 4.3: Could assessment of the outcome have been influenced by knowledge of intervention received?	PY
	4.5 If Y/PY/NI to 4.4: Is it likely that assessment of the outcome was influenced by knowledge of intervention received?	NA
	**Risk of bias judgement**	Low
Bias in selection of the reported result	5.1 Were the data that produced this result analysed in accordance with a pre-specified analysis plan that was finalized before unblinded outcome data were	Y
	5.2 ... multiple eligible outcome measurements (e.g. scales, definitions, time points) within the outcome domain?	Y
	5.3 ... multiple eligible analyses of the data?	NA
	**Risk of bias judgement**	Some concerns
Overall bias	**Risk of bias judgement**	Some concerns
Study	Lixiaoli(2018)	
Domain	Signalling question	Responese
Bias arising from the randomization process	1.1 Was the allocation sequence random?	Y
	1.2 Was the allocation sequence concealed until participants were enrolled and assigned to interventions?	NA
	1.3 Did baseline differences between intervention groups suggest a problem with the randomization process?	NA
	**Risk of bias judgement**	Some concerns
Bias due to deviations from intended interventions	2.1.Were participants aware of their assigned intervention during the trial?	Y
	2.2.Were carers and people delivering the interventions aware of participants' assigned intervention during the trial?	Y
	2.3. If Y/PY/NI to 2.1 or 2.2: Were there deviations from the intended intervention that arose because of the experimental context?	PY
	2.4 If Y/PY to 2.3: Were these deviations likely to have affected the outcome?	Y
	2.5. If Y/PY/NI to 2.4: Were these deviations from intended intervention balanced between groups?	NA
	2.6 Was an appropriate analysis used to estimate the effect of assignment to intervention?	Y
	2.7 If N/PN/NI to 2.6: Was there potential for a substantial impact (on the result) of the failure to analyse participants in the group to which they were randomized?	NI
	**Risk of bias judgement**	Some concerns
Bias due to missing outcome data	3.1 Were data for this outcome available for all, or nearly all, participants randomized?	Y
	3.2 If N/PN/NI to 3.1: Is there evidence that result was not biased by missing outcome data?	PN
	3.3 If N/PN to 3.2: Could missingness in the outcome depend on its true value?	NA
	3.4 If Y/PY/NI to 3.3: Is it likely that missingness in the outcome depended on its true value?	N
	**Risk of bias judgement**	Low

Bias in measurement of the outcome	4.1 Was the method of measuring the outcome inappropriate?	N
	4.2 Could measurement or ascertainment of the outcome have differed between intervention groups?	NA
	4.3 Were outcome assessors aware of the intervention received by study participants?	N
	4.4 If Y/PY/NI to 4.3: Could assessment of the outcome have been influenced by knowledge of intervention received?	PY
	4.5 If Y/PY/NI to 4.4: Is it likely that assessment of the outcome was influenced by knowledge of intervention received?	NA
	**Risk of bias judgement**	Low
Bias in selection of the reported result	5.1 Were the data that produced this result analysed in accordance with a pre-specified analysis plan that was finalized before unblinded outcome data were	Y
	5.2 ... multiple eligible outcome measurements (e.g. scales, definitions, time points) within the outcome domain?	Y
	5.3 ... multiple eligible analyses of the data?	NA
	**Risk of bias judgement**	Some concerns
Overall bias	**Risk of bias judgement**	Some concerns
Study	Yanghonggui(2015)	
Domain	Signalling question	Responese
Bias arising from the randomization process	1.1 Was the allocation sequence random?	Y
	1.2 Was the allocation sequence concealed until participants were enrolled and assigned to interventions?	NA
	1.3 Did baseline differences between intervention groups suggest a problem with the randomization process?	NA
	**Risk of bias judgement**	Some concerns
Bias due to deviations from intended interventions	2.1.Were participants aware of their assigned intervention during the trial?	NA
	2.2.Were carers and people delivering the interventions aware of participants' assigned intervention during the trial?	Y
	2.3. If Y/PY/NI to 2.1 or 2.2: Were there deviations from the intended intervention that arose because of the experimental context?	PY
	2.4 If Y/PY to 2.3: Were these deviations likely to have affected the outcome?	Y
	2.5. If Y/PY/NI to 2.4: Were these deviations from intended intervention balanced between groups?	NA
	2.6 Was an appropriate analysis used to estimate the effect of assignment to intervention?	Y
	2.7 If N/PN/NI to 2.6: Was there potential for a substantial impact (on the result) of the failure to analyse participants in the group to which they were randomized?	NI
	**Risk of bias judgement**	Some concerns
Bias due to missing outcome data	3.1 Were data for this outcome available for all, or nearly all, participants randomized?	Y
	3.2 If N/PN/NI to 3.1: Is there evidence that result was not biased by missing outcome data?	PN
	3.3 If N/PN to 3.2: Could missingness in the outcome depend on its true value?	NA
	3.4 If Y/PY/NI to 3.3: Is it likely that missingness in the outcome depended on its true value?	N
	**Risk of bias judgement**	Low
Bias in measurement of the outcome	4.1 Was the method of measuring the outcome inappropriate?	N
	4.2 Could measurement or ascertainment of the outcome have differed between intervention groups?	NA
	4.3 Were outcome assessors aware of the intervention received by study participants?	N
	4.4 If Y/PY/NI to 4.3: Could assessment of the outcome have been influenced by knowledge of intervention received?	PY
	4.5 If Y/PY/NI to 4.4: Is it likely that assessment of the outcome was influenced by knowledge of intervention received?	NA
	**Risk of bias judgement**	Low
Bias in selection of the reported result	5.1 Were the data that produced this result analysed in accordance with a pre-specified analysis plan that was finalized before unblinded outcome data were	Y
	5.2 ... multiple eligible outcome measurements (e.g. scales, definitions, time points) within the outcome domain?	Y
	5.3 ... multiple eligible analyses of the data?	NA
	**Risk of bias judgement**	Low

Overall bias	**Risk of bias judgement**	Some concerns
Study	Lijiahong(2015)	
Domain	Signalling question	Responese
Bias arising from the randomization process	1.1 Was the allocation sequence random?	Y
	1.2 Was the allocation sequence concealed until participants were enrolled and assigned to interventions?	NA
	1.3 Did baseline differences between intervention groups suggest a problem with the randomization process?	NA
	**Risk of bias judgement**	Some concerns
Bias due to deviations from intended interventions	2.1.Were participants aware of their assigned intervention during the trial?	NA
	2.2.Were carers and people delivering the interventions aware of participants' assigned intervention during the trial?	NA
	2.3. If Y/PY/NI to 2.1 or 2.2: Were there deviations from the intended intervention that arose because of the experimental context?	PY
	2.4 If Y/PY to 2.3: Were these deviations likely to have affected the outcome?	Y
	2.5. If Y/PY/NI to 2.4: Were these deviations from intended intervention balanced between groups?	NA
	2.6 Was an appropriate analysis used to estimate the effect of assignment to intervention?	Y
	2.7 If N/PN/NI to 2.6: Was there potential for a substantial impact (on the result) of the failure to analyse participants in the group to which they were randomized?	NI
	**Risk of bias judgement**	Some concerns
Bias due to missing outcome data	3.1 Were data for this outcome available for all, or nearly all, participants randomized?	Y
	3.2 If N/PN/NI to 3.1: Is there evidence that result was not biased by missing outcome data?	PN
	3.3 If N/PN to 3.2: Could missingness in the outcome depend on its true value?	NA
	3.4 If Y/PY/NI to 3.3: Is it likely that missingness in the outcome depended on its true value?	N
	**Risk of bias judgement**	Low
Bias in measurement of the outcome	4.1 Was the method of measuring the outcome inappropriate?	N
	4.2 Could measurement or ascertainment of the outcome have differed between intervention groups?	NA
	4.3 Were outcome assessors aware of the intervention received by study participants?	N
	4.4 If Y/PY/NI to 4.3: Could assessment of the outcome have been influenced by knowledge of intervention received?	PY
	4.5 If Y/PY/NI to 4.4: Is it likely that assessment of the outcome was influenced by knowledge of intervention received?	NA
	**Risk of bias judgement**	Low
Bias in selection of the reported result	5.1 Were the data that produced this result analysed in accordance with a pre-specified analysis plan that was finalized before unblinded outcome data were	Y
	5.2 ... multiple eligible outcome measurements (e.g. scales, definitions, time points) within the outcome domain?	Y
	5.3 ... multiple eligible analyses of the data?	NA
	**Risk of bias judgement**	Some concerns
Overall bias	**Risk of bias judgement**	Some concerns
Study	Haobingfeng(2017)	
Domain	Signalling question	Responese
Bias arising from the [illegible]	1.1 Was the allocation sequence random?	Y
	1.2 Was the allocation sequence concealed until participants were enrolled and assigned to interventions?	NA
	1.3 Did baseline differences between intervention groups suggest a problem with the randomization process?	NA

randomization process	**Risk of bias judgement**	Some concerns
Bias due to deviations from intended interventions	2.1.Were participants aware of their assigned intervention during the trial?	NA
	2.2.Were carers and people delivering the interventions aware of participants' assigned intervention during the trial?	NA
	2.3. If Y/PY/NI to 2.1 or 2.2: Were there deviations from the intended intervention that arose because of the experimental context?	PY
	2.4 If Y/PY to 2.3: Were these deviations likely to have affected the outcome?	Y
	2.5. If Y/PY/NI to 2.4: Were these deviations from intended intervention balanced between groups?	NA
	2.6 Was an appropriate analysis used to estimate the effect of assignment to intervention?	Y
	2.7 If N/PN/NI to 2.6: Was there potential for a substantial impact (on the result) of the failure to analyse participants in the group to which they were randomized?	NI
	**Risk of bias judgement**	Some concerns
Bias due to missing outcome data	3.1 Were data for this outcome available for all, or nearly all, participants randomized?	Y
	3.2 If N/PN/NI to 3.1: Is there evidence that result was not biased by missing outcome data?	PN
	3.3 If N/PN to 3.2: Could missingness in the outcome depend on its true value?	NA
	3.4 If Y/PY/NI to 3.3: Is it likely that missingness in the outcome depended on its true value?	N
	**Risk of bias judgement**	Low
Bias in measurement of the outcome	4.1 Was the method of measuring the outcome inappropriate?	N
	4.2 Could measurement or ascertainment of the outcome have differed between intervention groups?	NA
	4.3 Were outcome assessors aware of the intervention received by study participants?	N
	4.4 If Y/PY/NI to 4.3: Could assessment of the outcome have been influenced by knowledge of intervention received?	PY
	4.5 If Y/PY/NI to 4.4: Is it likely that assessment of the outcome was influenced by knowledge of intervention received?	NA
	**Risk of bias judgement**	Low
Bias in selection of the reported result	5.1 Were the data that produced this result analysed in accordance with a pre-specified analysis plan that was finalized before unblinded outcome data were	Y
	5.2 ... multiple eligible outcome measurements (e.g. scales, definitions, time points) within the outcome domain?	Y
	5.3 ... multiple eligible analyses of the data?	NA
	**Risk of bias judgement**	Low
Overall bias	**Risk of bias judgement**	Some concerns
Study	Yanghaizhen(2023)	
Domain	Signalling question	Responese
Bias arising from the randomization process	1.1 Was the allocation sequence random?	Y
	1.2 Was the allocation sequence concealed until participants were enrolled and assigned to interventions?	NA
	1.3 Did baseline differences between intervention groups suggest a problem with the randomization process?	NA
	**Risk of bias judgement**	Some concerns
Bias due to deviations from intended interventions	2.1.Were participants aware of their assigned intervention during the trial?	Y
	2.2.Were carers and people delivering the interventions aware of participants' assigned intervention during the trial?	Y
	2.3. If Y/PY/NI to 2.1 or 2.2: Were there deviations from the intended intervention that arose because of the experimental context?	PY
	2.4 If Y/PY to 2.3: Were these deviations likely to have affected the outcome?	Y
	2.5. If Y/PY/NI to 2.4: Were these deviations from intended intervention balanced between groups?	NA
	2.6 Was an appropriate analysis used to estimate the effect of assignment to intervention?	Y
	2.7 If N/PN/NI to 2.6: Was there potential for a substantial impact (on the result) of the failure to analyse participants in the group to which they were randomized?	NI
	**Risk of bias judgement**	Low
	3.1 Were data for this outcome available for all, or nearly all, participants randomized?	Y

Bias due to missing outcome data	3.2 If N/PN/NI to 3.1: Is there evidence that result was not biased by missing outcome data?	PN
	3.3 If N/PN to 3.2: Could missingness in the outcome depend on its true value?	NA
	3.4 If Y/PY/NI to 3.3: Is it likely that missingness in the outcome depended on its true value?	N
	**Risk of bias judgement**	Low
Bias in measurement of the outcome	4.1 Was the method of measuring the outcome inappropriate?	N
	4.2 Could measurement or ascertainment of the outcome have differed between intervention groups?	NA
	4.3 Were outcome assessors aware of the intervention received by study participants?	N
	4.4 If Y/PY/NI to 4.3: Could assessment of the outcome have been influenced by knowledge of intervention received?	PY
	4.5 If Y/PY/NI to 4.4: Is it likely that assessment of the outcome was influenced by knowledge of intervention received?	NA
	**Risk of bias judgement**	Low
Bias in selection of the reported result	5.1 Were the data that produced this result analysed in accordance with a pre-specified analysis plan that was finalized before unblinded outcome data were	Y
	5.2 ... multiple eligible outcome measurements (e.g. scales, definitions, time points) within the outcome domain?	Y
	5.3 ... multiple eligible analyses of the data?	NA
	**Risk of bias judgement**	Some concerns
Overall bias	**Risk of bias judgement**	Some concerns
TRQI VS Ribavirin (n=5)		
Study	Cuibingzhong(2013)	
Domain	Signalling question	Responese
Bias arising from the randomization process	1.1 Was the allocation sequence random?	Y
	1.2 Was the allocation sequence concealed until participants were enrolled and assigned to interventions?	NA
	1.3 Did baseline differences between intervention groups suggest a problem with the randomization process?	NA
	**Risk of bias judgement**	Some concerns
Bias due to deviations from intended interventions	2.1.Were participants aware of their assigned intervention during the trial?	NA
	2.2.Were carers and people delivering the interventions aware of participants' assigned intervention during the trial?	N
	2.3. If Y/PY/NI to 2.1 or 2.2: Were there deviations from the intended intervention that arose because of the experimental context?	PY
	2.4 If Y/PY to 2.3: Were these deviations likely to have affected the outcome?	Y
	2.5. If Y/PY/NI to 2.4: Were these deviations from intended intervention balanced between groups?	NA
	2.6 Was an appropriate analysis used to estimate the effect of assignment to intervention?	Y
	2.7 If N/PN/NI to 2.6: Was there potential for a substantial impact (on the result) of the failure to analyse participants in the group to which they were randomized?	NI
	**Risk of bias judgement**	Some concerns
Bias due to missing outcome data	3.1 Were data for this outcome available for all, or nearly all, participants randomized?	Y
	3.2 If N/PN/NI to 3.1: Is there evidence that result was not biased by missing outcome data?	PN
	3.3 If N/PN to 3.2: Could missingness in the outcome depend on its true value?	NA
	3.4 If Y/PY/NI to 3.3: Is it likely that missingness in the outcome depended on its true value?	N
	**Risk of bias judgement**	Low
Bias in measurement of the outcome	4.1 Was the method of measuring the outcome inappropriate?	N
	4.2 Could measurement or ascertainment of the outcome have differed between intervention groups?	NA
	4.3 Were outcome assessors aware of the intervention received by study participants?	N
	4.4 If Y/PY/NI to 4.3: Could assessment of the outcome have been influenced by knowledge of intervention received?	PY
	4.5 If Y/PY/NI to 4.4: Is it likely that assessment of the outcome was influenced by knowledge of intervention received?	NA

	**Risk of bias judgement**	Low
Bias in selection of the reported result	5.1 Were the data that produced this result analysed in accordance with a pre-specified analysis plan that was finalized before unblinded outcome data were	Y
	5.2 ... multiple eligible outcome measurements (e.g. scales, definitions, time points) within the outcome domain?	Y
	5.3 ... multiple eligible analyses of the data?	NA
	**Risk of bias judgement**	Some concerns
Overall bias	**Risk of bias judgement**	Some concerns
Study	Wangchunying(2014)	
Domain	Signalling question	Responese
Bias arising from the randomization process	1.1 Was the allocation sequence random?	Y
	1.2 Was the allocation sequence concealed until participants were enrolled and assigned to interventions?	NA
	1.3 Did baseline differences between intervention groups suggest a problem with the randomization process?	NA
	**Risk of bias judgement**	Some concerns
Bias due to deviations from intended interventions	2.1.Were participants aware of their assigned intervention during the trial?	NA
	2.2.Were carers and people delivering the interventions aware of participants' assigned intervention during the trial?	Y
	2.3. If Y/PY/NI to 2.1 or 2.2: Were there deviations from the intended intervention that arose because of the experimental context?	PY
	2.4 If Y/PY to 2.3: Were these deviations likely to have affected the outcome?	Y
	2.5. If Y/PY/NI to 2.4: Were these deviations from intended intervention balanced between groups?	NA
	2.6 Was an appropriate analysis used to estimate the effect of assignment to intervention?	Y
	2.7 If N/PN/NI to 2.6: Was there potential for a substantial impact (on the result) of the failure to analyse participants in the group to which they were randomized?	NI
	**Risk of bias judgement**	Some concerns
Bias due to missing outcome data	3.1 Were data for this outcome available for all, or nearly all, participants randomized?	Y
	3.2 If N/PN/NI to 3.1: Is there evidence that result was not biased by missing outcome data?	PN
	3.3 If N/PN to 3.2: Could missingness in the outcome depend on its true value?	NA
	3.4 If Y/PY/NI to 3.3: Is it likely that missingness in the outcome depended on its true value?	N
	**Risk of bias judgement**	Low
Bias in measurement of the outcome	4.1 Was the method of measuring the outcome inappropriate?	N
	4.2 Could measurement or ascertainment of the outcome have differed between intervention groups?	NA
	4.3 Were outcome assessors aware of the intervention received by study participants?	N
	4.4 If Y/PY/NI to 4.3: Could assessment of the outcome have been influenced by knowledge of intervention received?	PY
	4.5 If Y/PY/NI to 4.4: Is it likely that assessment of the outcome was influenced by knowledge of intervention received?	NA
	**Risk of bias judgement**	Low
Bias in selection of the reported result	5.1 Were the data that produced this result analysed in accordance with a pre-specified analysis plan that was finalized before unblinded outcome data were	Y
	5.2 ... multiple eligible outcome measurements (e.g. scales, definitions, time points) within the outcome domain?	Y
	5.3 ... multiple eligible analyses of the data?	NA
	**Risk of bias judgement**	Some concerns
Overall bias	**Risk of bias judgement**	Some concerns

Study	Wanmin(2016)	
Domain	Signalling question	Responese
Bias arising from the randomization process	1.1 Was the allocation sequence random?	Y
	1.2 Was the allocation sequence concealed until participants were enrolled and assigned to interventions?	NA
	1.3 Did baseline differences between intervention groups suggest a problem with the randomization process?	NA
	**Risk of bias judgement**	Some concerns
Bias due to deviations from intended interventions	2.1.Were participants aware of their assigned intervention during the trial?	Y
	2.2.Were carers and people delivering the interventions aware of participants' assigned intervention during the trial?	Y
	2.3. If Y/PY/NI to 2.1 or 2.2: Were there deviations from the intended intervention that arose because of the experimental context?	PY
	2.4 If Y/PY to 2.3: Were these deviations likely to have affected the outcome?	Y
	2.5. If Y/PY/NI to 2.4: Were these deviations from intended intervention balanced between groups?	NA
	2.6 Was an appropriate analysis used to estimate the effect of assignment to intervention?	Y
	2.7 If N/PN/NI to 2.6: Was there potential for a substantial impact (on the result) of the failure to analyse participants in the group to which they were randomized?	NI
	**Risk of bias judgement**	Low
Bias due to missing outcome data	3.1 Were data for this outcome available for all, or nearly all, participants randomized?	Y
	3.2 If N/PN/NI to 3.1: Is there evidence that result was not biased by missing outcome data?	PN
	3.3 If N/PN to 3.2: Could missingness in the outcome depend on its true value?	NA
	3.4 If Y/PY/NI to 3.3: Is it likely that missingness in the outcome depended on its true value?	N
	**Risk of bias judgement**	Low
Bias in measurement of the outcome	4.1 Was the method of measuring the outcome inappropriate?	N
	4.2 Could measurement or ascertainment of the outcome have differed between intervention groups?	NA
	4.3 Were outcome assessors aware of the intervention received by study participants?	N
	4.4 If Y/PY/NI to 4.3: Could assessment of the outcome have been influenced by knowledge of intervention received?	PY
	4.5 If Y/PY/NI to 4.4: Is it likely that assessment of the outcome was influenced by knowledge of intervention received?	NA
	**Risk of bias judgement**	Low
Bias in selection of the reported result	5.1 Were the data that produced this result analysed in accordance with a pre-specified analysis plan that was finalized before unblinded outcome data were	Y
	5.2 ... multiple eligible outcome measurements (e.g. scales, definitions, time points) within the outcome domain?	Y
	5.3 ... multiple eligible analyses of the data?	NA
	**Risk of bias judgement**	Low
Overall bias	**Risk of bias judgement**	Some concerns

Study	Kongshanshan(2018)	
Domain	Signalling question	Responese
Bias arising from the randomization process	1.1 Was the allocation sequence random?	Y
	1.2 Was the allocation sequence concealed until participants were enrolled and assigned to interventions?	Y
	1.3 Did baseline differences between intervention groups suggest a problem with the randomization process?	NA
	**Risk of bias judgement**	Low
Bias due to deviations from intended interventions	2.1.Were participants aware of their assigned intervention during the trial?	Y
	2.2.Were carers and people delivering the interventions aware of participants' assigned intervention during the trial?	Y
	2.3. If Y/PY/NI to 2.1 or 2.2: Were there deviations from the intended intervention that arose because of the experimental context?	PY
	2.4 If Y/PY to 2.3: Were these deviations likely to have affected the outcome?	Y
	2.5. If Y/PY/NI to 2.4: Were these deviations from intended intervention balanced between groups?	NA
	2.6 Was an appropriate analysis used to estimate the effect of assignment to intervention?	Y

	2.7 If N/PN/NI to 2.6: Was there potential for a substantial impact (on the result) of the failure to analyse participants in the group to which they were randomized?	NI
	**Risk of bias judgement**	Low
Bias due to missing outcome data	3.1 Were data for this outcome available for all, or nearly all, participants randomized?	Y
	3.2 If N/PN/NI to 3.1: Is there evidence that result was not biased by missing outcome data?	PN
	3.3 If N/PN to 3.2: Could missingness in the outcome depend on its true value?	NA
	3.4 If Y/PY/NI to 3.3: Is it likely that missingness in the outcome depended on its true value?	N
	**Risk of bias judgement**	Low
Bias in measurement of the outcome	4.1 Was the method of measuring the outcome inappropriate?	N
	4.2 Could measurement or ascertainment of the outcome have differed between intervention groups?	NA
	4.3 Were outcome assessors aware of the intervention received by study participants?	N
	4.4 If Y/PY/NI to 4.3: Could assessment of the outcome have been influenced by knowledge of intervention received?	PY
	4.5 If Y/PY/NI to 4.4: Is it likely that assessment of the outcome was influenced by knowledge of intervention received?	NA
	**Risk of bias judgement**	Low
Bias in selection of the reported result	5.1 Were the data that produced this result analysed in accordance with a pre-specified analysis plan that was finalized before unblinded outcome data were	Y
	5.2 ... multiple eligible outcome measurements (e.g. scales, definitions, time points) within the outcome domain?	Y
	5.3 ... multiple eligible analyses of the data?	NA
	**Risk of bias judgement**	Some concerns
Overall bias	**Risk of bias judgement**	Some concerns
Study	Guohui(2017)	
Domain	Signalling question	Responese
Bias arising from the randomization process	1.1 Was the allocation sequence random?	Y
	1.2 Was the allocation sequence concealed until participants were enrolled and assigned to interventions?	NA
	1.3 Did baseline differences between intervention groups suggest a problem with the randomization process?	NA
	**Risk of bias judgement**	Some concerns
Bias due to deviations from intended interventions	2.1.Were participants aware of their assigned intervention during the trial?	NA
	2.2.Were carers and people delivering the interventions aware of participants' assigned intervention during the trial?	Y
	2.3. If Y/PY/NI to 2.1 or 2.2: Were there deviations from the intended intervention that arose because of the experimental context?	PY
	2.4 If Y/PY to 2.3: Were these deviations likely to have affected the outcome?	Y
	2.5. If Y/PY/NI to 2.4: Were these deviations from intended intervention balanced between groups?	NA
	2.6 Was an appropriate analysis used to estimate the effect of assignment to intervention?	NI
	2.7 If N/PN/NI to 2.6: Was there potential for a substantial impact (on the result) of the failure to analyse participants in the group to which they were randomized?	NI
	**Risk of bias judgement**	Some concerns
Bias due to missing outcome data	3.1 Were data for this outcome available for all, or nearly all, participants randomized?	Y
	3.2 If N/PN/NI to 3.1: Is there evidence that result was not biased by missing outcome data?	PN
	3.3 If N/PN to 3.2: Could missingness in the outcome depend on its true value?	NA
	3.4 If Y/PY/NI to 3.3: Is it likely that missingness in the outcome depended on its true value?	N
	**Risk of bias judgement**	Low
Bias in measurement	4.1 Was the method of measuring the outcome inappropriate?	N
	4.2 Could measurement or ascertainment of the outcome have differed between intervention groups?	NA
	4.3 Were outcome assessors aware of the intervention received by study participants?	N

of the outcome	4.4 If Y/PY/NI to 4.3: Could assessment of the outcome have been influenced by knowledge of intervention received?	PY
	4.5 If Y/PY/NI to 4.4: Is it likely that assessment of the outcome was influenced by knowledge of intervention received?	NA
	**Risk of bias judgement**	Low
Bias in selection of the reported result	5.1 Were the data that produced this result analysed in accordance with a pre-specified analysis plan that was finalized before unblinded outcome data were	Y
	5.2 ... multiple eligible outcome measurements (e.g. scales, definitions, time points) within the outcome domain?	Y
	5.3 ... multiple eligible analyses of the data?	NA
	**Risk of bias judgement**	Some concerns
Overall bias	**Risk of bias judgement**	Some concerns

Supplementary Table S10: GRADE assessment

Clinical Effectiveness	Direct evidence		Indirect evidence		Network meta-analysis	
comparison	Risk ratio (95% confidence interval)	Quality of evidence	Risk ratio (95% confidence interval)	Quality of evidence	Risk ratio (95% confidence interval)	Quality of evidence
QKLI vs. XYPI	/	/	0.03 (-0.09,0.15)	Low	0.03 (-0.09,0.15)	Low
QKLI vs. RDNI	/	/	0.05 (-0.07,0.17)	Low	0.05 (-0.07,0.17)	Low
QKLI vs. YHNI	/	/	0.05 (-0.07,0.17)	Low	0.05 (-0.07,0.17)	Low
QKLI vs. TRQI	/	/	0.06 (-0.08,0.20)	Low	0.06 (-0.08,0.20)	Low
QKLI vs. Ribavirin	0.21 (0.10,0.33)	very low	Not estimable	Not estimable	0.21 (0.10,0.33)	very low
XYPI vs. RDNI	/	/	0.02 (-0.03,0.06)	Low	0.02 (-0.03,0.06)	Low
XYPI vs. YHNI	/	/	0.02 (-0.03,0.07)	Very low	0.02 (-0.03,0.07)	Very low
XYPI vs. TRQI	/	/	0.03 (-0.06,0.12)	Low	0.03 (-0.06,0.12)	Low
XYPI vs. Ribavirin	0.18 (0.15,0.21)	Low	Not estimable	Not estimable	0.18 (0.15,0.21)	Low
RDNI vs. YHNI	/	/	0.00 (-0.05,0.05)	Very low	0.00 (-0.05,0.05)	Very low
RDNI vs. TRQI	/	/	0.01 (-0.07,0.10)	Low	0.01 (-0.07,0.10)	Low
RDNI vs. Ribavirin	0.16 (0.13,0.20)	Moderate	Not estimable	Not estimable	0.16 (0.13,0.20)	Moderate
YHNI vs. TRQI	/	/	0.01 (-0.07,0.10)	Low	0.01 (-0.07,0.10)	Low
YHNI vs. Ribavirin	0.16 (0.12,0.20)	Very low	Not estimable	Not estimable	0.16 (0.12,0.20)	Very low
TRQI vs. Ribavirin	0.15 (0.07,0.23)	Low	Not estimable	Not estimable	0.15 (0.07,0.23)	Low
Anti-fever time	Direct evidence		Indirect evidence		Network meta-analysis	
comparison	Risk ratio (95% confidence interval)	Quality of evidence	Risk ratio (95% confidence interval)	Quality of evidence	Risk ratio (95% confidence interval)	Quality of evidence

QKLI vs. YHNI	/	/	-0.80 (-2.49,0.90)	Very low	-0.80 (-2.49,0.90)	Very low
QKLI vs. XYPI	/	/	-0.82 (-2.51,0.86)	Low	-0.82 (-2.51,0.86)	Low
QKLI vs. RDNI	/	/	-0.82 (-2.51,0.87)	Low	-0.82 (-2.51,0.87)	Low
QKLI vs. TRQI	/	/	-0.97 (-3.29,1.35)	Very low	-0.97 (-3.29,1.35)	Very low
QKLI vs. Ribavirin	-1.86 (-3.51,-0.21)	Low	Not estimable	Not estimable	-1.86 (-3.51,-0.21)	Low
YHNI vs. XYPI	/	/	-0.03 (-0.56,0.51)	Very low	-0.03 (-0.56,0.51)	Very low
YHNI vs. RDNI	/	/	-0.02 (-0.58,0.54)	Low	-0.02 (-0.58,0.54)	Low
YHNI vs. TRQI	/	/	-0.17 (-1.86,1.51)	Very low	-0.17 (-1.86,1.51)	Very low
YHNI vs. Ribavirin	-1.06 (-1.46,-0.67)	Low	Not estimable	Not estimable	-1.06 (-1.46,-0.67)	Low
XYPI vs. RDNI	/	/	0.00 (-0.53,0.53)	Low	0.00 (-0.53,0.53)	Low
XYPI vs. TRQI	/	/	-0.15 (-1.83,1.53)	Low	-0.15 (-1.83,1.53)	Low
XYPI vs. Ribavirin	-1.04(-1.39,-0.68)	Very low	Not estimable	Not estimable	-1.04(-1.39,-0.68)	Very low
RDNI vs. TRQI	/	/	-0.15 (-1.84,1.54)	Very low	-0.15 (-1.84,1.54)	Very low
RDNI vs. Ribavirin	-1.04(-1.44,-0.64)	Moderate	Not estimable	Not estimable	-1.04(-1.44,-0.64)	Moderate
TRQI vs. Ribavirin	-0.89 (-2.53,0.75)	Moderate	Not estimable	Not estimable	-0.89 (-2.53,0.75)	Moderate
Time to Relief of Cough	Direct evidence		Indirect evidence		Network meta-analysis	
Comparison	Risk ratio (95% confidence interval)	Quality of evidence	Risk ratio (95% confidence interval)	Quality of evidence	Risk ratio (95% confidence interval)	Quality of evidence
TRQI vs. XYPI	/	/	-0.73 (-1.50,0.04)	Very low	-0.73 (-1.50,0.04)	Very low
TRQI vs. YHNI	/	/	-0.73 (-1.52,0.06)	Low	-0.73 (-1.52,0.06)	Low
TRQI vs. RDNI	/	/	-1.11 (-1.89,-0.33)	Very low	-1.11 (-1.89,-0.33)	Very low
TRQI vs. QKLI	/	/	-1.77 (-2.97,-0.58)	Very low	-1.77 (-2.97,-0.58)	Very low

TRQI vs. Ribavirin	-2.19 (-2.93,-1.46)	Moderate	Not estimable	Not estimable	-2.19 (-2.93,-1.46)	Moderate
XYPI vs. YHNI	/	/	0.00 (-0.35,0.35)	Low	0.00 (-0.35,0.35)	Low
XYPI vs. RDNI	/	/	-0.38 (-0.73,-0.03)	Low	-0.38 (-0.73,-0.03)	Low
XYPI vs. QKLI	/	/	-1.04 (-2.01,-0.08)	Low	-1.04 (-2.01,-0.08)	Low
XYPI vs. Ribavirin	-1.46 (-1.69,-1.24)	Low	Not estimable	Not estimable	-1.46 (-1.69,-1.24)	Low
YHNI vs. RDNI	/	/	-0.38(-0.76,0.00)	Very low	-0.38(-0.76,0.00)	Very low
YHNI vs. QKLI	/	/	-1.04(-2.03,-0.06)	Low	-1.04(-2.03,-0.06)	Low
YHNI vs. Ribavirin	-1.46(-1.74,-1.19)	Very low	Not estimable	Not estimable	-1.46(-1.74,-1.19)	Very low
RDNI vs. QKLI	/	/	-0.66 (-1.64,0.31)	Very low	-0.66 (-1.64,0.31)	Very low
RDNI vs. Ribavirin	-1.08(-1.35,-0.82)	Moderate	Not estimable	Not estimable	-1.08(-1.35,-0.82)	Moderate
QKLI vs. Ribavirin	-0.42 (-1.36,0.52)	Moderate	Not estimable	Not estimable	-0.42 (-1.36,0.52)	Moderate
Time to Relief of Sore Throat	Direct evidence		Indirect evidence		Network meta-analysis	
Comparison	Risk ratio (95% confidence interval)	Quality of evidence	Risk ratio (95% confidence interval)	Quality of evidence	Risk ratio (95% confidence interval)	Quality of evidence
YHNI vs. RDNI	/	/	-0.34 (-0.77,0.09)	Very low	-0.34 (-0.77,0.09)	Very low
YHNI vs. XYPI	/	/	-0.49 (-0.89,-0.08)	Very low	-0.49 (-0.89,-0.08)	Very low
YHNI vs. TRQI	/	/	-1.53 (-2.65,-0.41)	Low	-1.53 (-2.65,-0.41)	Low
YHNI vs. Ribavirin	-1.63 (-1.95,-1.32)	Low	Not estimable	Not estimable	-1.63 (-1.95,-1.32)	Low
RDNI vs. XYPI	/	/	-0.14 (-0.53,0.24)	Very low	-0.14 (-0.53,0.24)	Very low
RDNI vs. TRQI	/	/	-1.19 (-2.30,-0.08)	Low	-1.19 (-2.30,-0.08)	Low
RDNI vs. Ribavirin	-1.29 (-1.58,-1.00)	Moderate	Not estimable	Not estimable	-1.29 (-1.58,-1.00)	Moderate
XYPI vs. TRQI	/	/	-1.05 (-2.15,0.06)	Very low	-1.05 (-2.15,0.06)	Very low

XYPI vs. Ribavirin	-1.15(-1.40,-0.89)	Very low	Not estimable	Not estimable	-1.15(-1.40,-0.89)	Very low
TRQI vs. Ribavirin	-0.10 (-1.17,0.97)	Low	Not estimable	Not estimable	-0.10 (-1.17,0.97)	Low
Time to Relief of Runny Nose	Direct evidence		Indirect evidence		Network meta-analysis	
Comparison	Risk ratio (95% confidence interval)	Quality of evidence	Risk ratio (95% confidence interval)	Quality of evidence	Risk ratio (95% confidence interval)	Quality of evidence
YHNI vs. RDNI	/	/	-1.09 (-1.92,-0.26)	Very low	-1.09 (-1.92,-0.26)	Very low
YHNI vs. XYPI	/	/	-1.42 (-2.28,-0.56)	Very low	-1.42 (-2.28,-0.56)	Very low
YHNI vs. TRQI	/	/	-1.47 (-2.35,-0.59)	Low	-1.47 (-2.35,-0.59)	Low
YHNI vs. Ribavirin	-2.11 (-2.82,-1.40)	Low	Not estimable	Not estimable	-2.11 (-2.82,-1.40)	Low
RDNI vs. XYPI	/	/	-0.33 (-0.99,0.32)	Very low	-0.33 (-0.99,0.32)	Very low
RDNI vs. TRQI	/	/	-0.38 (-1.06,0.29)	Low	-0.38 (-1.06,0.29)	Low
RDNI vs. Ribavirin	-1.02 (-1.45,-0.59)	Low	Not estimable	Not estimable	-1.02 (-1.45,-0.59)	Low
XYPI vs. TRQI	/	/	-0.05 (-0.76,0.66)	Low	-0.05 (-0.76,0.66)	Low
XYPI vs. Ribavirin	-0.69(-1.18,-0.20)	Moderate	Not estimable	Not estimable	-0.69(-1.18,-0.20)	Moderate
TRQI vs. Ribavirin	-0.64(-1.16,-0.12)	Moderate	Not estimable	Not estimable	-0.64(-1.16,-0.12)	Moderate
Time to Relief of Stuffy Nose	Direct evidence		Indirect evidence		Network meta-analysis	
Comparison	Risk ratio (95% confidence interval)	Quality of evidence	Risk ratio (95% confidence interval)	Quality of evidence	Risk ratio (95% confidence interval)	Quality of evidence
YHNI vs. XYPI	/	/	-0.08 (-0.83,0.68)	Very low	-0.08 (-0.83,0.68)	Very low
YHNI vs. RDNI	/	/	-0.19 (-0.66,0.27)	Low	-0.19 (-0.66,0.27)	Low
YHNI vs. Ribavirin	-1.22 (-1.54,-0.89)	Very low	Not estimable	Not estimable	-1.22 (-1.54,-0.89)	Very low

XYPI vs. RDNI	/	/	-0.11 (-0.88,0.65)	Very low	-0.11 (-0.88,0.65)	Very low
XYPI vs. Ribavirin	-1.14 (-1.82,-0.45)	Low	Not estimable	Not estimable	-1.14 (-1.82,-0.45)	Low
RDNI vs. Ribavirin	-1.02(-1.36,-0.69)	Low	Not estimable	Not estimable	-1.02(-1.36,-0.69)	Low

Supplementary Table S11: The PRISMA checklist of this meta-analysis

Section and Topic	Item #	Checklist item	Location where item is reported
**TITLE**			
Title	1	Identify the report as a systematic review.	1
**ABSTRACT**			
Abstract	2	See the PRISMA 2020 for Abstracts checklist.	1
**INTRODUCTION**			
Rationale	3	Describe the rationale for the review in the context of existing knowledge.	1
Objectives	4	Provide an explicit statement of the objective(s) or question(s) the review addresses.	1
**METHODS**			
Eligibility criteria	5	Specify the inclusion and exclusion criteria for the review and how studies were grouped for the syntheses.	2
Information sources	6	Specify all databases, registers, websites, organisations, reference lists and other sources searched or consulted to identify studies. Specify the date when each source was last searched or consulted.	3-4
Search strategy	7	Present the full search strategies for all databases, registers and websites, including any filters and limits used.	4
Selection process	8	Specify the methods used to decide whether a study met the inclusion criteria of the review, including how many reviewers screened each record and each report retrieved, whether they worked independently, and if applicable, details of automation tools used in the process.	4

Section and Topic	Item #	Checklist item	Location where item is reported
Data collection process	9	Specify the methods used to collect data from reports, including how many reviewers collected data from each report, whether they worked independently, any processes for obtaining or confirming data from study investigators, and if applicable, details of automation tools used in the process.	4
Data items	10a	List and define all outcomes for which data were sought. Specify whether all results that were compatible with each outcome domain in each study were sought (e.g. for all measures, time points, analyses), and if not, the methods used to decide which results to collect.	5
	10b	List and define all other variables for which data were sought (e.g. participant and intervention characteristics, funding sources). Describe any assumptions made about any missing or unclear information.	5
Study risk of bias assessment	11	Specify the methods used to assess risk of bias in the included studies, including details of the tool(s) used, how many reviewers assessed each study and whether they worked independently, and if applicable, details of automation tools used in the process.	4
Effect measures	12	Specify for each outcome the effect measure(s) (e.g. risk ratio, mean difference) used in the synthesis or presentation of results.	5
Synthesis methods	13a	Describe the processes used to decide which studies were eligible for each synthesis (e.g. tabulating the study intervention characteristics and comparing against the planned groups for each synthesis (item #5)).	5
	13b	Describe any methods required to prepare the data for presentation or synthesis, such as handling of missing summary statistics, or data conversions.	5
	13c	Describe any methods used to tabulate or visually display results of individual studies and syntheses.	5
	13d	Describe any methods used to synthesize results and provide a rationale for the choice(s). If meta-analysis was performed, describe the model(s), method(s) to identify the presence and extent of statistical heterogeneity, and software package(s) used.	5
	13e	Describe any methods used to explore possible causes of heterogeneity among study results (e.g. subgroup analysis, meta-regression).	5
	13f	Describe any sensitivity analyses conducted to assess robustness of the synthesized results.	5

Section and Topic	Item #	Checklist item	Location where item is reported
Reporting bias assessment	14	Describe any methods used to assess risk of bias due to missing results in a synthesis (arising from reporting biases).	4
Certainty assessment	15	Describe any methods used to assess certainty (or confidence) in the body of evidence for an outcome.	5
**RESULTS**			
Study selection	16a	Describe the results of the search and selection process, from the number of records identified in the search to the number of studies included in the review, ideally using a flow diagram.	5
	16b	Cite studies that might appear to meet the inclusion criteria, but which were excluded, and explain why they were excluded.	5-6
Study characteristics	17	Cite each included study and present its characteristics.	7-11
Risk of bias in studies	18	Present assessments of risk of bias for each included study.	12
Results of individual studies	19	For all outcomes, present, for each study: (a) summary statistics for each group (where appropriate) and (b) an effect estimate and its precision (e.g. confidence/credible interval), ideally using structured tables or plots.	12
Results of syntheses	20a	For each synthesis, briefly summarise the characteristics and risk of bias among contributing studies.	13-15
	20b	Present results of all statistical syntheses conducted. If meta-analysis was done, present for each the summary estimate and its precision (e.g. confidence/credible interval) and measures of statistical heterogeneity. If comparing groups, describe the direction of the effect.	13-15
	20c	Present results of all investigations of possible causes of heterogeneity among study results.	13-15
	20d	Present results of all sensitivity analyses conducted to assess the robustness of the synthesized results.	13-15

Section and Topic	Item #	Checklist item	Location where item is reported
Reporting biases	21	Present assessments of risk of bias due to missing results (arising from reporting biases) for each synthesis assessed.	16
Certainty of evidence	22	Present assessments of certainty (or confidence) in the body of evidence for each outcome assessed.	16
**DISCUSSION**			
Discussion	23a	Provide a general interpretation of the results in the context of other evidence.	18-22
	23b	Discuss any limitations of the evidence included in the review.	18-22
	23c	Discuss any limitations of the review processes used.	18-22
	23d	Discuss implications of the results for practice, policy, and future research.	18-22
**OTHER INFORMATION**			
Registration and protocol	24a	Provide registration information for the review, including register name and registration number, or state that the review was not registered.	3
	24b	Indicate where the review protocol can be accessed, or state that a protocol was not prepared.	3
	24c	Describe and explain any amendments to information provided at registration or in the protocol.	3
Support	25	Describe sources of financial or non-financial support for the review, and the role of the funders or sponsors in the review.	23
Competing interests	26	Declare any competing interests of review authors.	23
Availability of data, code and other materials	27	Report which of the following are publicly available and where they can be found: template data collection forms; data extracted from included studies; data used for all analyses; analytic code; any other materials used in the review.	23
